# Recent Advances in the Enantioselective Synthesis of Chiral Amines via Transition Metal-Catalyzed Asymmetric Hydrogenation

**DOI:** 10.1021/acs.chemrev.1c00496

**Published:** 2021-10-22

**Authors:** Albert Cabré, Xavier Verdaguer, Antoni Riera

**Affiliations:** † Institute for Research in Biomedicine (IRB Barcelona), The Barcelona Institute of Science and Technology, Baldiri Reixac 10, Barcelona E-08028, Spain; ‡ Departament de Química Inorgànica i Orgànica, Secció de Química Orgànica, Universitat de Barcelona, Martí i Franquès 1, Barcelona E-08028, Spain

## Abstract

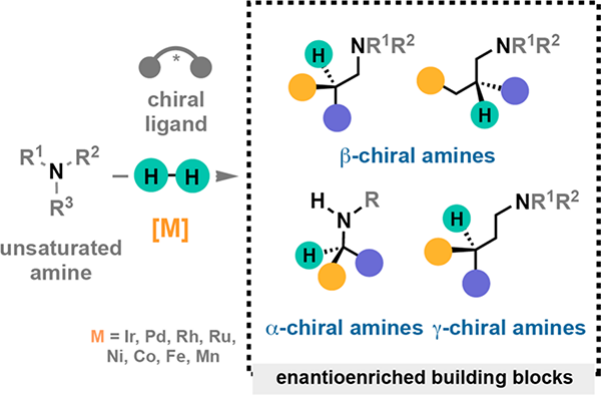

Chiral amines are key structural motifs present in a wide variety of natural products, drugs, and other biologically active compounds. During the past decade, significant advances have been made with respect to the enantioselective synthesis of chiral amines, many of them based on catalytic asymmetric hydrogenation (AH). The present review covers the use of AH in the synthesis of chiral amines bearing a stereogenic center either in the α, β, or γ position with respect to the nitrogen atom, reported from 2010 to 2020. Therefore, we provide an overview of the recent advances in the AH of imines, enamides, enamines, allyl amines, and *N*-heteroaromatic compounds.

## 1 Introduction

**Figure 1:**
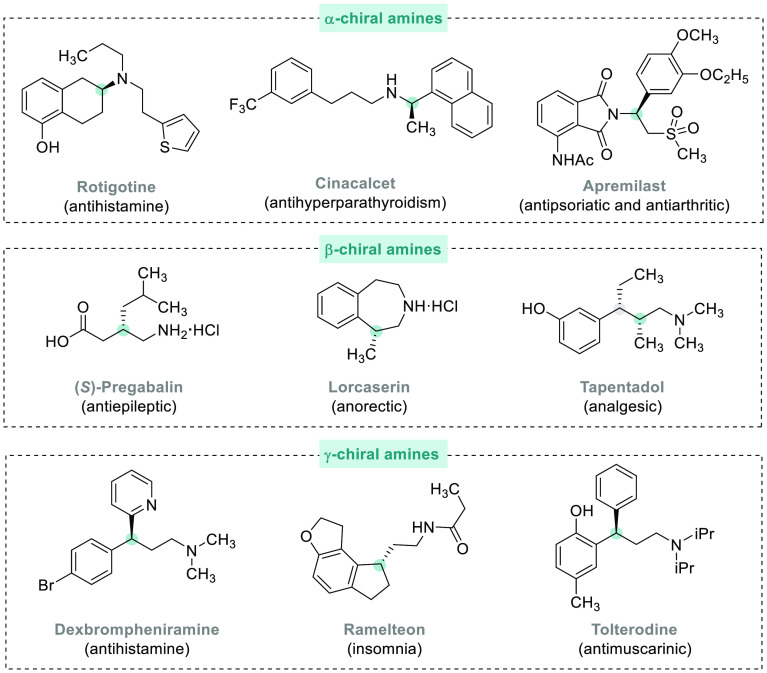
Selected pharmaceuticals with chiral amine fragments.

Chiral amines are key structural motifs present in a wide variety of natural products, drugs, and other biologically active compounds (Figure 1).^1,2^ Around 40–45% of the small molecule pharmaceuticals and many other industrially relevant fine chemicals and agrochemicals contain chiral amine fragments. Moreover, chiral amines can be used as resolving agents, chiral auxiliaries, or building blocks for the asymmetric synthesis of more complex molecules, including natural products. Therefore, the great demand for enantiomerically enriched amines in the life sciences has driven the development of innovative and sustainable synthetic routes toward their efficient preparation.^3^

Despite the widespread relevance of chiral amines, traditional synthetic methods such as resolution are still being used. To overcome their intrinsic limitations, the use of catalytic methods has been widely investigated in recent decades, with asymmetric catalysis being a key research field in modern synthetic chemistry.^4,5^ Although biocatalytic^6^ and organocatalytic^7^ stategies have gained importance, the catalytic approach based on transition metals is still, arguably, the method most widely used.^8^ The design and synthesis of modular chiral ligands have allowed the preparation of novel metal complexes whose properties have been fine-tuned to afford highly active and efficient catalysts.

A relevant number of new metal-catalyzed transformations for the synthesis of chiral amines have been reported. Important achievements have been made in enantioselective methods involving, among others, reductive amination,^9−12^ hydroamination,^13−15^ allylic amination,^16^ or isomerization reactions.^17,18^ The metal-catalyzed enantioselective alkyl addition to imines has also been explored.^19^ Nonetheless, the asymmetric reduction of unsaturated compounds continues to be the most fundamental means of introducing chirality.^20^ In this regard, the asymmetric reduction of imines^21,22^ (via hydrosilylation or transfer hydrogenation, for example) provides an attractive route to chiral amines. However, direct asymmetric hydrogenation (AH) of unsaturated nitrogenated compounds is probably the most powerful and efficient strategy. AH offers excellent atom economy, with almost no waste or byproducts, and thus is a highly sustainable and “green” strategy for attaining optically active amines.^23^ Due to all these advantages, AH has become one of the major disciplines in homogeneous catalysis.^24^ Transition metal-catalyzed AH frequently shows excellent chemo-, regio-, and enantioselectivity, and it is considered a versatile and a reliable tool for the synthesis of chiral drugs.^25^ The AH of challenging organic substrates such as unfunctionalized olefins,^26,27^ nonaromatic cyclic alkenes,^28^ tetrasubstituted olefins,^29^ and (hetero)arenes^30−34^ has been extensively studied, reaching high levels of enantiocontrol. However, and in spite of the long-standing problems being partially solved, many challenges remain. Moreover, the environmental need to use more economical and accessible first-row transition metals (Mn, Fe, Co, and Ni) arises as a new complex task in a field dominated by Rh, Ir, and Ru since its origins.

Focusing on the enantioselective synthesis of chiral amines, important advances have been reported in the last ten years, many of them based on the AH of imines,^35−39^ enamines, and derivatives^39−41^ and *N*-heteroarenes.^30−32^ These advancements are largely driven by a plethora of new chiral phosphorus ligands,^42^ including phosphino-oxazolines^43^ and *P*-stereogenic phosphines.^44,45^ In addition, other chiral phosphine-free metal catalysts, bearing *N*-heterocyclic carbenes^46^ or *C,N-* and *N,N*-based ligands, have also shown outstanding catalytic activity.^47^ Thanks to these breakthroughs, a wide range of previously not easily accessible chiral amines have been obtained with excellent enantioselectivities.

The development of new efficient routes for chiral amine synthesis has a strong and direct impact on medicinal chemistry and the pharmaceutical industry.^48^ Indeed, recent years have witnessed an increase in the synthesis of new chiral amino building blocks due to the great demand for expanding the chemical space in drug discovery platforms.^49^ AH has also found widespread use at the industrial level. The pioneering work of Knowles,^50,51^ Horner,^52^ and Kagan,^53^ followed by the great success of the Monsanto Company^54^ with the production of l-DOPA, opened up industrial-scale synthesis using AH.^55−57^ In 2009, Merck implemented a highly efficient and sustainable enantioselective synthesis of sitagliptin via rhodium-catalyzed AH on a manufacturing scale.^58^ In 2011, Pfizer developed the multikilogram synthesis of the amino acid imagabalin hydrochloride (PD-0332334), used for the treatment of generalized anxiety disorder (GAD), via AH.^59^ Another landmark in the field was the rhodium-catalyzed AH of β-cyanocynnamic esters^60^ to produce pregabalin (Lyrica), which is an important drug for the treatment of fibromyalgia and epilepsy.^61^

**Figure 2:**
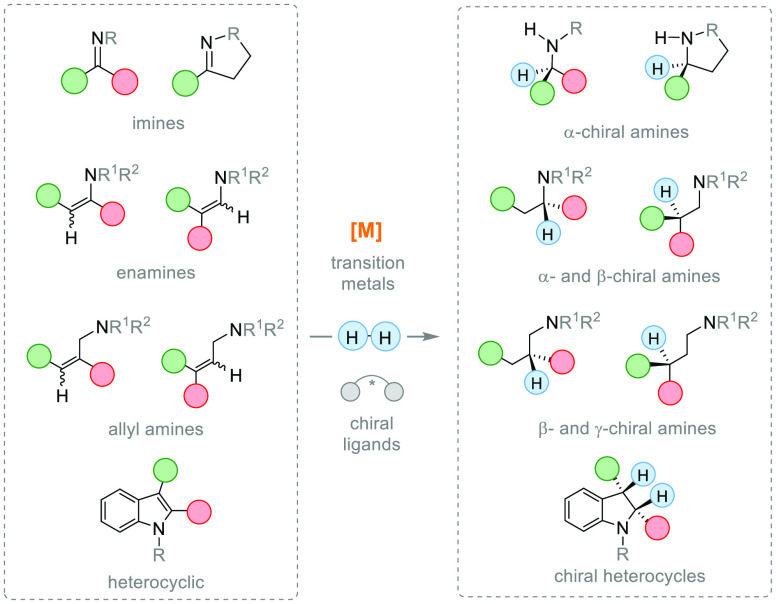
Synthesis of chiral amines via AH of unsaturated compounds using transition metal catalysis.

The present review focuses on the syntheses of chiral amines bearing a stereogenic center in either the α, β, or γ position with respect to the nitrogen atom reported between 2010 and 2020. Therefore, we provide an overview of the recent advances in the AH of the following substrates: (a) imines, (b) enamides, (c) enamines, (d) allyl amines, and (e) *N*-heteroaromatic compounds (Figure 2). Despite the fact that asymmetric reductive amination (ARA) is one of the most convenient methods for the prepration of chiral amines, this topic will not be covered specifically in this review since ARA has been extensively reviewed recently.^10−12^

## 2 Asymmetric Hydrogenation of Imines

The asymmetric hydrogenation of prochiral imines^35−40^ is the most direct and efficient approach to prepare valuable α-chiral amines.^62^ It has been used at industrial scale, exemplified by the multiton-scale production of the herbicide (*S*)-metolachlor.^63^

Imines are more challenging substrates than their oxygenated analogs, namely ketones, due to the easy hydrolysis, the presence of *E,Z* stereoisomers, and nucleophilicity. Thus, extensive efforts have been devoted to the development of efficient synthetic procedures. In recent decades, considerable progress has been made in the AH of both *N*-protected and unprotected^64^ imines. While ruthenium has provided excellent results in asymmetric transfer-hydrogenation reactions, iridium has shown better performance for the direct hydrogenation of imines.^65−67^ In addition, catalytic systems based on earth-abundant metals such as iron or cobalt have started to give competitive results.^68^

### 2.1 *N*-Aryl Imines

#### 2.1.1 *N*-Aryl Aryl Alkyl Imines

**Scheme 1:**
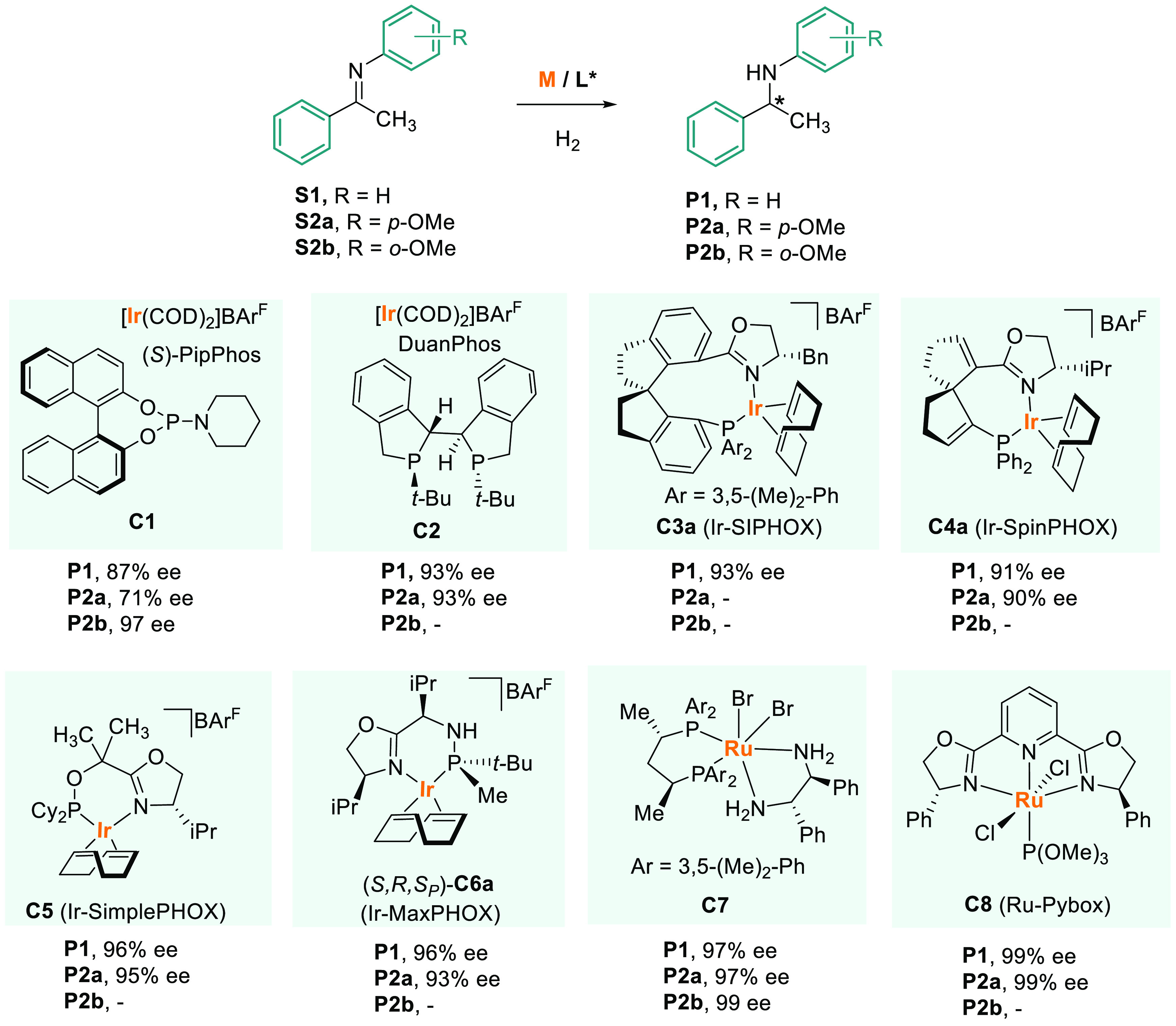
Iridium- and Ruthenium-Catalyzed AH of *N-*Phenyl 1-Phenylethanimine

Several examples of the AH of *N*-aryl imines derived from acetophenones have been reported, reaching excellent levels of enantioselectivity. The reduction of acetophenone phenyl imine (**S1**, Scheme 1) is the standard substrate for this chemistry.

In 2009, de Vries, Feringa, and co-workers reported the iridium-catalyzed AH of *N-*aryl imines using readily available (*S*)-PipPhos as chiral monodentate phosphoramidite ligand (**C1**, Scheme 1).^69^ With the model substrate **S1**, they obtained a product of 87% ee, but the selectivity increased significantly using *ortho*-methoxyphenyl imines (**S2b**) as substrates. This work demonstrated that, although bidentate chiral ligands were considered a superior class in AH, modular monodentate ligands might also be highly efficient in certain cases.^70^

X. Zhang and co-workers used chiral diphosphine DuanPhos in the preparation of iridium catalysts **C2**.^71^ The AH of the standard substrate gave 93% ee. Similar substrates with substitutions in the aryl groups gave 90–93% ee.

Iridium complexes bearing phosphino-oxazoline chiral ligands have been widely used in the AH of *N*-aryl imines.^72,73^ Zhou’s^74^ and Ding’s^75^ groups, respectively, developed chiral complexes with a spiranic backbone **C3a** and **C4a**. Both reported high activity and achieved chiral amines in up to 97% ee. Pfaltz also showed that phosphino-oxazoline ligands provide an excellent platform for the iridium-catalyzed reduction of *N*-aryl imines. In 2010, he reported a range of Ir–P,N chiral complexes (SimplePHOX, **C5**) that were readily accessible by a short and convenient synthesis.^76^ The AH of **S1** with **C5** gave a product of 96% ee. In 2016, Riera and Verdaguer developed a novel family of chiral *P*-stereogenic phosphino-oxazoline ligands called MaxPHOX.^77^ These modular ligands are prepared from three different building blocks: an amino alcohol, an amino acid, and a *P*-stereogenic phosphinous acid.^78^ The key advantage of the Ir-MaxPHOX catalysts (**C6a**) resides in their structural diversity, which is conferred by four possible configurations and diverse substitution patterns. This feature allows fine-tuning of the catalyst for each specific reaction. Moreover, the absolute configuration of the P-center is crucial and has a great impact on catalytic activity. Using these Ir-MaxPHOX complexes, the AH of acyclic *N*-aryl ketimines smoothly proceeded with high enantiocontrol (up to 96% ee) at 1 bar of hydrogen.^79^

Ruthenium catalyst **C7**, first developed by Ohkuma and co-workers in 2012, afforded very high enantioselectivities on the model substrate **S1** (97% ee) (Scheme 1).^80^ The Xyl-Skewphos/DPEN-Ru complex **C7** was applied to the AH of a range of imines derived from aromatic and heteroaromatic ketones with a TON as high as 18,000 to afford chiral amines in up to 99% ee.

Another ruthenium complex, Ru-Pybox (**C8**), developed by Pizzano and Gamasa,^81^ afforded the corresponding amines with excellent enantioselectivities. **C8** gave the best enantioselectivity for the model substrate **S1** (99% ee).

**Scheme 2:**
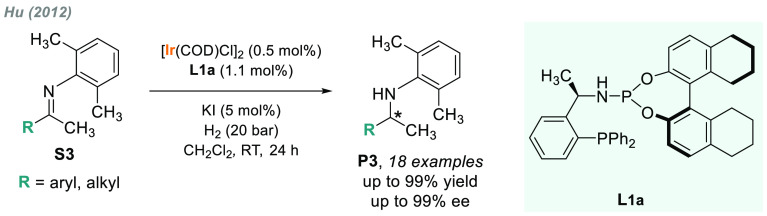
Iridium-Catalyzed AH of Sterically Hindered *N*-Aryl Imines

Sterically hindered *N*-aryl imines are difficult substrates. In 2001, X. Zhang and co-workers described Ir/f-binaphane as an excellent catalyst for the AH of sterically hindered *N*-aryl alkylarylamines.^82^ Later, in 2012, Hu reported an extended substrate scope by using the iridium complex derived from phosphine-phosphoramidite ligand **L1a** (Scheme 2).^83,84^ The corresponding chiral amines **P3**, which are important building blocks in organic synthesis and agrochemistry, were obtained in good to excellent enantioselectivities.

#### 2.1.2 *N*-Aryl Dialkyl Imines

**Scheme 3:**
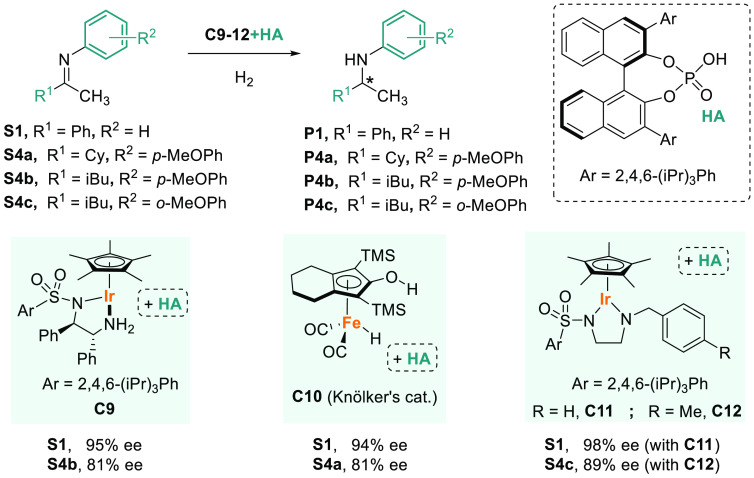
AH of *N*-Aryl Dialkyl Imines Using Binary Catalysts of a Metal Complex and a Chiral Phosphoric Acid (HA)

In contrast to aromatic imines, successful examples of the AH of imines derived from aliphatic ketones (**S4**) are rare and usually with low chiral induction. In 2008, Xiao pioneered the field with the highly efficient cooperative catalysis between the ruthenium complex **C9** and achiral phosphoric acid (HA) (Scheme 3).^85^ In 2011, Beller and co-workers demonstrated that performing ligand-free AHs without the use of precious metal catalysts was possible.^86^ The combination of an achiral iron complex (Knölker’s catalyst, **C10**) with HAs enabled smooth hydrogenation for a wide range of *N*-aryl imines, including **S1** and the dialkyl imine **S4a**. Similarly, in 2013, Xiao reported a family of achiral iridium-(Cp*) complexes containing diamine ligands that, in combination with a chiral HA, gave access to highly active catalysts (**C11**–**C12**) for the AH of *N*-aryl imines derived from both aryl and aliphatic ketones.^87,88^ While **C11** was chosen as the best catalyst for **S1** (98% ee, Scheme 3), for imine **S4c** the highest enantioselectivity was observed using **C12**, which is the best catalyst reported to date for these substrates.

#### 2.1.3 *N*-Aryl α-Imino Esters

The synthesis of enantiomerically pure α-amino acids and their derivatives is of great importance in pharmaceutical and synthetic chemistry.^89^ Chiral α-aryl glycines, in particular, have found wide applicability in the synthesis of significant antibacterial and cardiovascular drugs, such as amoxicillins and nocardicins.^90^ Several highly efficient asymmetric catalytic methods such as the asymmetric Strecker^91^ or Sharpless aminohydroxylation have been developed.^92^ Despite being a logical approach, the AH of the corresponding α-imino esters has scarcely been addressed, presumably because of the relatively poor reactivity of these types of imino substrates toward hydrogenation.

**Scheme 4:**
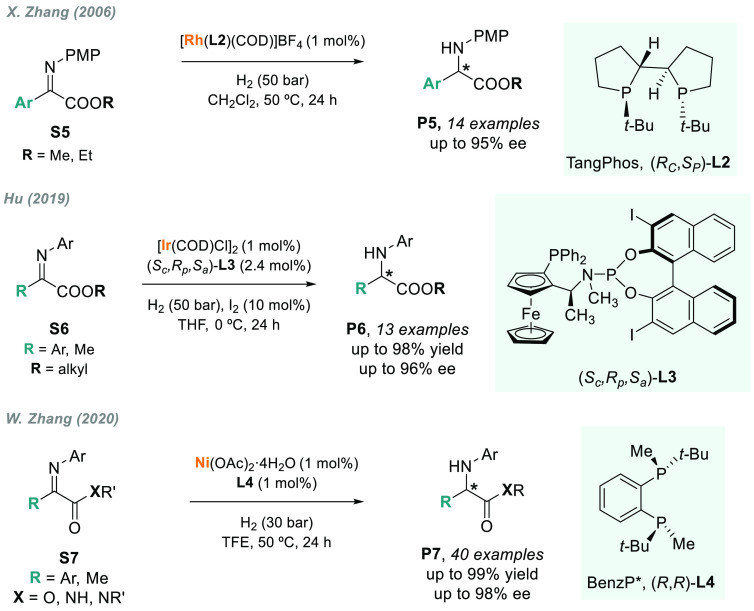
AH of *N*-Aryl α-Imino Esters

In 2006, X. Zhang and co-workers reported the first rhodium-catalyzed AH of **S5** using a *P*-stereogenic diphosphine **L2** (TangPhos), providing chiral glycines **P5** with high yields and enantioselectivities (Scheme 4).^93^ However, the scope of this method was limited to *p*-methoxyphenyl (PMP)-protected α-imino esters. To overcome this constraint, Hu described the iridium-catalyzed AH of α-imino esters **S6** with unsymmetrical hybrid chiral ferrocenylphosphine-phosphoramidite ligand **L3** for the synthesis of optically active α-aryl glycines **P6** (Scheme 4).^94^ The method features high asymmetric induction (up to 96% ee), with the iodo-substituent of the binaphthyl unit playing a fundamental role in the enantioselectivity.

To avoid the use of precious metals, in 2020, W. Zhang and co-workers reported an efficient nickel-catalyzed AH of *N*-aryl imino esters **S7**, affording chiral α-aryl glycines **P7** in high yields and enantioselectivities (up to 98% ee) using a *P*-stereogenic dialkyl phosphine ligand, BenzP* **L4** (Scheme 4).^95^ The reaction was performed on a gram scale at a low catalyst loading (S/C up to 2000). The preparation of two synthetic drug intermediates showcased the applicability of the method.

#### 2.1.4 Exocyclic *N*-Aryl Imines

**Scheme 5:**
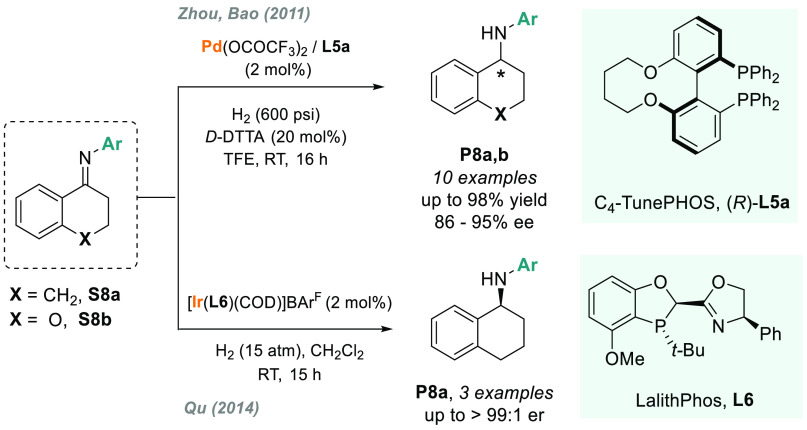
AH of Exocyclic *N*-Aryl Imines

Typically, the AH of exocyclic ketimines derived from 1-tetralone or 4-chromanone exhibited low enantioselectivities, presumably due to the conformational strain upon metal coordination.^96,97^ In 2011, Zhou and Bao reported a highly enantioselective palladium-catalyzed hydrogenation using a catalytic amount of a Brønsted acid as activator (*D*-DTTA).^98^ By using C_4_-TunePhos **L5a** as a chiral ligand, this catalytic system provided straightforward access to enantioenriched cyclic amines **P8a** and **P8b** (86–95% ee, Scheme 5), which are privileged structural motifs present in a large number of drugs and natural compounds.^99^ Iridium-based catalytic systems were also used in this transformation. First, Bolm’s group made a significant advance in the iridium-catalyzed AH of exocylic imine **S8a**. They introduced a novel class of C_1_-symmetry sulfoximines as chiral ligands that, once coordinated, yielded the corresponding chiral amine adduct in 91% ee.^100^ Although it was a single example, the catalytic system also gave excellent results for acyclic *N*-aryl imines. Later, in 2014, Qu and co-workers reported a family of air-stable *P*-stereogenic dihydrobenzooxaphosphole oxazoline ligands (LalithPhos).^101^ In particular, Ir/**L6** was chosen as the best catalyst for the AH of **S8a**, which afforded up to three examples of **P8a** in enantiopure form (Scheme 5).

### 2.2 *N*-Alkyl Imines

**Scheme 6:**
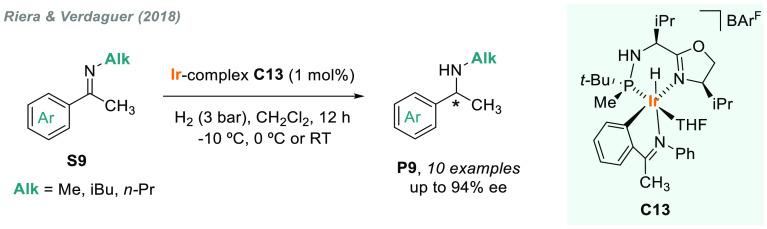
AH of *N*-Methyl and *N*-Alkyl Imines Using Ir(III)-H Complex

Chiral *N*-methyl or *N*-alkyl amine is a frequent pharmacophore in many pharmaceuticals and drugs, and despite other successful approaches,^102,103^ direct AH is the most convenient process. In sharp contrast with the good results obtained with *N*-aryl ketimines, the development of the AH of *N*-alkyl ketimines has been more difficult. The high basicity and nucleophilicity of the corresponding *N*-alkyl amines as reaction products often results in catalyst deactivation. Pfaltz pioneered the use of Ir-PHOX catalysts for the AH of the *N*-methyl imine of acetophenone, albeit with low enantioselectivity.^73^ Later, in 2013, he discovered that the catalyst in the hydrogenation of *N*-aryl imine is actually an iridacycle that forms upon reaction with the imine substrate.^104^ Prompted by this finding, and inspired by the excellent activity that Ir-MaxPHOX catalysts showed in the AH of *N*-aryl imines,^79^ Riera and Verdaguer’s laboratory recently reported a highly efficient AH of *N*-alkyl imines **S9** using iridacycle **C13** prepared fromMaxPHOX and the phenyl imine of benzophenone (Scheme 6).^105^ This catalyst allowed the first direct hydrogenation of methyl and alkyl imines derived from acetophenones in very mild conditions and in high enantioselectivity (up to 94% ee).

**Scheme 7:**
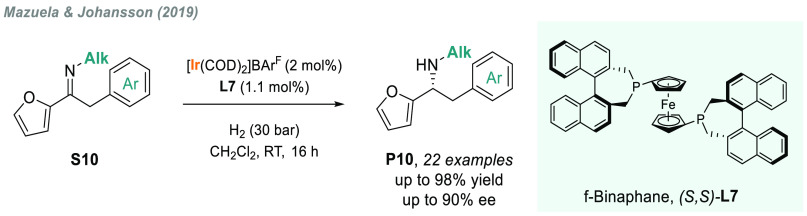
Iridium-Catalyzed AH of *N*-Alkyl α-Aryl Furan-Containing Imines

The AH of *N*-alkyl α-aryl furan-containing imines is a straightforward route to a wide range of unnatural *N*-alkyl aryl alanines. In this regard, Mazuela et al. reported that, using a Ir/(*S,S*)-f-Binaphane-**L7** as catalyst, up to 22 *N*-alkyl imines were efficiently hydrogenated, providing chiral amines **P10** (up to 90% ee), which can be further easily transformed into amino acids (Scheme 7).^106^ The effect of substituents on the nitrogen was remarkable, as the use of large alkyl substituents led to a significant decrease of enantioselectivity.

**Scheme 8:**
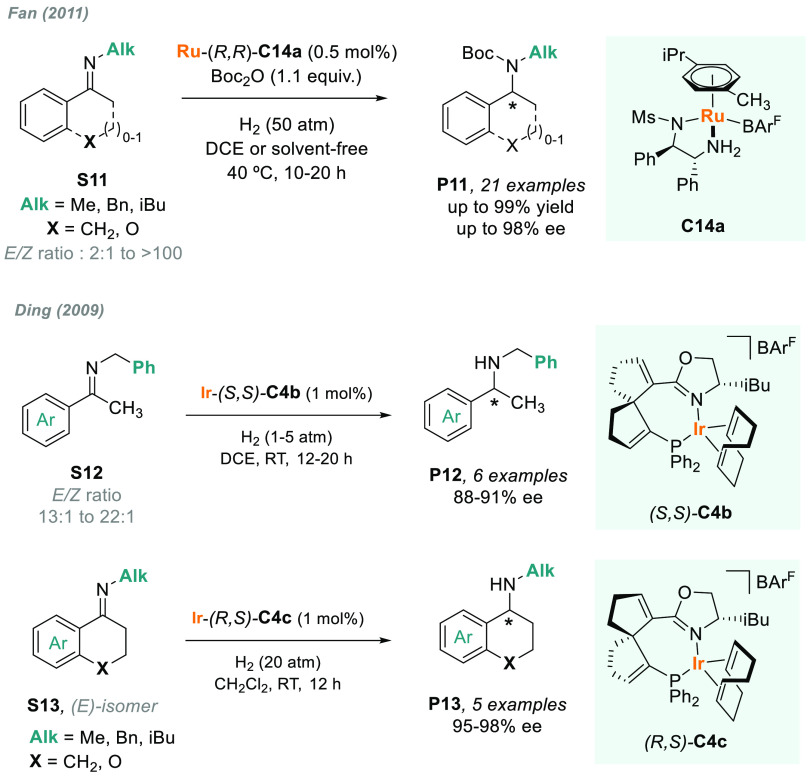
Metal-Catalyzed AH of *N*-Alkyl Imines

Fan described the phosphine-free, chiral, cationic Ru/MsDPEN complex **C14a** which was a highly active catalyst for the AH of a range of acyclic and exocyclic *N*-alkyl ketimines (Scheme 8).^107^ By using BAr^F^ as counterion, a broad range of often problematic substrates **S11** were efficiently hydrogenated with low catalyst loadings, albeit with the use of Boc_2_O to avoid catalyst inhibition. Moreover, this system also operates under solvent-free conditions, thus providing a highly sustainable platform to optically active amines **P11**. The same group later reported a similar ruthenium complex that, together with a phosphoric acid as additive via cooperative catalysis, was also an efficient catalyst for the hydrogenation of *N*-alkyl ketimines **S11** in the absence of Boc_2_O.^108^

Previously, in 2009, Ding and co-workers designed a new family of spiro phosphino-oxazoline chiral ligands that were successfully applied to the iridium-catalyzed AH of *N*-aryl imines.^75^ Of note, the catalytic system is also applicable for *N*-alkyl imines (Scheme 8). Actually, both acyclic (**S12**) and exocyclic (**S13**) imines were efficiently hydrogenated with high levels of enantioselectivity using two distinct precatalysts (diastereoisomers **C4b** and **C4c**, respectively) and without the need of additives.

**Scheme 9:**
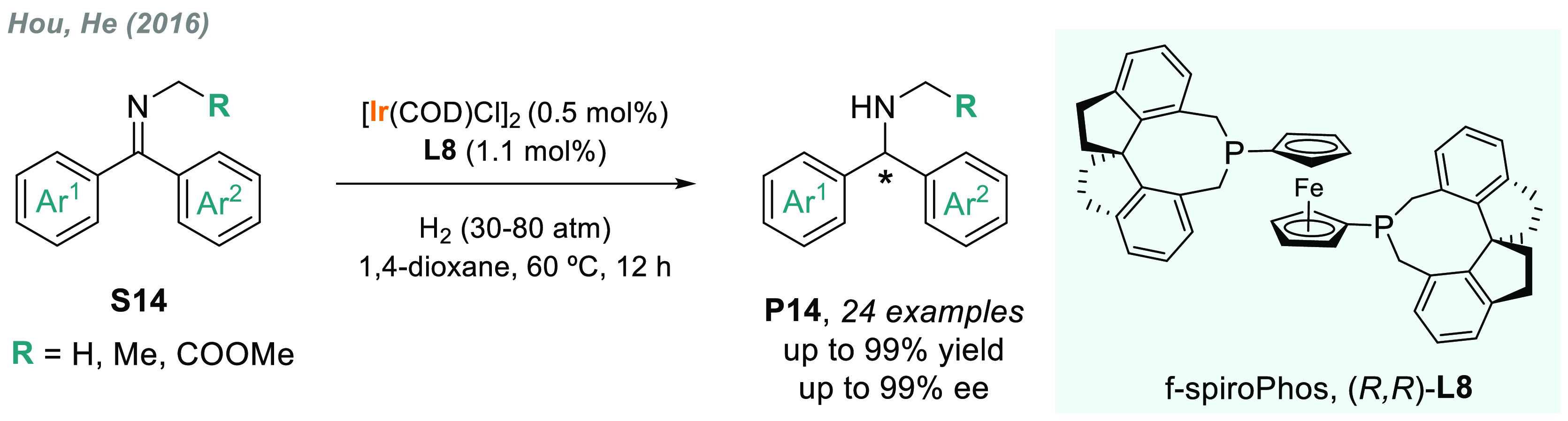
Iridium-Catalyzed AH of Diaryl *N*-Alkyl Imines

Phosphine ligands containing spiro scaffolds^109^ such as f-spiroPhos **L8**, first reported by Hou and co-workers,^110^ emerged as a new and powerful class of chiral ligands for asymmetric catalysis. In 2016, this group reported a highly efficient AH of diarylmethanimines, which are challenging substrates due to the difficulties to distinguish between two sterically similar aryl groups (Scheme 9).^111^ Hou detailed that, by using Ir/**L8** as catalyst, a variety of chiral diarylmethylamines **P14** were obtained with excellent enantioselectivities (up to 99% ee) and high TON. Previously, **L8** had also been successfully applied to the rhodium-catalyzed AH of α,β-unsaturated nitriles,^112^ among other examples.

### 2.3 Cyclic *N*-Aryl Imines

**Scheme 10:**
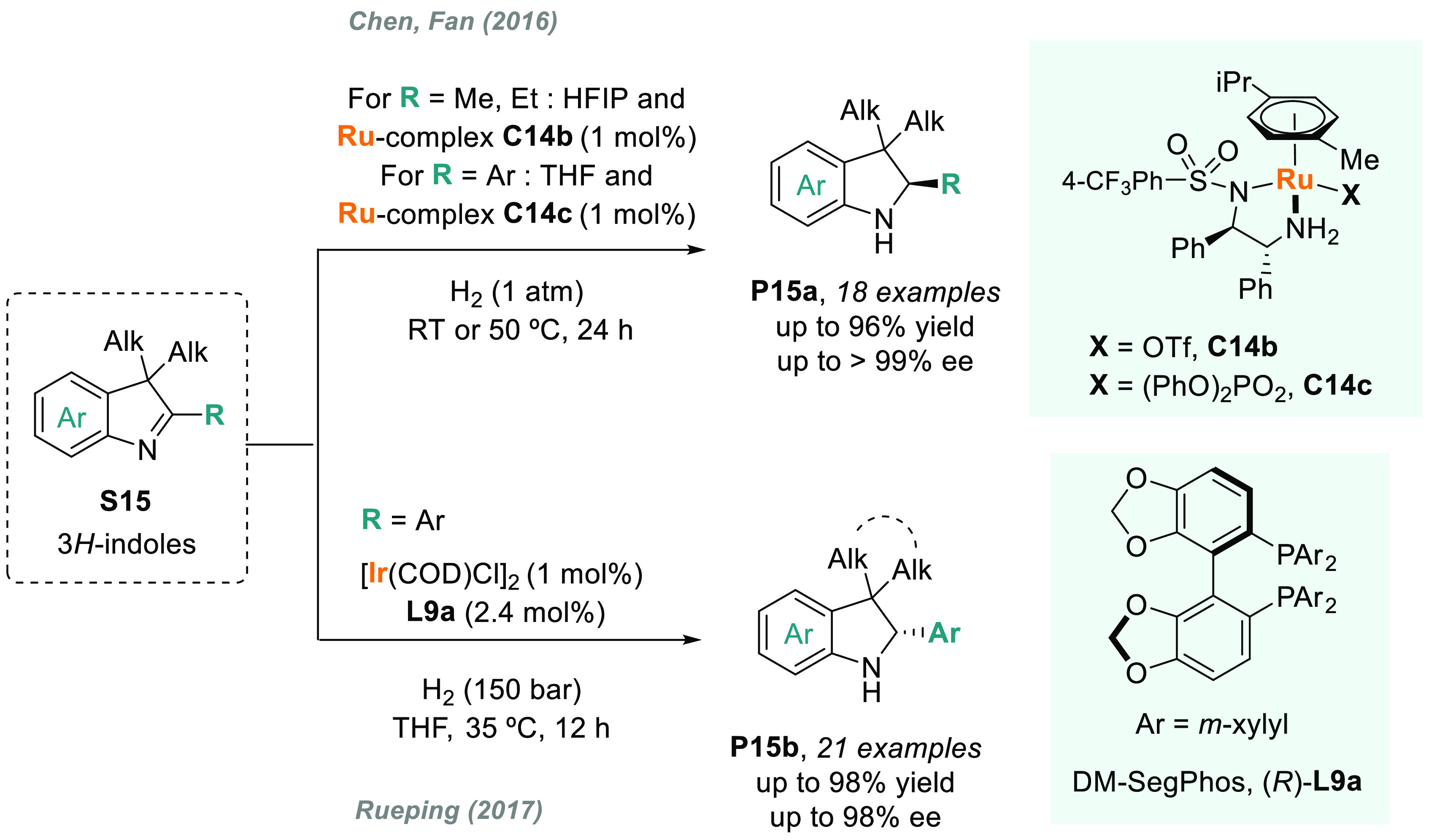
Metal-Catalyzed AH of 3*H*-Indoles

The AH of *N*-heteroarenes is one of the most important ways to access chiral *N*-heterocyclic compounds (see section 6). For instance, a direct strategy to obtain chiral indolines would be the direct AH of the corresponding indoles. However, indoles are a challenging class of substrates and their AH was unsuccessful for many years.^113^ In this field, Fan and Rueping’s groups simultaneously reported two independent catalytic systems that were highly efficient for the AH of 3*H*-indoles (Scheme 10). Fan and co-workers described a highly efficient enantioselective synthesis of 2-alkyl and 2-aryl indolines (**P15a**) via AH using Ru diamine catalysts **C14b** and **C14c**, respectively.^114^ The catalytic reaction proceeded smoothly at low H_2_ pressure and with a high enantioselectivity (>99% ee in the best cases). Both the counteranion and the solvent played a crucial role in catalytic performance. On the other hand, Rueping reported a highly enantioselective iridium-catalyzed AH of 3*H*-indoles **S15** by using chiral diphosphine ligand **L9a**.^115^ A wide range of valuable disubstituted and spirocyclic 2-aryl indolines **P15b** were prepared in excellent results, albeit at elevated H_2_ pressure. Previously, the same group provided an operationally simple route to other biologically relevant heterocyclic compounds, such as dihydrobenzodiazepines, by AH.^116^

**Scheme 11:**
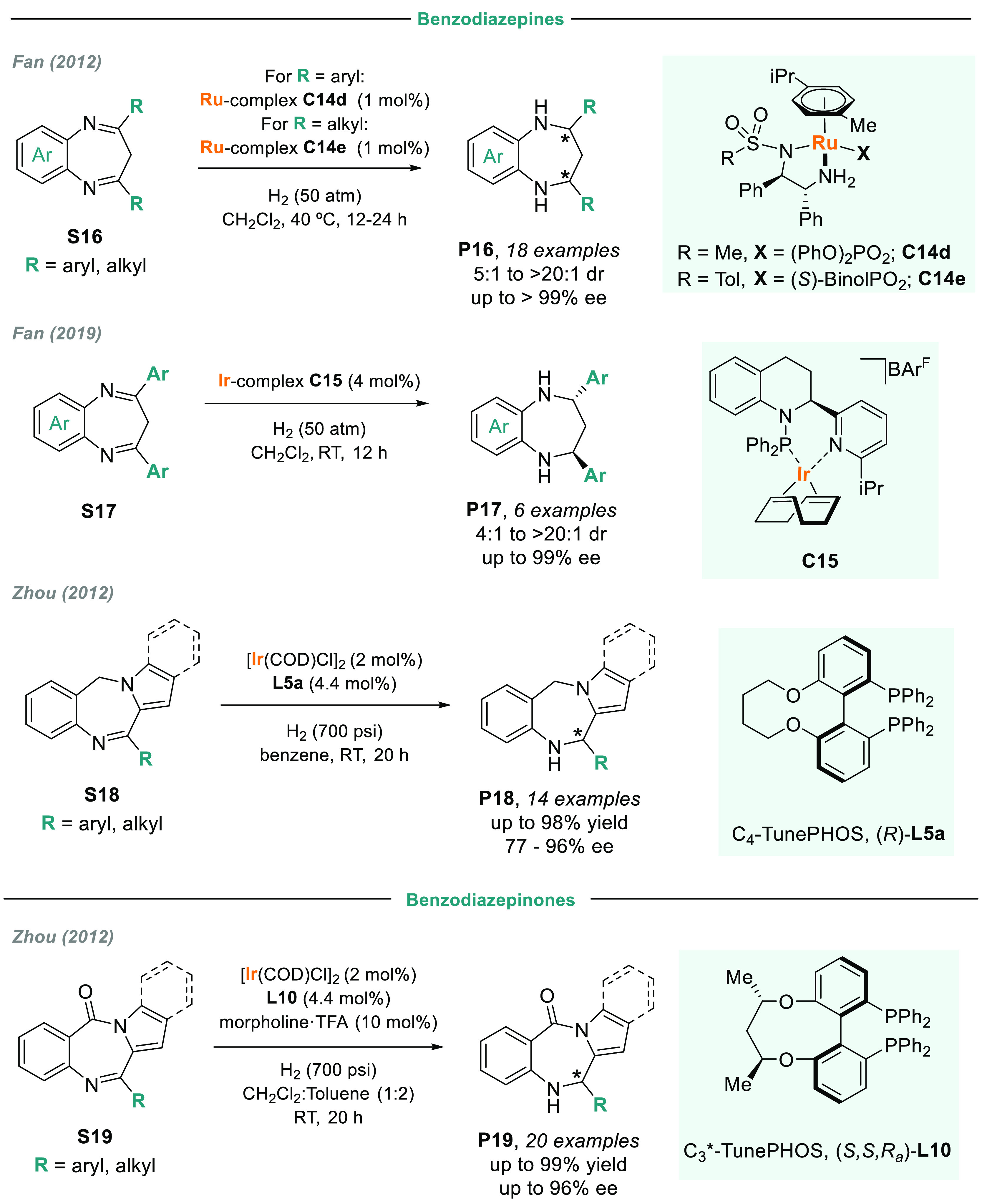
Metal-Catalyzed AH of Benzodiazepines and Benzodiazepinones

The enantioselective synthesis of seven-membered *N*-containing heterocycles has attracted considerable attention during recent decades, as they are versatile pharmacophores in medicinal chemistry. In 2012, Fan and co-workers reported that two Ru/diamine catalysts were highly efficient in the AH of benzodiazepines **S16** (Scheme 11).^117^ Interestingly, an achiral anion influenced both the nature and the coordination effect and reversed the sense of the asymmetric induction. After an exhaustive catalyst screening, **C14d** was chosen for benzodiazepine-bearing aryl substituents, while **C14e** was used for alkyl groups. In the first case, both enantiomers were obtained using the same ligand but in the presence of different achiral counteranions. Recently, they also reported that the iridium complex **C15** is a highly active catalyst for the AH of benzodiazepines **S17** bearing aryl substituents (Scheme 11).^118^ For both catalytic systems, the corresponding optically active dihydrobenzodiazepines were obtained with good to excellent diastereoselectivity and excellent enantioselectivity. The same group reported other catalysts, including dendritic phosphinooxazoline iridium complexes, which proved highly efficient for both the partial and total AH of benzodiazepines.^119−121^

Zhou’s group used Ir chiral complexes also for the AH of benzodiazepines. They reported that Ir/C_4_-TunePhos-**L5a** is a highly active catalytic system for the AH of both pyrrole- and indole-fused benzodiazepines **S18**, reporting a moderate to excellent level of enantioselectivity (Scheme 11).^122^ Moreover, by switching to chiral ligand **L10**, outstanding results were also obtained for the AH of benzodiazepinones **S19**, thus offering a highly versatile catalytic approach for a range of chiral cyclic amines present in numerous important natural products and drugs. In addition, the same group later reported an iridium-catalyzed AH/oxidative fragmentation cascade for the synthesis of chiral dihydrobenzodiazepinones.

**Scheme 12:**
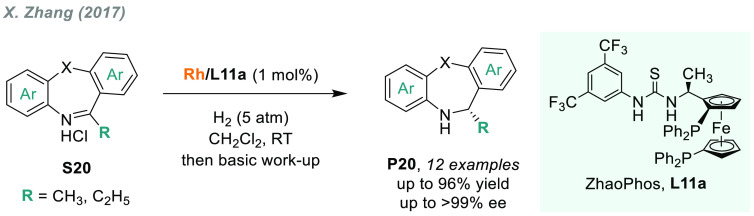
Access to Chiral Seven-Membered Cyclic Amines via Rhodium-Catalyzed AH

A number of successful examples of the AH of some benzo-fused seven-membered cyclic imines for the preparation of chiral benzazepines and benzodiazepines have recently been reported.^123−126^ X. Zhang and co-workers described a highly efficient AH of dibenzoazepine hydrochlorides **S20** catalyzed by Rh/ZhaoPhos-**L11a** (Scheme 12).^127^ The corresponding chiral seven-member cyclic amines **P20** were obtained in high yields and excellent enantioselectivities (>99% ee in the best cases). Interestingly, control experiments revealed that the anion-bonding interaction between the chloride ion of the substrate and the thiourea motif of **L11a** played a key role in enantioselectivity. The same reaction conditions were also useful for the AH of oxazepines.

**Scheme 13:**
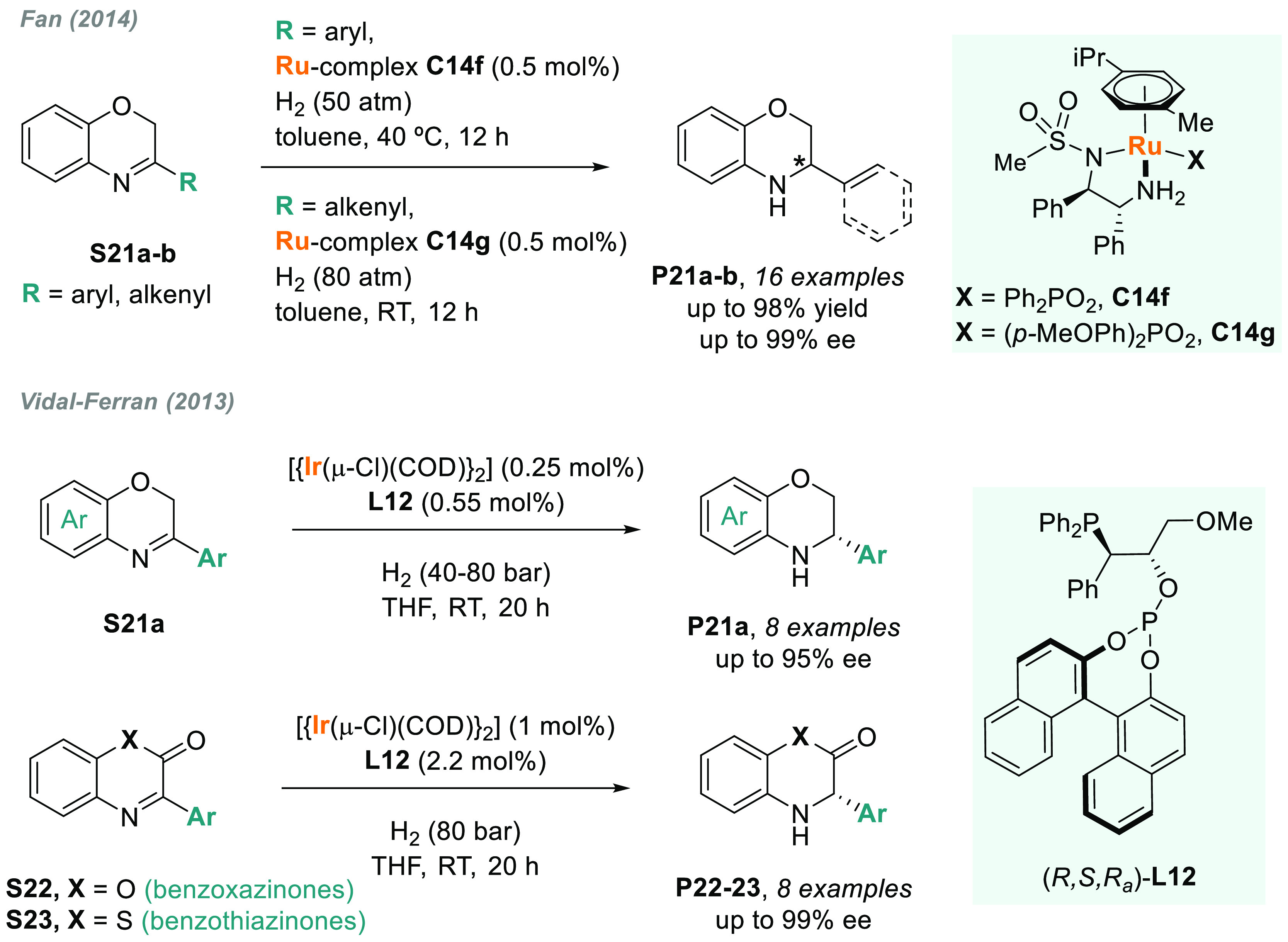
Metal-Catalyzed AH of Benzoxazines and Benzoxazinones

Another important family of C=N-containing heterocycles are benzoxazines and derivatives (**S21–S22**). At the beginning of this decade, Beller,^128^ Ohkuma,^129^ and Zhou’s^130^ groups reported advances in the transition metal-catalyzed AH of this class of compounds. Later, in 2014, Fan expanded the catalytic application of Ru/MsDPEN complexes. In fact, **C14f** and **C14g** were excellent catalysts for the highly enantioselective AH of 3-aryl- and 3-styryl-substituted benzoxazines **S21**, respectively (up to 99% ee, Scheme 13).^131^ In contrast to previous work^130^ where 3-styryl-substituted benzoxazines were completely hydrogenated, this catalytic system showed an exquisite 1,2-selectivity with an appropriate counterion (**C14g**). On the other hand, the main drawback of this method is that *ortho*-substituted aryl substituents in benzoxazines **S21** were not compatible. Unfortunately, when using these substrates, the reaction could not take place, probably due to undesired steric effects. In addition, the AH of 3-alkyl-substituted benzoxazines is underdeveloped.^129^

Moving to iridium catalysis, Vidal-Ferran designed a new phosphine-phosphite ligand **L12**, which, once coordinated to iridium, provided a highly active Ir(I) catalyst for the AH of 3-aryl-substituted benzoxazines (**S21a**), benzoxazinones (**S22**), and benzothiazinones (**S23**) (up to 99% ee, Scheme 13).^132,133^

**Scheme 14:**
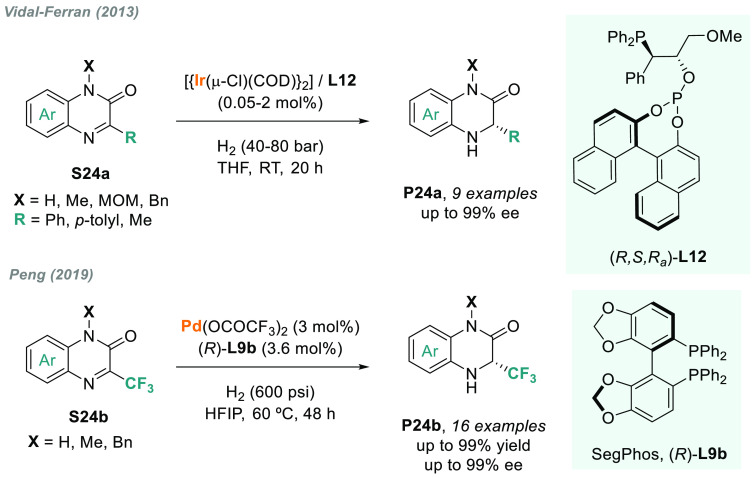
Metal-Catalyzed AH of Quinoxalinones

The iridium catalyst with **L12** was also the first-ever reported catalyst for the AH of quinoxalinones and *N*-substituted quinoxalinones **S24a** (Scheme 14). More recently, Peng and co-workers reported a highly enantioselective palladium-catalyzed AH of **S24b**.^134^ Using (*R*)-SegPhos **L9b** as the chiral ligand, and performing the reaction in HFIP, a wide array of optically active 3-trifluoromethylated dihydroquinoxalinones **P24** were synthesized (>99% ee in the best cases, Scheme 14). However, the substituent on the aromatic ring impaired the reaction. In this regard, the introduction of a methyl group at the 5-position on the phenyl ring inhibited the reaction due to the steric effect.

**Scheme 15:**
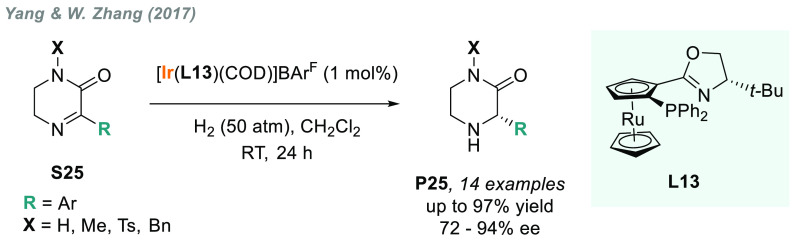
Enantioselective Synthesis of Chiral Piperazin-2-ones via AH

The AH of related nonaromatic systems such as 5,6-dihydropyrazin-2-ones **S25** was recently reported by Yang, W. Zhang, and co-workers^135^ using a phosphine-oxazoline RuPHOX ligand (**L13**). The corresponding chiral piperazin-2-ones **P25** were obtained in good yields and with moderate to good enantioselectivities (Scheme 15).

### 2.4 Cyclic *N*-Alkyl Imines

**Scheme 16:**
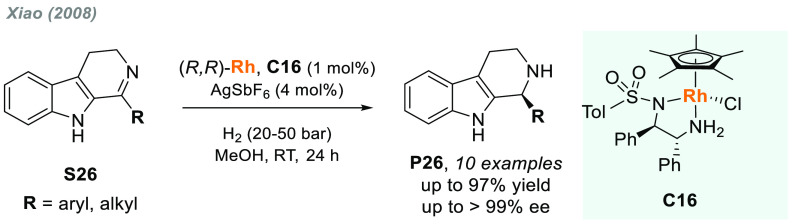
Rhodium-Catalyzed AH of Cyclic *N*-Alkyl Imines

The great progress achieved in the AH of activated and *N*-aryl imines contrasts with the often problematic AH of *N*-alkyl imines. Buchwald’s group reported the titanocene-catalyzed AH of cyclic *N*-alkyl imines back in 1994.^136^ In 2008, Xiao and co-workers identified a Rh(III)-diamine complex (**C16**) as a highly active catalyst for the AH of cyclic *N*-alkyl imines **S26** to give bioactive tetrahydro-β-carbolines^137^**P26** in optically pure form (>99% ee in the best cases, Scheme 16).^138^ Remarkably, both aryl and alkyl substituents were well-tolerated, and mostly no differences in terms of enantioselectivities were observed.

**Scheme 17:**
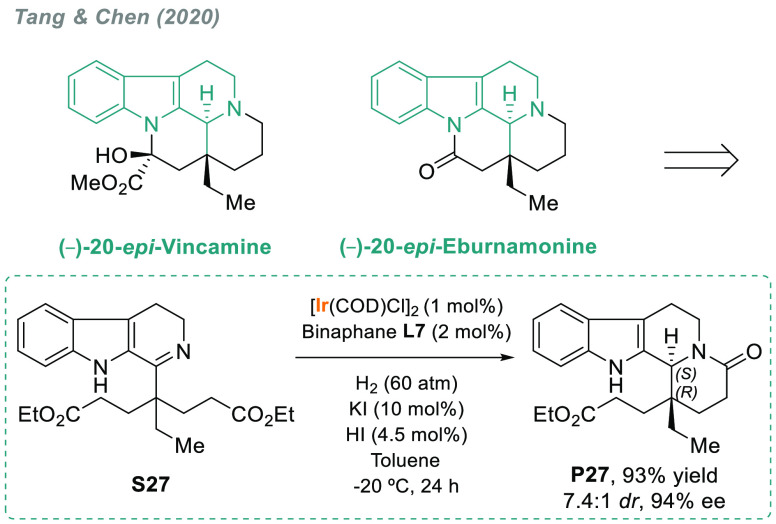
Iridium-Catalyzed Enantioselective Imine Hydrogenation/Lactamization Cascade

Dihydro-β-carbolines have been used to synthesize natural products. In 2020, Tang, Chen, and co-workers reported a concise asymmetric total synthesis of two examples of the Eburnamine–Vincamine alkaloids (Scheme 17).^139^ These syntheses featured a highly stereoselective iridium-catalyzed hydrogenation/lactamization cascade using f-binaphane **L7** as a chiral ligand, thus allowing a stereocontrolled assembly of the C20/C21 adjacent chiral centers in **P27**.

**Scheme 18:**
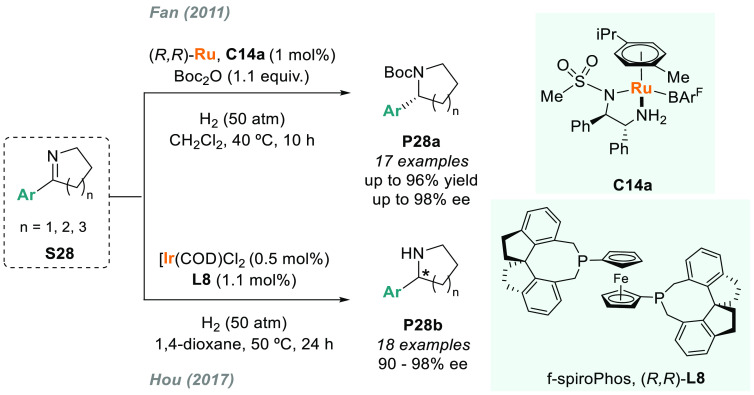
Synthesis of Chiral 2-Aryl Pyrrolidines and Piperidines via AH

Chiral cationic Ru-MsDPEN complexes have also been employed in the AH of cyclic *N*-alkyl imines. In particular, Fan disclosed that **C14a** was an efficient catalyst for the AH of **S28** to provide chiral cyclic amines **P28a** in excellent yields and enantioselectivities (Scheme 18).^140^ The same authors used a similar catalytic system for the AH of dibenzo[*c,e*]azepine derivatives to afford seven-membered cyclic amines with moderate to excellent enantioselectivities.^141^ However, in both cases, the use of Boc_2_O was required to prevent *in situ* catalyst deactivation. To circumvent this issue, Hou recently reported that the complex of iridium and (*R,R*)-f-spiroPhos **L8** as the catalyst allowed the smooth hydrogenation of a range of 2-aryl cyclic imines **S28** to **P28b** under mild conditions without any additive (Scheme 18).^142^ Hou also reported the synthesis of free cyclic amines via intramolecular reductive amination using a chiral iridium complex derived from **L8**.^143^ Previously, in 2010, X. Zhang reported an iridium-based catalytic system for the direct AH of **S28** without *in situ N*-protection, albeit with lower enantioselectivities.^144^

**Figure 3:**
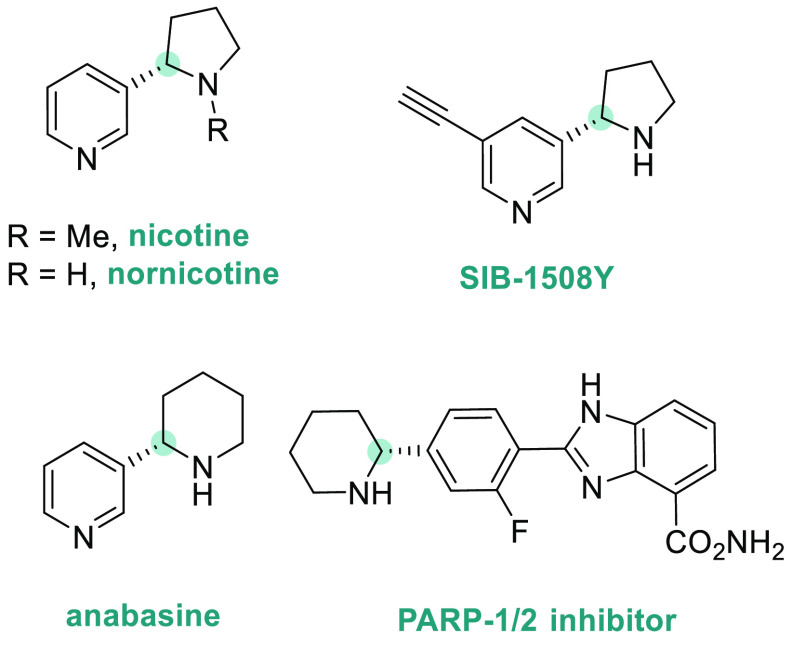
Structures of biologically active compounds and pharmaceutical drugs containing a cyclic 2-aryl amine moiety.

**Scheme 19:**
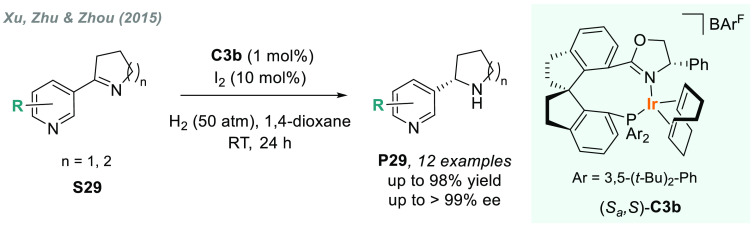
Iridium-Catalyzed AH of 2-Pyridyl Cyclic Imines

Optically active 2-aryl pyrrolidines and piperidines are an important class of structural units in many natural products and pharmaceuticals (Figure 3).^145,146^ In particular, chiral amines containing a pyridyl moiety, such as nicotine and its derivatives,^147^ are very common in alkaloid natural products and pharmaceuticals. However, the transition metal-catalyzed AH of pyridyl-containing unsaturated compounds remained a great challenge due to the strong coordinating ability of the pyridine moiety, which led to catalyst deactivation. To overcome this limitation, in 2015, Xu, Zhu, Zhou, and co-workers reported a highly efficient protocol to facilitate the exploration of nicotine-derived bioactive compounds.^148^ By using iridium catalyst **C3b** with a chiral spiro phosphine-oxazoline ligand (SIPHOX), a wide variety of chiral amines **P29** were attained in excellent yields and enantioselectivities via direct catalytic AH of 2-pyridyl cyclic imines **S29** (Scheme 19).

**Figure 4:**
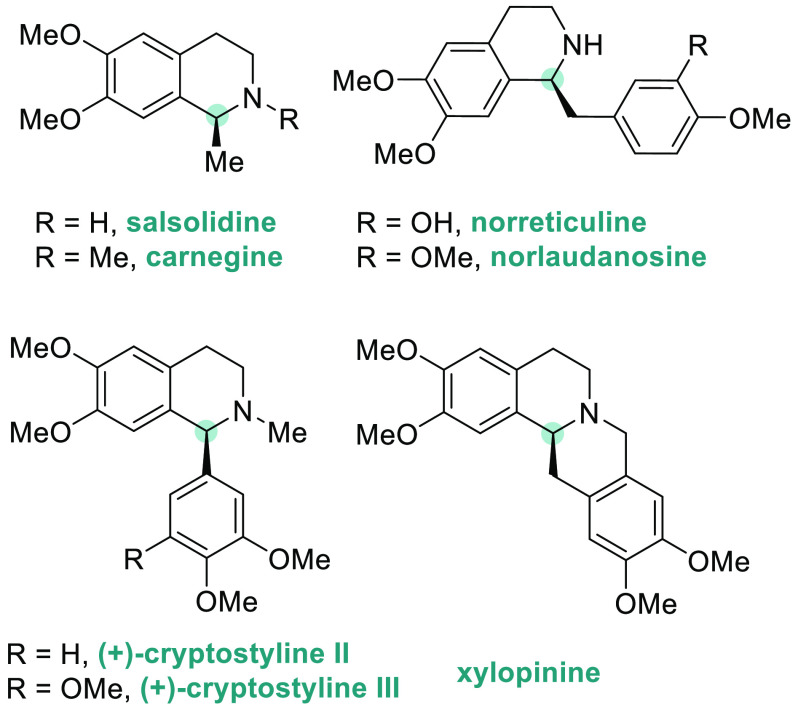
Pharmaceuticals and alkaloids containing chiral 1-substituted THIQs.

**Scheme 20:**
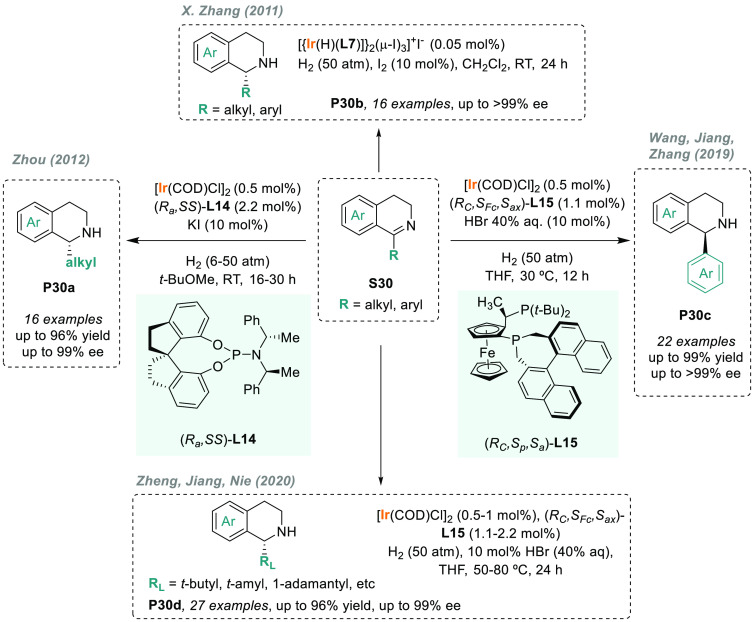
Enantioselective Synthesis of THIQs via Iridium-Catalyzed AH

Tetrahydroisoquinolines (THIQs) are an important class of alkaloids present in many pharmaceutical drugs (Figure 4).^149,150^ Therefore, the development of new enantioselective methods for their synthesis is highly desired. To this end, Zhou’s group used ligand **L14** from the family of spiro-ligands SIPHOS for the enantioselective synthesis of THIQs. In 2012, they developed a highly efficient iridium-catalyzed AH of 3,4-dihydroisoquinolines (DHIQs) **S30** with good to excellent enantioselectivities (Scheme 20).^151^ The scope of the reaction was limited to alkyl substituents.^138,152^ X. Zhang’s laboratory developed an alternative catalytic system using the iodine-bridged dimeric iridium complex with (*S,S*)-f-Binaphane **L7**.^153^ This catalyst was applied to the AH of a wide range of 3,4-dihydroisoquinolines (**S30**) including, for the first time, those bearing aryl substituents. The corresponding THIQs **P30b** were afforded with excellent enantioselectivities and high TON (Scheme 20). Unfortunately, due to steric hindrance, the enantioselectivities varied dramatically with the substrates bearing a 1-*ortho*-substituted phenyl ring. To overcome this limitation, several catalytic systems were reported as alternatives.^154−156^ Of note, Wang, Jiang, S. Zhang, and co-workers reported a direct, simple, and efficient protocol toward enantioenriched chiral 1-aryl-substituted THIQs **P30c**.^157^ For this purpose, they applied novel JosiPhos-type binaphane ligand (*t-*Bu-ax-JosiPhos) **L15** to the iridium-catalyzed AH of 1-aryl-substituted DHIQs **S30** (Scheme 20). Interestingly, the new ligand adopted the privileged properties of both JosiPhos and f-binaphane in terms of rigidity and electron-donating ability. Moreover, the use of 40% HBr (aqueous solution) as an additive dramatically improved the asymmetric induction of the catalyst. In 2020, the same catalytic system was applied to the AH of sterically hindered cyclic imines **P30d**, achieved with good to excellent enantioselectivities (74–99% ee) (Scheme 20).^158^ This novel family of chiral ligands was also applied to the iridium-catalyzed AH of acyclic *N*-aryl imines.^159^

**Scheme 21:**
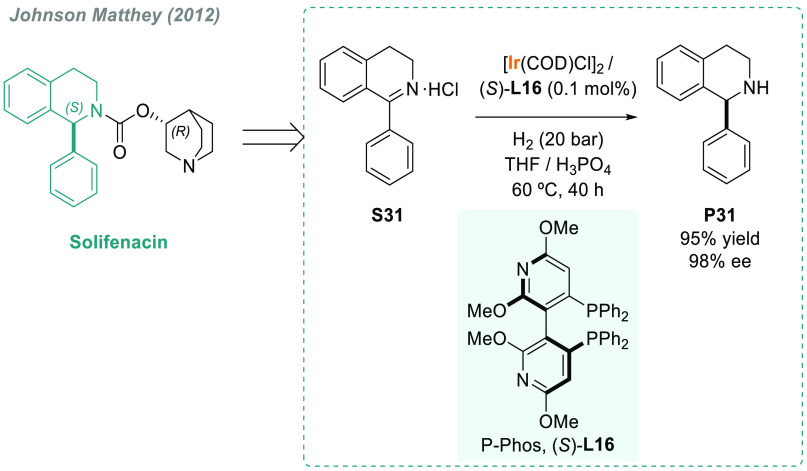
Asymmetric Synthesis of Solifenacin via Iridium-Catalyzed AH

In 2013, Zanotti-Gerosa’s group (Johnson-Matthey) described a novel approach to the synthesis of the urinary antispasmodic drug solifenacin (Scheme 21).^160^ After an exhaustive optimization process, the group demonstrated the feasibility of the process for the AH of the hydrochloride salt **S31**. The use of this salt increased reactivity in the presence of the iridium catalyst with chiral ligand (*S*)-P-Phos (**L16**). The robustness of the protocol was proved by reproducing it on 200 g scale to give **P31** in 95% yield and 98% ee.

**Scheme 22:**
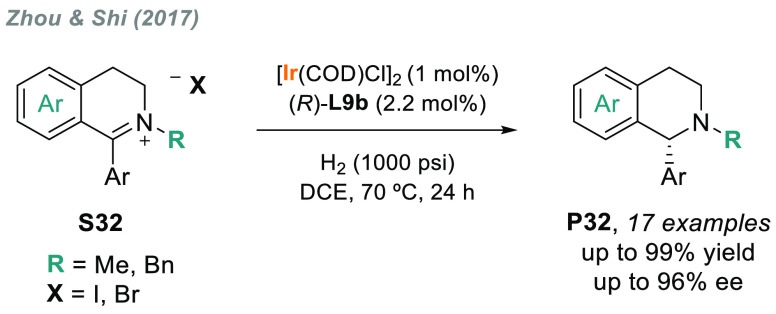
Iridium-Catalyzed AH of Cyclic Iminium Salts

The AH of iminium salts is the method of choice for obtaining tertiary amines in terms of simplicity and atom economy. In this regard, Zhou’s group described an efficient and convenient method using Ir/(*R*)-SegPhos **L9b** for the AH of cyclic iminium salts bearing a dihydroisoquinoline moiety **S32** (Scheme 22).^161^ The corresponding chiral tertiary amines **P32** were afforded in good to excellent yields and with up to 96% ee.

### 2.5 *N*-Sulfonyl Imines

#### 2.5.1 Acyclic or exocyclic *N*-sulfonyl imines

**Scheme 23:**
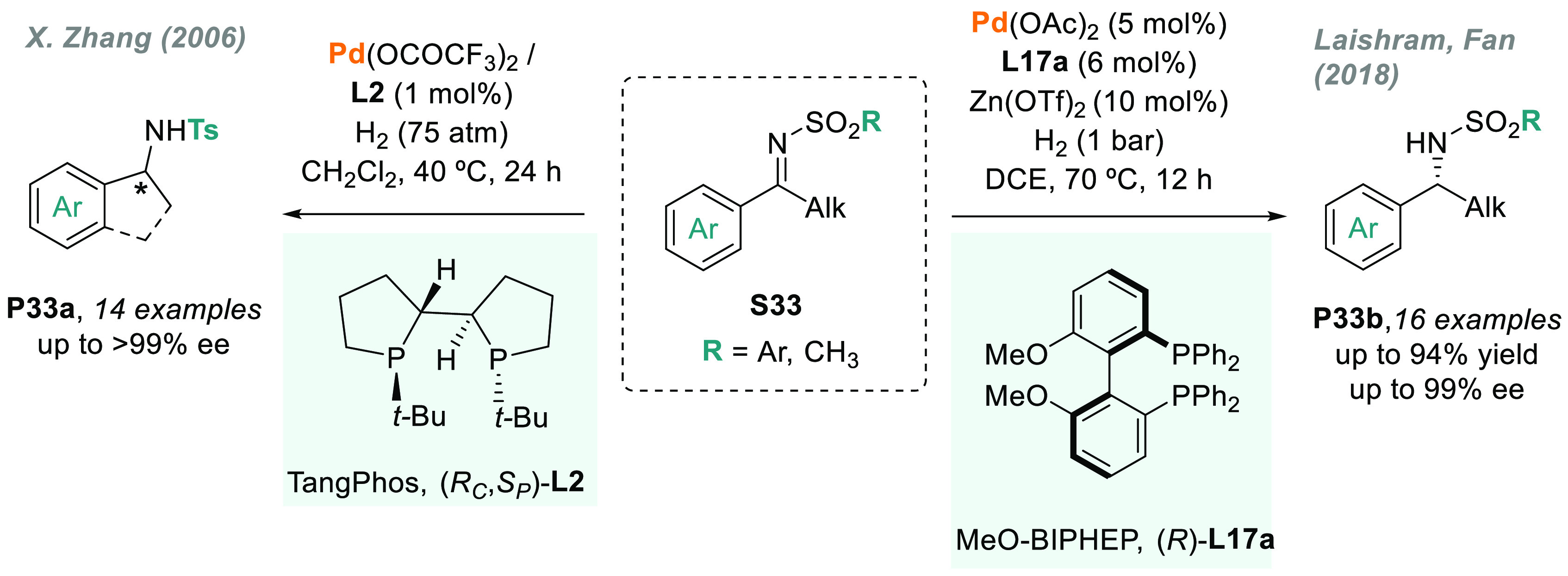
Palladium-Catalyzed AH of Aryl Alkyl *N*-Sulfonyl Imines

At the beginning of this decade, the instability of some imines prepared from ketones and the inhibitory effect of the amine products on the metal catalysts partially prevented their widespread use in AH. To overcome these limitations, *N*-sulfonyl imines, which are more stable than aryl or alkyl imines, emerged as a useful alternative. Moreover, the strong electron-withdrawing character of the sulfonyl group reduces the probability of eventual catalyst deactivation. In 2006, X. Zhang and co-workers reported an important breakthrough in the field: palladium-catalyzed AH using TangPhos (**L2**).^162^*N*-sulfonyl imines **S33** (including exocyclic imines) were efficiently hydrogenated with high levels of enantioselectivity (>99% ee in the best cases, Scheme 23). However, high H_2_ pressure was required to hydrogenate the C=N bond with full conversion. Aiming to design a catalytic system able to work at low pressure, Laishram, Fan, and co-workers recently reported a cocatalytic system based on Pd and using Zn(OTf)_2_ as an essential additive.^163^ The combination of this Lewis acid, Pd(OAc)_2_, and the axially chiral diphosphine MeO-Biphep (**L17a**) furnished the corresponding *N*-sulfonyl amines **P33b**, which show high activity and optical purity working under 1 bar of H_2_ (Scheme 23).

**Scheme 24:**
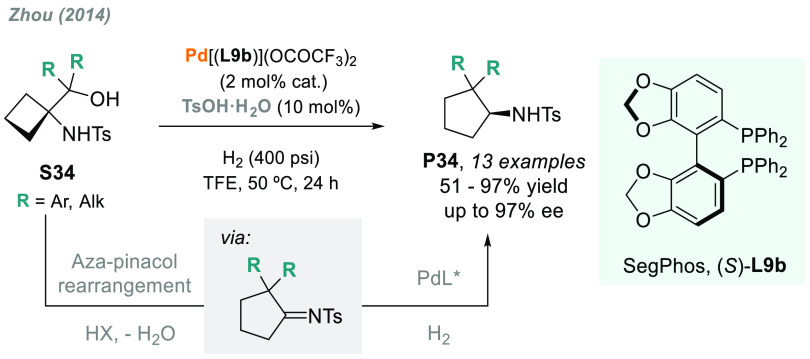
Enantioselective Palladium-Catalyzed Hydrogenation of Cyclic *N*-Sulfonyl Amino Alcohols

Palladium-based catalysts had a strong impact on AH.^164^ Furthermore, palladium-catalyzed processes involving tandem or cascade reactions are advantageous for the exploration of highly reactive intermediate species. In 2014, Zhou’s group reported an efficient palladium-catalyzed AH via hydrogenation of an intermediate generated from the acid-catalyzed aza-Pinacol rearrangement of **S34** (Scheme 24).^165^ Using the axially chiral ligand (*S*)-SegPhos **L9b**, up to 13 examples of chiral five-membered exocyclic amines **P34** were obtained in moderate to high yields and excellent enantioselectivities (up to 97% ee).

**Scheme 25:**
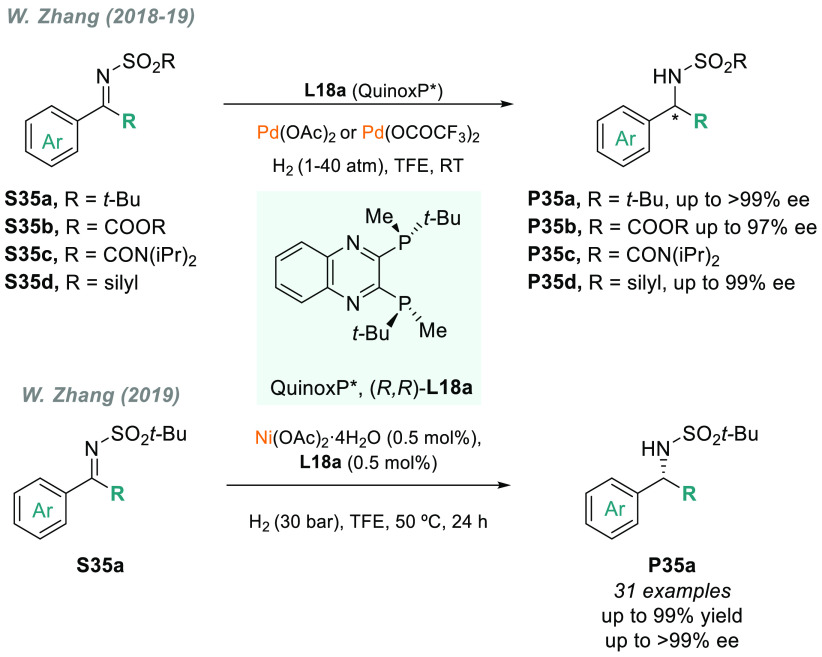
Metal-Catalyzed AH of Different Acyclic α-Substituted *N*-Sulfonyl Imines

Imines bearing small substituents, such as methyl or ethyl groups connected to the carbon atom, have been widely used as substrates. In sharp contrast, the AH of sterically demanding imines (from ketones bearing bulky substituents) or other α-heteroatom-substituted imines is still rare. W. Zhang expanded the frontiers of the AH of *N*-sulfonyl imines by employing the P-stereogenic diphosphine Quinox-P* (**L18a**),^166^ previously designed by Imamoto (Scheme 25). In 2018, the group reported the palladium-catalyzed AH of sterically hindered *N*-tosylimines under 1 bar of H_2_ pressure with high catalytic activities (S/C up to 5000) and excellent enantioselectivities (up to 99% ee, Scheme 25, **P35a**).^167^ This methodology was also applied to dialkyl *N*-tosyl imines and *N*-sulfonyl α-iminoesters^168^ with the same level of enantiocontrol. W. Zhang and co-workers also described the AH of α-iminosilanes^169^ (up to 99% ee, Scheme 25, **S35d**), albeit using higher H_2_ pressures. The low activity of earth-abundant transition metal catalysts has prevented their broad adoption in AH.^170^ Undeterred by this challenge, W. Zhang’s group recently demonstrated that the combination of nickel complexes with QuinoxP* **L18a** allows the AH of *N*-sulfonyl imines **S35**a with high catalytic activity (S/C = 10500) and exquisite enantiocontrol (>99% ee in the best cases, Scheme 25).^171^

**Scheme 26:**
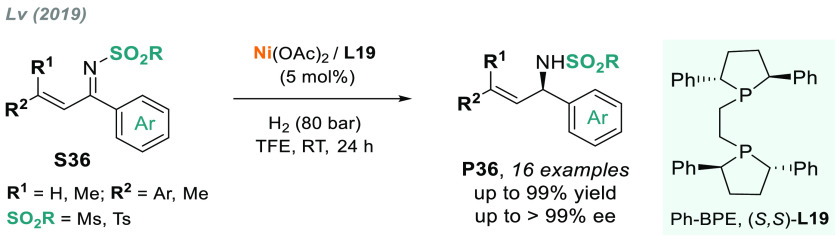
Nickel-Catalyzed Chemoselective AH of α,β-Unsaturated Ketoimines

A similar catalytic system using Ph-PBE (**L19**) as a ligand was recently reported by Lv and co-workers (Scheme 26).^172^ The nickel-catalyzed chemoselective AH of α,β-unsaturated ketoimines **S36** afforded chiral allylic amines **P36** in excellent yields and enantioselectivities. The last two examples confirm that nickel can be an effective transition metal for AH—a concept also disclosed by Chirik^173^ and Hamada.^174^

**Scheme 27:**
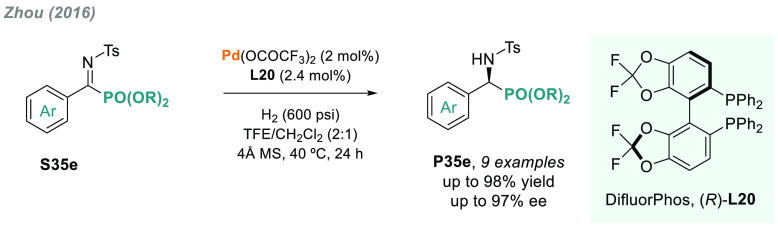
Metal-Catalyzed AH of α-Substituted *N*-Sulfonyl Imines

Other α-heteroatom *N*-sulfonyl imines have also been explored. Zhou and co-workers disclosed the palladium-catalyzed AH of a series of linear and cyclic α-iminophosphonates.^175^ The combination of Pd/(*R*)-DifluorPhos-**L20** as catalyst provided an efficient route to obtain optically active α-amino phosphonates **P35e** with up to 97% ee (Scheme 27).

#### 2.5.2 Cyclic *N*-Sulfonyl Imines

**Scheme 28:**
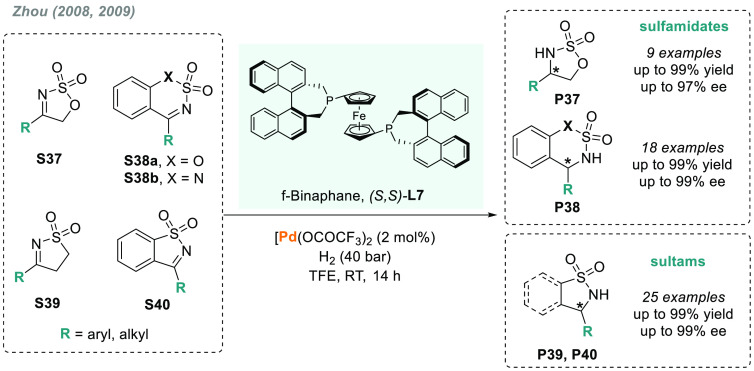
Palladium-Catalyzed AH of Sulfamidites and Sultams Using (*S,S*)-f-Binaphane as a Chiral Ligand

Sulfamidates (**P37** and **P38**) and sultams (**P39** and **P40**) are privileged building blocks in medicinal chemistry and useful chiral auxiliaries and ligands in asymmetric catalysis. They can be synthesized through the AH of the corresponding imines **S37**–**S40** (Scheme 28). Zhou and co-workers reported an efficient AH of cyclic *N*-sulfonyl imines using Pd(CF_3_CO_2_)_2_/(*S,S*)-f-binaphane-**L7** as catalyst, to afford the corresponding chiral amines in high enantioselectivity (up to 99% ee).^176,177^ The catalytic system was valid for both sulfamidates and sultams, and it was further extended to the AH of benzo-fused imines **S38** and **S40**. Fan reported a previous version of this transformation using ruthenium catalysts, but with lower enantiomeric ratios.^178^

**Scheme 29:**
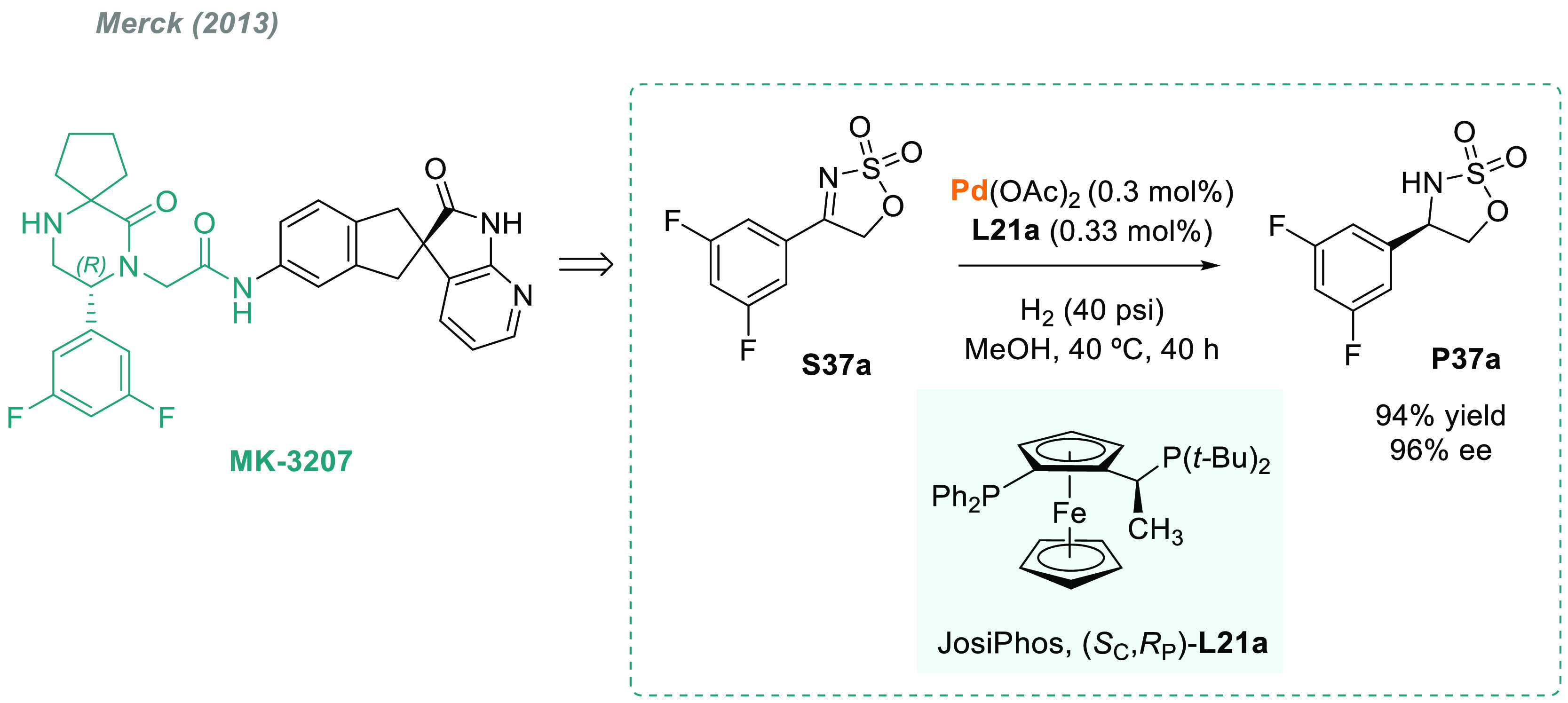
Synthesis of MK-3207 via Palladium-Catalyzed AH of Cyclic Sulfamidate Imine **S37a**

An example of the importance of chiral sulfamidates as drug building blocks is the Merck’s synthesis of MK-3207.^179^ The chirality of the benzylic stereocenter was introduced *via* the palladium-catalyzed AH of the cyclic sulfamidate imine **S37a** using either **L7** or JosiPhos (**L21a**) as chiral ligands (Scheme 29).

**Scheme 30:**
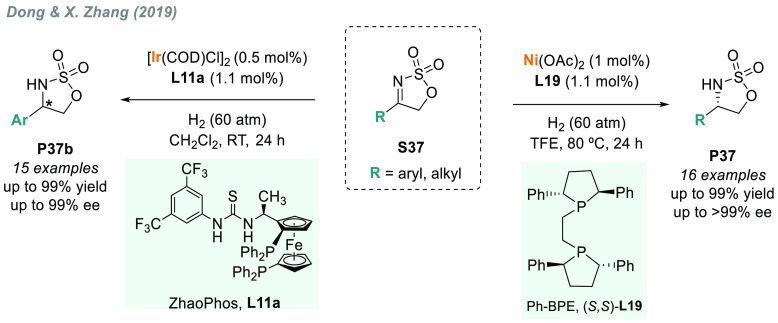
Iridium- and Nickel-Catalyzed AH of Sulfamidate Imines **S37**

More recently, in 2019, Dong, X. Zhang, and co-workers performed the iridium-catalyzed AH of **S37** using ZhaoPhos (**L11a**) to attain sulfamidates **P37b** with excellent activities and enantioselectivities (Scheme 30).^180^ ZhaoPhos is a family of chiral bifunctional diphosphine-thiourea ligands based on the synergistic cooperation between transition metal catalysis and organocatalysis.^181^ The substrate scope was limited to aryl or heteroaryl substituents. In contrast, the combination of Ni/**L19**, which gave excellent results for the AH of α,β-unsaturated ketoimines,^172^ is an alternative for the AH of **S37** bearing both aryl and alkyl substituents.^182^ Several chiral cyclic sulfamidates **P37** were prepared, even at gram scale, in high enantiomeric purity.

**Scheme 31:**
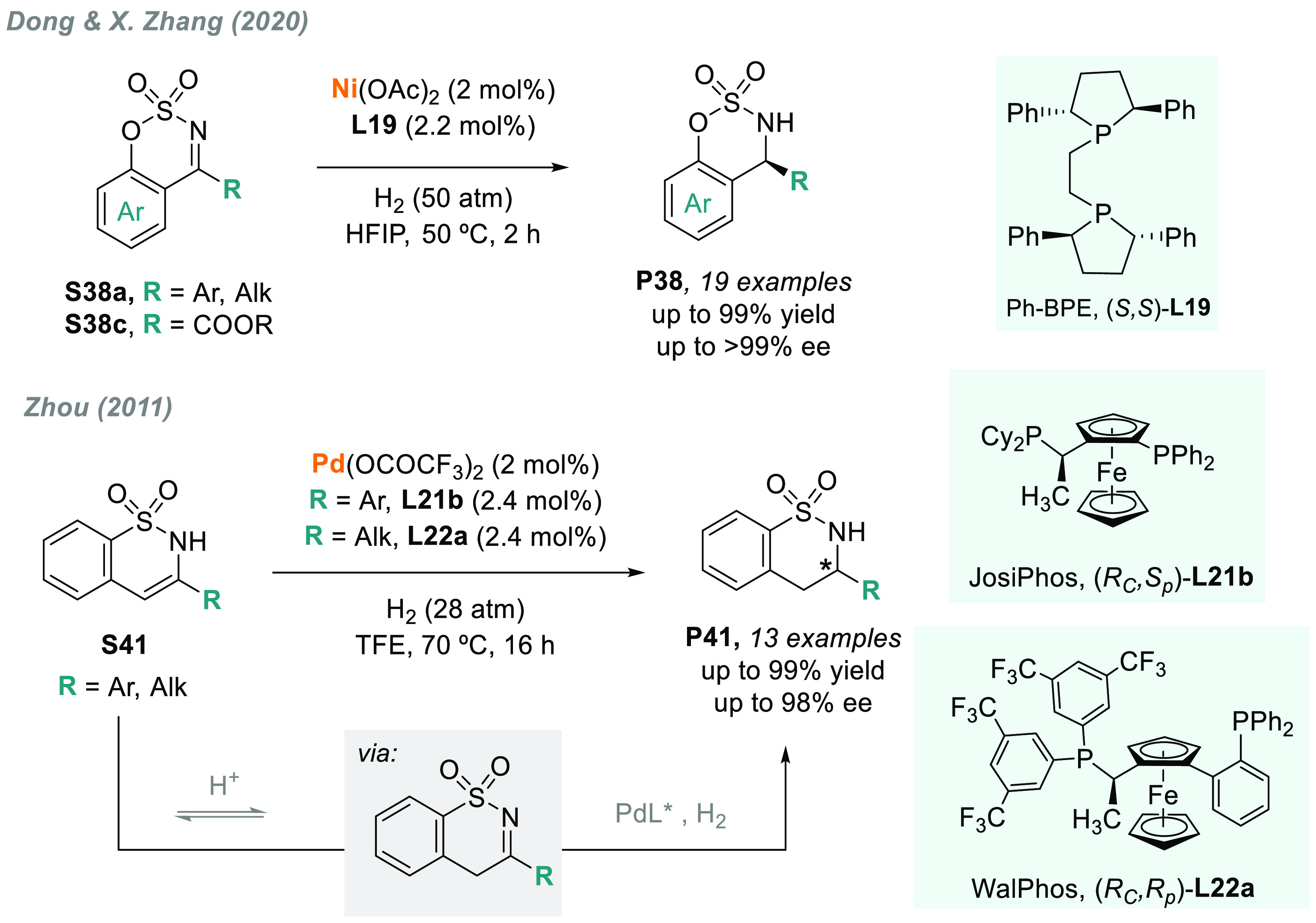
AH of Cyclic *N*-Sulfonyl Ketimino Esters **S38** and Enesulfonamides **S41**

The same catalytic system was used in the highly efficient AH of cyclic *N*-sulfonyl ketimino esters **S38c**, among other **S38a**-type substrates, which had not been disclosed before (Scheme 31).^183^ This transformation led to the facile synthesis of various chiral α-monosubstituted α-amino acid derivatives with excellent results.

Another strategy for the synthesis of cyclic sultams is the AH of enesulfonamides **S41**. In 2011, Zhou’s group reported an innovative transformation, using a catalytic system based on Pd/JosiPhos-type ligands.^184^ JosiPhos-**L21b** and WalPhos-**L22a**, in particular, were excellent chiral ligands for enesulfonamides **S41** bearing aryl and alkyl substituents, respectively (Scheme 31). Interestingly, labeling experiments confirmed that the hydrogenation was conducted via *N*-sulfonylimine intermediates. Later, in 2015, the same group reported the enantioselective synthesis of sultams by a palladium-catalyzed formal hydrogenolysis of racemic *N*-sulfonyloxaziridines with up to 99% ee.^185^

### 2.6 *N*-Phosphinyl Imines

**Scheme 32:**
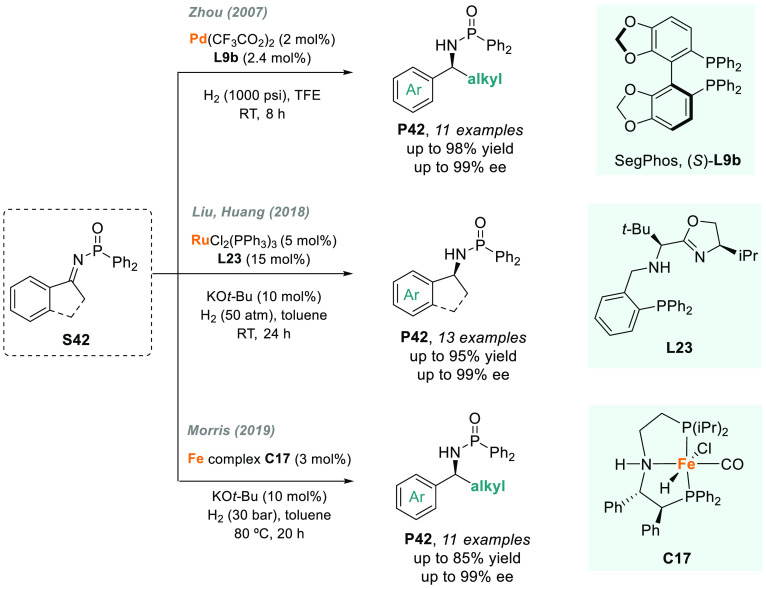
Metal-Catalyzed AH of *N*-Phosphinyl Imines

As described before, palladium complexes bearing diphosphine ligands are highly effective catalysts for the AH of *N*-sulfonyl imines in fluorinated solvents such as TFE (trifluoroethanol) or HFIP (hexafluoroisopropanol). Similarly, other activated imines, such as *N*-phosphinyl imines, are also suitable substrates for this catalytic system. After the pioneering work of Blaser,^186^ Zhou described the highly efficient palladium-catalyzed AH of activated imines, including *N*-diphenylphosphinyl ketimines.^187^ These ketimines (**S42**) were hydrogenated using **L9b** as a chiral ligand (Scheme 32), attaining excellent levels of enantioselectivity (up to 99% ee). The reaction showed a dramatic solvent effect, as only TFE led to high conversion toward **P42**.

Alternatively, Liu, Huang, and co-workers designed a phosphino-oxazoline ligand (**L23**) for the ruthenium-catalyzed AH of **S42** (Scheme 32).^188^ The catalytic system exhibited good activity and excellent enantioselectivity, providing an efficient and mild approach to optically active secondary amines **P42**. Using iron as earth-abundant transition metal, Morris and co-workers reported that an unsymmetrical iron P-NH-P′ complex (**C17**, Scheme 32) gave excellent enantioselectivity for the AH of prochiral *N*-phosphinyl imines **S42**, but with poorer activity than the previous catalytic systems.^189^ The same group had previously foreseen that these iron-hydride catalytic species were highly active toward the AH of polar bonds.^190^ Nonetheless, the system failed when using dialkyl-substituted or exocyclic *N*-phosphinyl imines, which remains as a current challenge in the field.

### 2.7 *N*-Acyl Imines

**Scheme 33:**
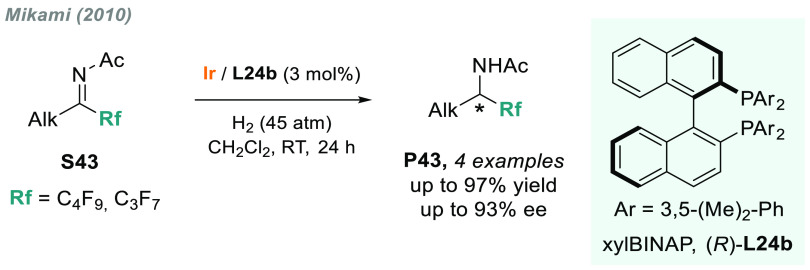
Iridium-Catalyzed AH of *N*-Acyl Imines

In 2010, Mikami and co-workers reported a catalytic AH of acyclic ketimines **S43** bearing a perfluoroalkyl chain as substituent (Scheme 33).^191^ The introduction of fluorine into molecules enhances their lipophilicity, metabolic stability, and bioavailability, thus remarkably affecting the physicochemical properties.^192−194^ Using a Ir/**L24b** (a 3,5-dimethylphenyl analog of BINAP, **L24a**) as catalytic system, four examples of chiral perfluoroalkyl amines were obtained with excellent enantioselectivity. Moreover, this work established an important precedent in the field, as the direct AH of *N*-acyl imines is still rare.^195^

**Scheme 34:**
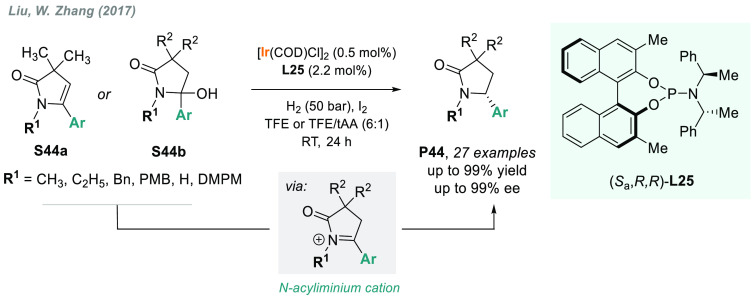
Synthesis of Chiral γ-Lactams via Iridium-Catalyzed AH of *N*-Acyliminium Cations

A novel strategy for the AH of β,γ-unsaturated γ-lactams **S44a** was described by Liu, W. Zhang, and co-workers using iridium catalysis in combination with a phosphoramidite ligand **L25** and I_2_ (Scheme 34).^196^ The chiral γ-lactams **P44** were obtained in excellent yields and enantioselectivities. Mechanistic studies detailed that the reduced products were obtained via the hydrogenation of *N*-acyliminium cations, rather than directly by the hydrogenation of **S44a**. Therefore, using the same catalytic system, these chiral γ-lactams were also prepared via *in situ* elimination/AH of racemic γ-hydroxy-γ-lactams **S44b**.^197^

**Scheme 35:**
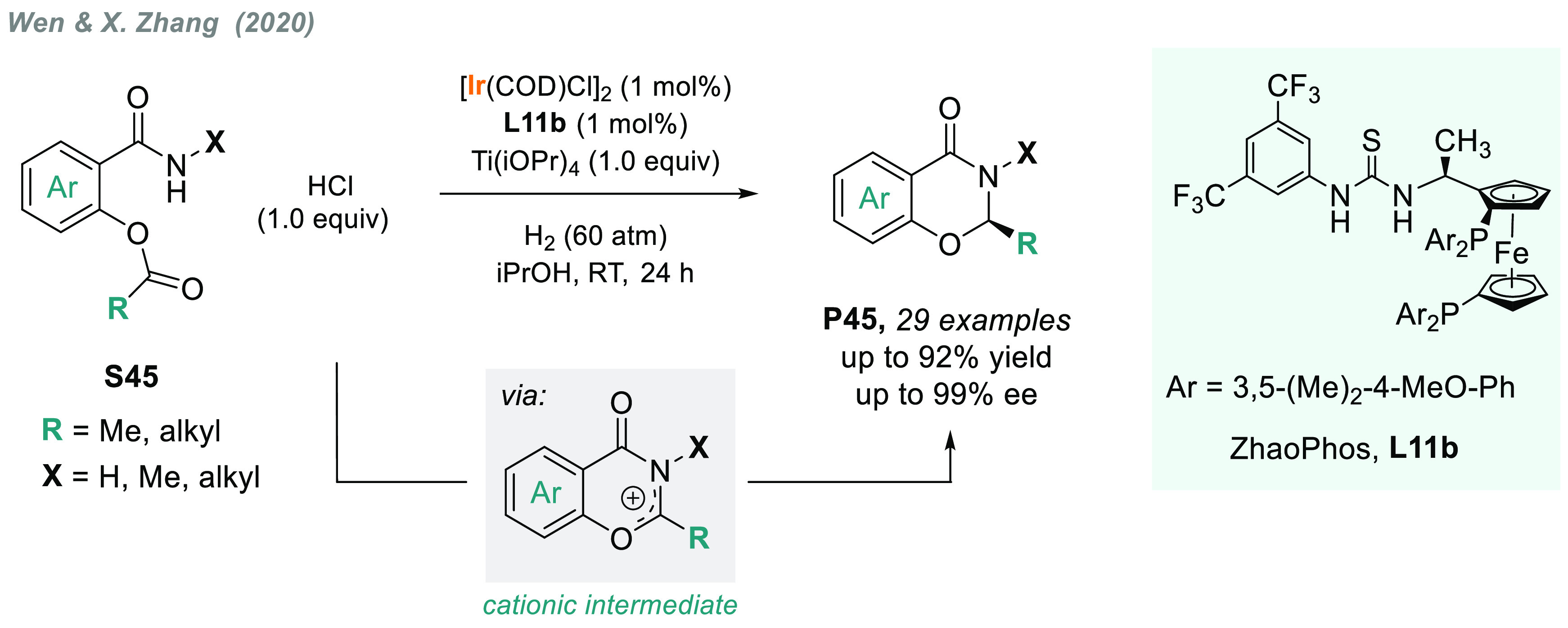
Synthesis of Chiral N,O-Acetals via Iridium-Catalyzed AH of Cationic Intermediates

A related iridium-catalyzed AH of cationic species was recently reported by Wen, X. Zhang, and co-workers for the enantioselective synthesis of chiral *N*,*O*-acetals (Scheme 35).^198^ Under acidic conditions, *O*-acetylsalicylamides **S45** underwent cyclization to generate cationic intermediates, which were subsequently hydrogenated by an iridium complex bearing a ZhaoPhos ligand (**L11b)**, thus obtaining **P45** in excellent yields and enantioselectivities.

**Scheme 36:**
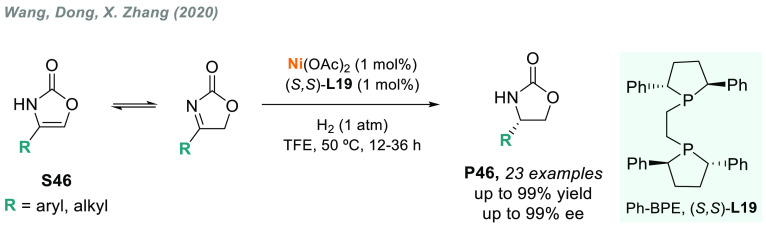
Nickel-Catalyzed AH of 2-Oxazolones

The same group recently reported the nickel-catalyzed AH of 2-oxazolones (**S46**) to afford 2-oxazolidinones in excellent yields and enantioselectivities (Scheme 36).^199^ Interestingly, deuterium labeling experiments and DFT calculations were conducted to reveal the catalytic mechanism for this hydrogenation, which indicated an equilibrium between the enamine and its imine isomer, with the latter being the substrate of choice for the asymmetric 1,2-addition of Ni(II)-H.

### 2.8 *N*-Heteroatom-Substituted Imines

**Scheme 37:**
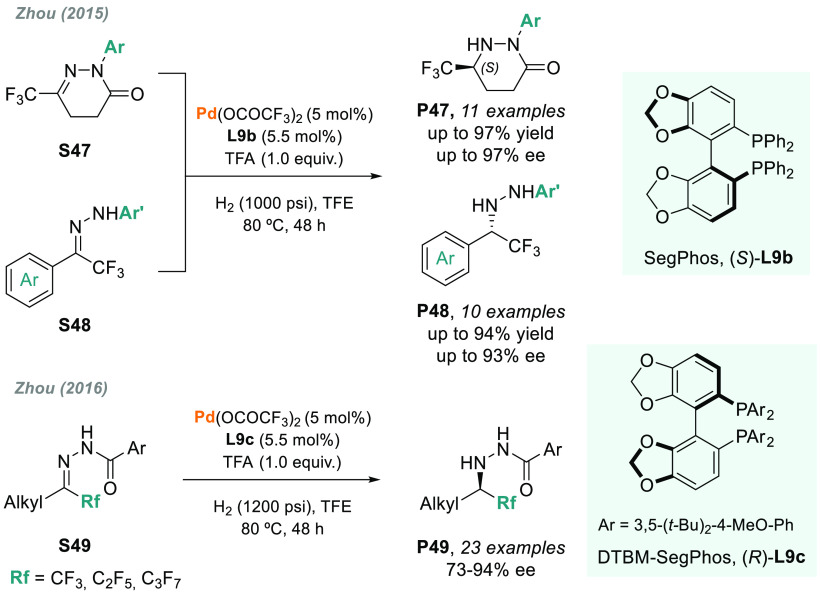
Palladium-Catalyzed AH of α-Aryl Hydrazones and α-Alkyl Hydrazones

The hydrogenation of other *N*-heteroatom imines, such as hydrazones or oximes, remains a challenge. In 2015, Zhou and co-workers reported the enantioselective synthesis of cyclic and linear chiral trifluoromethyl-substituted hydrazines via the palladium-catalyzed AH of *N*-acyl and *N*-aryl hydrazones (**S47** and **S48**, Scheme 37).^200^ Currently, many compounds bearing a hydrazine moiety, such as atazanavir or azacastanospermine, show pharmacological activity. By using Pd/(S)-SegPhos **L9b** as a catalyst and TFA as an essential additive, chiral hydrazines **P47** and **P48** were obtained in excellent yields and up to 97% ee. A year later, the same authors reported that, by using the bulkier DTBM-SegPhos (**L9c**) as a chiral ligand, the palladium-catalyzed AH of α-alkyl hydrazones **S49** proceeded smoothly, thus affording the corresponding fluorinated hydrazines **P49** in excellent enantioselectivities (Scheme 37).^201^

**Scheme 38:**
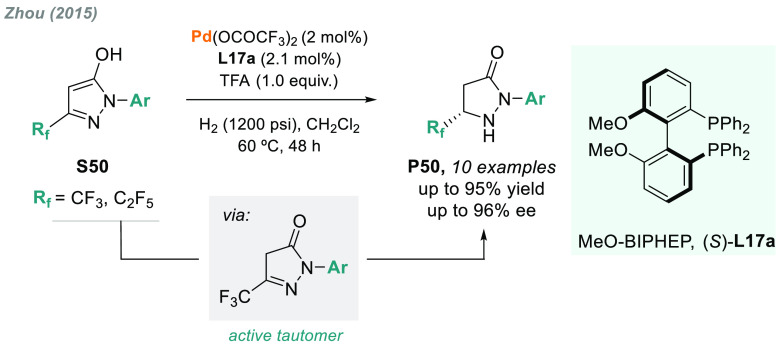
Palladium-Catalyzed AH of Fluorinated Aromatic Pyrazol-5-ols via the *CH*-Tautomer

In addition, the same laboratory reported the palladium-catalyzed AH of fluorinated aromatic pyrazol-5-ols **S50** (Scheme 38).^202^ The key for the success of this transformation is the Brønsted acid-promoted tautomerization, thus capturing the active form, followed by enantioselective hydrogenation. A wide variety of substituted pyrazolidinones **P50** were synthesized with up to 95–96% ee using (*S*)-MeO-Biphep (**L17a**) as the chiral ligand.

**Scheme 39:**
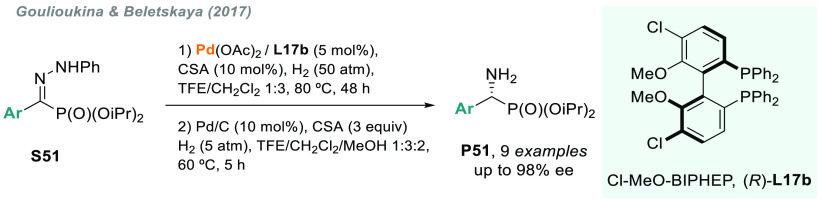
Sequential Palladium-Catalyzed AH/Hydrogenolysis of α-Hydrazono Phosphonates

In 2017, Beletskaya and co-workers reported a convenient one-pot procedure for the asymmetric synthesis of α-amino phosphonates, which are also important structural motifs in many bioactive compounds. Using a combination of Pd and biaryl chiral ligand **L17b**, the AH of α-hydrazono phosphonates **S51** proceeded with high enantiocontrol.^203^ Subsequent cleavage of the N–N bond after the addition of Pd/C and methanol into the crude reaction mixture afforded the optically active **P51** (Scheme 39).

**Scheme 40:**
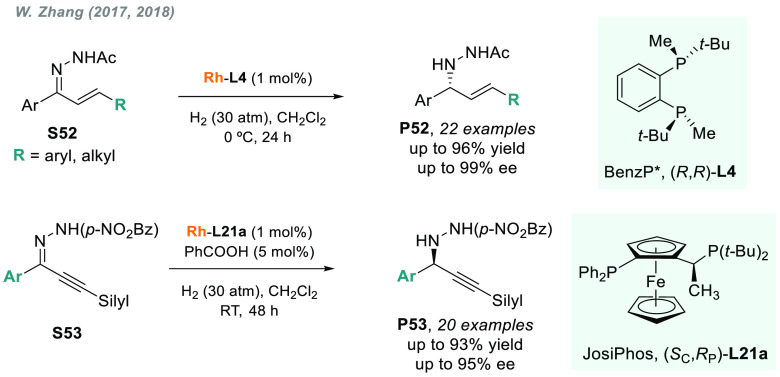
Rhodium-Catalyzed AH of Allyl and Alkynyl-aryl Hydrazones

The laboratory of W. Zhang developed highly efficient protocols for the chemo- and enantioselective hydrogenation of allyl and alkynyl hydrazones using rhodium catalysts.^204,205^ When using BenzP* (**L4**) or JosiPhos (**L21a**) as a chiral ligand, allyl or alkynyl-aryl hydrazones (**S52–S53**) were hydrogenated with excellent results (Scheme 40).

**Scheme 41:**
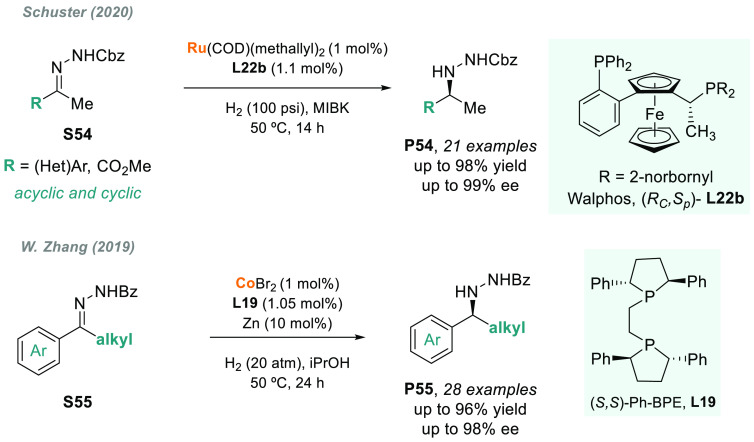
AH of Hydrazones with Ruthenium and Cobalt Complexes

Alternatively, Schuster and co-workers described the ruthenium-catalyzed AH of hydrazones **S54** using a Walphos-type ligand **L22b** (Scheme 41).^206^ The method allowed access to versatile chiral hydrazine building blocks **P54** containing aryl, heteroaryl, cycloalkyl, and ester substituents, and the protocol was demonstrated on >150 g scale. The use of Rh complexes in the AH of hydrazones had been described early this decade, but with lower ee values.^207,208^

The use of chiral Ir complexes in the AH of hydrazones was described by X. Zhang, using f-binaphane as the chiral ligand. Of note, they reported a direct catalytic asymmetric reductive amination of simple aromatic ketones with phenylhydrazide, thus offering an attractive route for the synthesis of chiral hydrazine-derived compounds.^209^

In 2019, W. Zhang and co-workers disclosed, for the first time, the efficient cobalt-catalyzed AH of C=N bonds.^210^ Although the use of cobalt as an earth-abundant transition metal in AHs was first pioneered by Chirik, the scope was limited to C=C or C=O bonds.^211−213^ Interestingly, the success of this reaction relies on the presence of an NHBz group (**S55**, Scheme 41), which acts as a directing group. The reactivity and enantioselectivity were further enhanced by assisted coordination to the cobalt atom and π–π nonbonding interactions between the phenyl groups on the substrates and the chiral diphosphine (*S,S*)-Ph-BPE **L19**. The resulting chiral nitrogen-containing compounds **P55** were attained in high yields and excellent enantioselectivities (95–98% ee).

**Scheme 42:**
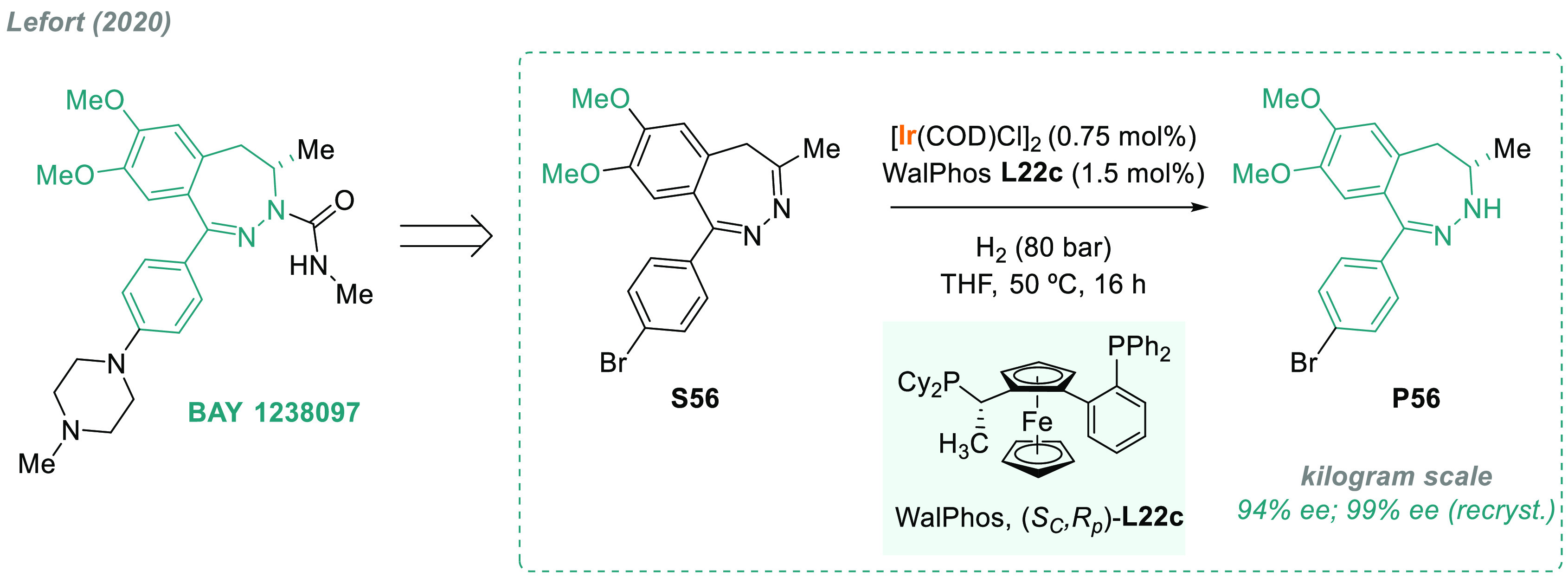
Enantioselective Synthesis of an Intermediate of BET Inhibitor BAY 1238097 via Iridium-Catalyzed AH

In 2020, Lefort and co-workers reported the first example of a regio- and enantioselective AH of a C=N–N=C motif.^214^ As shown in Scheme 42, the prochiral benzodiazepine **S56** was efficiently hydrogenated using a chiral catalyst based on Ir and a Walphos bisphosphine **L22c**. No undesired hydrogenation of the C=N double bond in the 1,2-position was observed. Using the optimal conditions, the AH was performed on a kilogram scale leading to the production of **P56**, an intermediate of BET inhibitor BAY 1238097, in enantiopure form after crystallization.

**Scheme 43:**
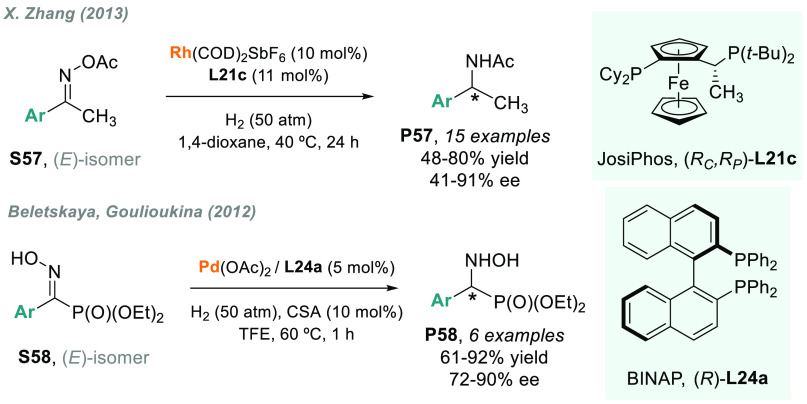
Metal-Catalyzed AH of Ketoximes

The AH of oximes and their derivatives remained a long-standing problem. To solve this gap in the field, X. Zhang and co-workers proposed the rhodium-catalyzed AH of oxime acetates **S57** (Scheme 43).^215^ Unexpectedly, the reaction led to the formation of chiral acetamide **P57** as the major product, thus affording a new strategy for the straightforward synthesis of chiral acetamides from oxime derivatives. After an exhaustive screening of phosphine ligands, JosiPhos **L21c** was found to give the highest enantioselectivity (up to 91% ee). The main limitations of this approach are the moderate activity, as well as the low enantiocontrol, in the case of *ortho*-substituted groups on the aromatic ring.

The AH of *N*-hydroxy-α-imino phosphonates **S58** was studied by Goulioukina et al.^216^ using the Pd/BINAP (**L24a**) as catalytic system, first reported by Amii and co-workers.^217^ The synthesis of chiral **P58** was achieved in up to 90% ee (Scheme 43). The catalytic reaction was performed using a Brønsted acid (CSA) as an activator and TFE as solvent. However, the scope was limited to phenyl and *para*-substituted aromatic rings.

**Scheme 44:**
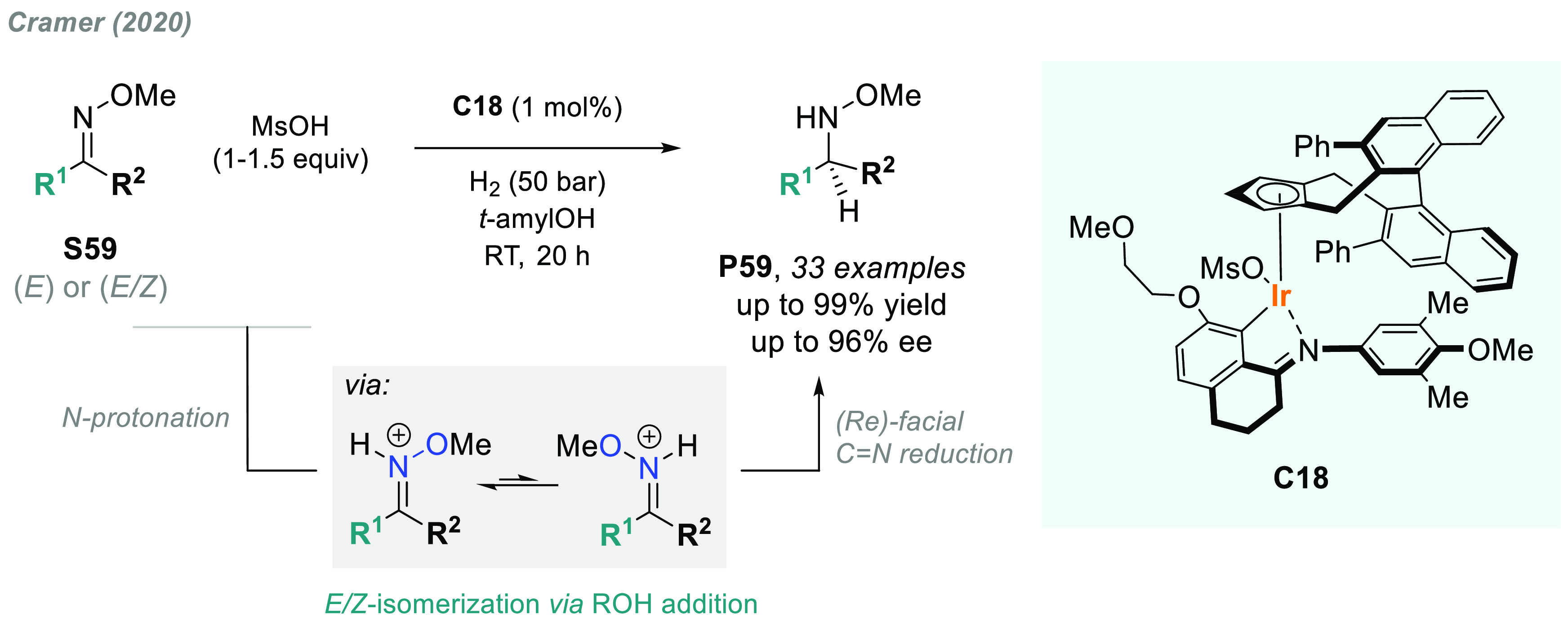
Iridium-Catalyzed Acid-Assisted AH of Oximes to Hydroxylamines

The selective reduction of an oxime to the corresponding chiral hydroxylamine derivative remains a challenge in this field because of undesired cleavage of the weak N–O bond. In this regard, in 2020, Cramer and co-workers described a methodology to overcome this limitation. He reported a robust cyclometalated Ir(III) complex **C18** bearing a chiral cyclopentadienyl ligand as an efficient catalyst for this transformation (Scheme 44).^218^ Using MsOH as activator, this acid-assisted AH of oximes **S59** avoids overreduction of the N–O bond via C=N reduction after substrate protonation, thus accessing valuable chiral *N*-alkoxy amines **P59** in excellent yields and enantioselectivities.

### 2.9 Unprotected Imines

**Scheme 45:**
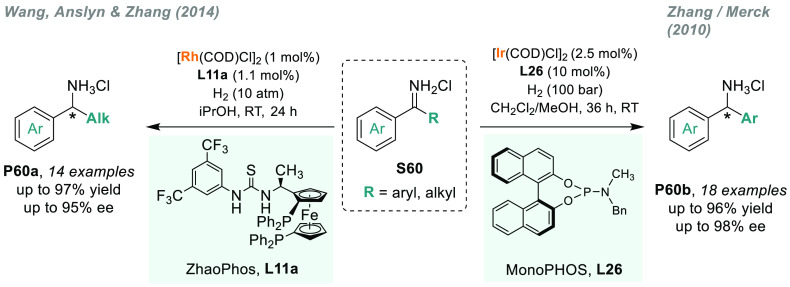
Metal-Catalyzed AH of *N*-Unprotected Imines

The transition metal-catalyzed AH of *N*-unprotected imines^64^ has been widely pursued. X. Zhang’s laboratory, in collaboration with Merck, developed the first efficient and atom-economic iridium-catalyzed AH of unprotected ketimines using HCl as Brønsted acid to activate the substrate.^219^ Ketimine hydrochlorides **S60** were efficiently hydrogenated using Ir/(*S,S*)-f-Binaphane **L7**, although in high catalyst loading (5 mol %). Later, in 2014, Wang, Anslyn, X. Zhang, and co-workers improved this transformation in terms of TON using Rh/ZhaoPhos (**L11a)** as catalyst. Taking advantage of the anion binding interaction between the thiourea and chloride counterion, chiral amines **P60a** were afforded in high yields and enantioselectivities (Scheme 45).^220^ The iridium-catalyzed AH of substituted benzophenone imines **S60** was also efficiently conducted in X. Zhang’s group.^221^ Enantioenriched diarylmethylamines **P60b** were obtained using a monodentate phosphoramidite **L26** and rather harsh reaction conditions (100 atm H_2_, Scheme 45). Substitution at the 2-position on the aryl group in **S60** is essential to achieve good enantiocontrol.

It is worth mentioning that direct asymmetric reductive amination (ARA) has become an important branch of asymmetric hydrogenation. However, as stated in the Introduction, this topic is not covered here since it has been comprehensively reviewed very recently.^10−12^

## 3 Asymmetric Hydrogenation of Enamides

In 1972, Kagan, Dang, and co-workers reported the first example of the AH of *N*-protected enamines, using the chiral ligand DIOP.^53^ Although the enantioselectivity was only moderate, this work opened a door toward the enantioselective synthesis of chiral amines. Knowles,^222^ Noyori,^223^ and Burk^224^ strongly contributed to the field by introducing DIPAMP, BINAP, and DuPhos ligands, respectively. Since then, many highly efficient Rh catalysts bearing chiral diphosphine ligands have been developed. In addition, at the beginning of the century, Reetz, Feringa, and Zhou’s groups independently demonstrated that Rh complexes bearing monodentate phosphorus chiral ligands were also highly efficient catalysts.^225−227^ The direct catalytic AH of enamides is, arguably, the method of choice for the synthesis of amino acids and chiral amines bearing a stereogenic center in the α or β position to the nitrogen atom.

### 3.1 Acyclic *N*-Acyl Enamines

#### 3.1.1 Chiral Rh Catalysts

**Figure 5:**
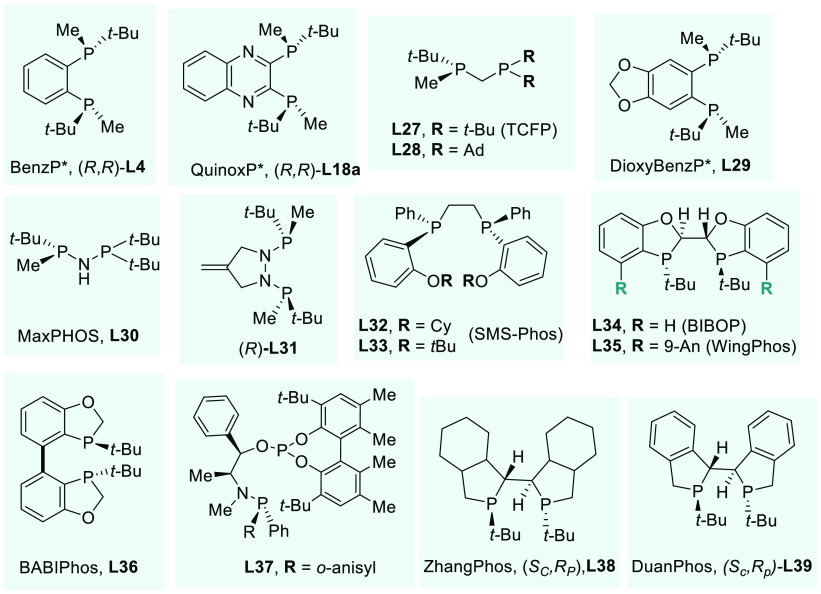
*P*-Stereogenic chiral ligands used in the metal-catalyzed AH of *N*-acyl enamines.

The presence of a coordinating group adjacent to the C=C makes *N*-acyl enamines ideal substrates for rhodium-catalyzed AH, which very often induces very high enantioselectivity.^228^ In contrast to imines, the use of iridium complexes in the AH of acyclic *N*-acyl enamines is uncommon.^229^ Nevertheless, the chiral ligand makes a critical contribution to the achievement of high activity and selectivity. Consequently, the development of more efficient ligands for a range of catalytic processes is still a vital research topic. During the past decade, new families of chiral phosphines,^230^ including monodentate phosphines,^231−234^ bis(aminophosphine)-type ligands,^235−237^ phosphino-phosphite (P-OP),^238−244^ phosphino-phosphoramidite,^245−252^ spiroketal^253,254^ or supramolecular-type^255−259^ biphosphines, and others,^260,261^ have found widespread use in the rhodium-catalyzed AH of *N*-acyl enamines.^262^ Among these, the past decade has witnessed the development of *P*-stereogenic electron-rich alkyl phosphines as highly proficient ligands.^44,45,263−265^Figure 5 shows the most relevant *P*-stereogenic ligands used in the rhodium-catalyzed AH of benchmark enamides (Table 1). These chiral ligands have stood out from others in the AH of standard *N*-acylenamines such as methyl α-acetamidoacrylate (MAA, **S61**), (*Z*)-methyl a-acetamido-3-phenyl acrylate (*Z*-MAC, **S62**), β-dehydroamino acids (**S63**),^266^ and *N*-(1-phenylvinyl)acetamide (PVA, **S64**).

**Table 1:**
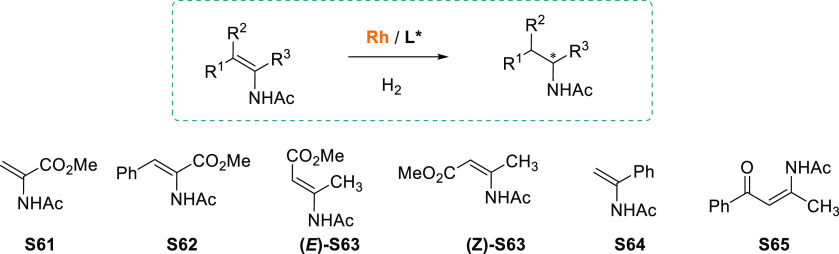
Enantiomeric Excesses (%) in the Rhodium-Catalyzed AH of Benchmark *N*-Acyl Enamines Using the *P*-Stereogenic Ligands Shown in Figure 5

	S61	S62	(*E*)- S63	(Z)-S63	S64	S65
**L27**	>99	>99	99	96	98	
**L28**	>99	>99	>99	96	97	97
**L18a**	>99	>99	>99	99	>99	97
**L4**	>99	>99	>99	98	93	99
**L29**	>99	>99	>99	99	90	99
**L30**	99	99	99	96	97	92
**L31**	98	99			97	
**L32**	>99	>99	98	88	>99	
**L33**	>99	>99	97	80	>99	
**L34**	>99	97	99	99	99	
**L36**		>99		90	96	
**L37**	99	>99			96	
**L38**	>99	>99	>99	97	>99	96
**L39**	>99	>99	>99	97	>99	96

In 2004, Hoge and co-workers established an important breakthrough in the field by preparing a *C*_1_-diphosphine with three-hindered quadrants (trichickenfootphos—TCFP, **L27**).^267−269^ This ligand showed very high enantioinduction for a wide variety of *N*-acyl enamines (**S61**–**S64**, Table 1). However, TCFP is difficult to handle in air, which explains why it has received little attention in asymmetric catalysis. To overcome this limitation, Imamoto recently prepared a crystalline, air-stable analog of TCFP by replacing the *tert*-butyl groups in the nonstereogenic phosphorus atom for the bulkier 1-adamantyl (**L28**).^270^ Its catalytic activity on the rhodium-catalyzed AH of enamides afforded excellent enantioselectivities for the substrates tested, including β-keto enamides (**S65**).^271^ In 2010, Riera and Verdaguer’s laboratory reported the first synthesis of optically pure, borane-protected primary and secondary aminophosphines.^272^ These compounds were found to be valuable *P*-stereogenic building blocks for the preparation of new chiral aminodiphosphine ligands. The synthesis and catalytic evaluation of small-bite angle MaxPHOS ligand (**L30**) was first described.^273^ Indeed, MaxPHOS is a nitrogen-containing analog of TCFP (**L27**). However, and in contrast to **L27**, the presence of an -NH- bridge between the two phosphine moieties allows the NH/PH tautomerism to take place. The protonation of MaxPHOS led to the stable PH form of the ligand, which turned into air-stable compounds both in the solid state and in solution. The complex Rh/**L30** proved to be a highly enantioselective and robust system for the AH of a wide range of *N*-acyl enamines (Table 1). Later, a new class of *P*-stereogenic *C*_2_-symmetric ligands with a hydrazine backbone was also disclosed by Riera and Verdaguer.^274^**L31**, in particular, showed excellent catalytic performance in the rhodium-catalyzed AH of several benchmark substrates (Table 1).

*C*_2_-symmetric *P*-stereogenic ligands have been widely used in AH. Stephan’s laboratory performed the rhodium-catalyzed AH of a wide spectrum of representative enamides using **L32** and **L33** (SMS-Phos) as chiral ligands.^275,276^ Both catalytic systems showed excellent enantioselectivities (>99% ee for several model substrates; Table 1). The catalytic activity of the ligand was markedly affected by the nature of its aryl substituents in terms of both bulkiness and electronic properties. Of note, *t*-Bu-SMS-Phos **L33** outperformed other reported ligands (Table 1), although the enantioselectivity dropped considerably when using tetrasubstituted vinyl acetamides.^277^

In 2010, Tang designed and synthesized a novel family of chiral bisdihydrobenzooxaphosphole ligands (BIBOP, **L34**).^278,279^ Their ease of preparation and excellent air stability make BIBOP a practical ligand. Moreover, it can also be highly modular by fine-tuning the substituents at the 4,4′-positions. The rhodium-catalyzed AH of various *N*-acyl enamines using BIBOP ligands was exploited, including in kilogram scale.^280^ When using Rh/**L34** in the AH of benchmark substrates, the corresponding chiral amines were attained in excellent enantioselectivities (Table 1). The same group later developed a similar ligand named WingPhos (**L35**), and the introduction of 9-anthracenyl substituents conferred a deeper chiral pocket.^281^ Other ligands were efficiently applied to the rhodium-catalyzed AH of (*E*)-β-aryl enamides, which is a class of substrates that remained underdeveloped.^282,283^ More recently, a novel class of benzooxaphosphole ligands (BABIPhos, **L36**) has been reported.^284^ The high catalytic performance of these ligands was showcased in rhodium-catalyzed AH, although for **S63** the enantioselectivity achieved was lower than with BIBOP.

The use of *P*-stereogenic *N*-phosphine-phosphinite ligands is still rare. Recently, Dieguez’s laboratory developed a family of these ligands (**L37**) that has been applied in rhodium-catalyzed AH.^285^ By choosing the appropriate ligand for each substrate family, benchmark enamides were hydrogenated, giving excellent results (Table 1).

Between 1998 and 1999, Imamoto pioneered the use of the *tert*-butylmethylphosphine synthon in *C*_2_ chiral diphosphines with the development of BisP* and MiniPHOS.^286−288^ Afterward, he improved the ligand design by introducing this *P*-stereogenic synthon into many other ligands such as QuinoxP* (**L18a**), BenzP* (**L4**), and DioxyBenzP* (**L29**).^289−291^ These conformationally rigid ligands are crystalline solids and, once coordinated to Rh, exhibited excellent enantioselectivities in the AH of a broad range of enamides and other functionalized alkenes (Table 1). **L18a** showed unbeatable enantioselectivities when acetamido acrylates and vinyl acetamides were used but gave poor conversion for the AH of β-keto enamide **S65**. In contrast, **L4** and **L29** gave the best results reported to date with **S65**.

**Scheme 46:**
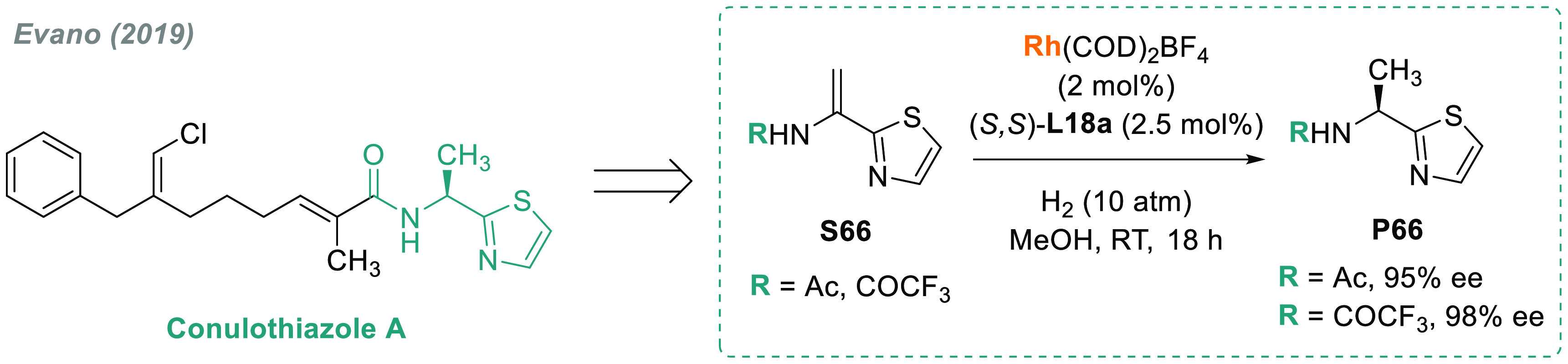
Total Synthesis of Conulothiazole A via Rhodium-Catalyzed AH

As an example of a synthetic application, Evano’s group recently developed a short and modular total synthesis of Conulothiazole A in 7 steps and 30% overall yield.^292^ One of the key steps was an efficient rhodium-catalyzed AH of a 2-enamido-thiazole **S66** (Scheme 46) using (*S,S*)-QuinoxP* **L18a**. The catalytic system was extended to a variety of 2-enamido-heteroarenes with excellent results (up to 99% ee), thus providing efficient access to 2-aminoethyl-arenes, which are useful building blocks in medicinal chemistry. Of note, the rhodium-catalyzed AH of acetamidoacrylates or vinylacetamides has been widely used as a powerful tool in total synthesis of natural products^293,294^ and for the preparation of drugs and pharmacologically active compounds.^295−302^

At the beginning of the decade, X. Zhang and co-workers reported the preparation of an electron-donating *P*-stereogenic biphospholane ligand (ZhangPhos, **L38**) for the rhodium-catalyzed AH.^303,304^ The group had also previously reported other *P*-stereogenic ligands with *C*_2_-symmetry, such as TangPhos (**L2**)^305,306^ or DuanPhos (**L39**),^307,308^ among others.^309^ Compared to those, ZhangPhos is conformationally more rigid, and it achieved better or similar enantioselectivities (up to 99% ee, Table 1). Moreover, **L38** exhibited extremely high reactivity (up to 50 000 TON) in the rhodium-catalyzed AH of a wide range of *N*-acyl enamines and had the advantage that both enantiomers can be prepared by asymmetric synthesis.

**Scheme 47:**
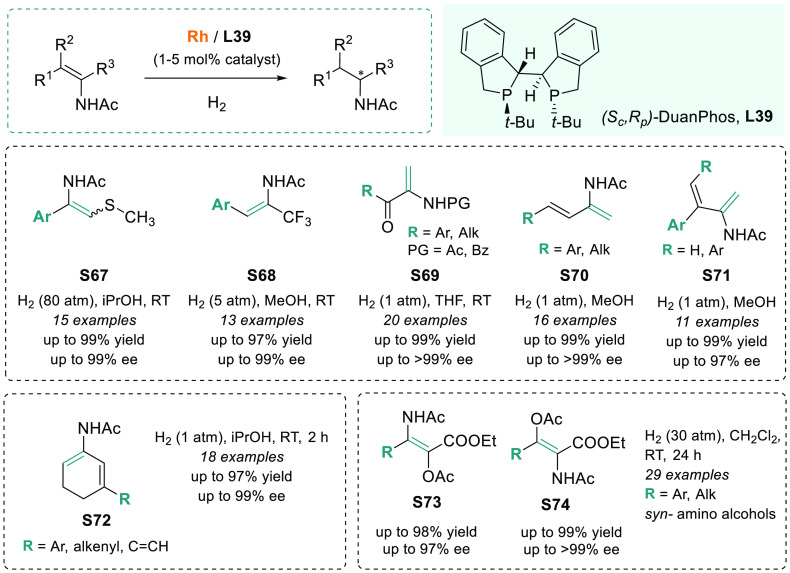
Scope of Substrates for Rhodium-Catalyzed AH Using DuanPhos

**Scheme 48:**
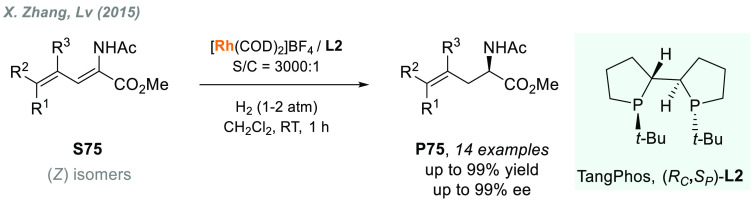
Rhodium-Catalyzed AH of Conjugated Enamides

Nevertheless, Rh-DuanPhos is a highly versatile catalytic system that was used in many other functionalized substrates. Wiest, Dong, and co-workers recently applied this chiral catalyst in the cascade hydrogenation of cyclic dehydropeptides controlled by catalyst–substrate recognition.^310^ Previously, X. Zhang, Lv, and co-workers used this catalyst for the efficient AH of β-acetylamino vinylsulfides **S67**,^311^ α-CF_3_-enamides **S68**,^312^ α-dehydroamino ketones **S69**,^313,314^ aliphatic dienamides **S70**(315) and **S71**,^316^ and cyclic dienamides **S72** (Scheme 47).^317^ The resulting chiral amines were afforded in excellent yields and enantioselectivities. Furthermore, other challenging functionalized substrates, such as tetrasubstituted enamides, were hydrogenated in a highly enantioselective manner. In particular, the AH of α-acetoxy β-enamido esters **S73**(318) and β-acetoxy α-enamido esters **S74**(319) for the preparation of *syn* amino alcohols was conducted using Rh/DuanPhos catalyst, achieving excellent results (Scheme 47). In 2015, the same group also reported the highly regio- and enantioselective synthesis of γ,δ-unsaturated amido esters **P75** by AH of conjugated enamides using Rh/TangPhos-**L2** (Scheme 48).^320^

**Scheme 49:**
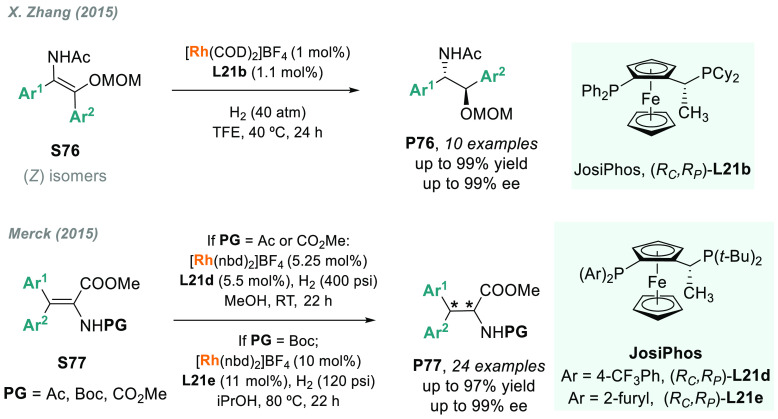
Rhodium-Catalyzed AH of (*Z*)- Tetrasubstituted Enamides

In addition, the AH of tetrasubstituted enamides in *Z* form was also accomplished by the same laboratory (Scheme 49).^321^ However, in this case, Rh-DuanPhos-**L39** gave poor conversion for **S76**. In contrast, JosiPhos ligand **L21b** afforded a set of *anti* β-amino alcohol derivatives **P76** in excellent yields and enantioselectivities. Simultaneously, scientists at Merck reported a concise, enantio- and diastereoselective route to novel nonsymmetrically substituted *N*-protected β,β-diaryl-α-amino acids and esters through the AH of tetrasubstituted enamides **S77** (Scheme 49).^322^ Again, JosiPhos ligands (**L21d** and **L21e**) allowed complete stereocontrol over the two vicinal stereogenic centers. Remarkably, an example of **S77** was previously hydrogenated by Ramsden and co-workers for the asymmetric synthesis of an intermediate of denagliptin.^323^ The rhodium-catalyzed AH of other tetrasubstituted enamides has also been investigated. A noteworthy example was the asymmetric synthesis of the cannabinoid-1 receptor inverse agonist taranabant, reported by a Merck team in 2009.^324^

**Scheme 50:**
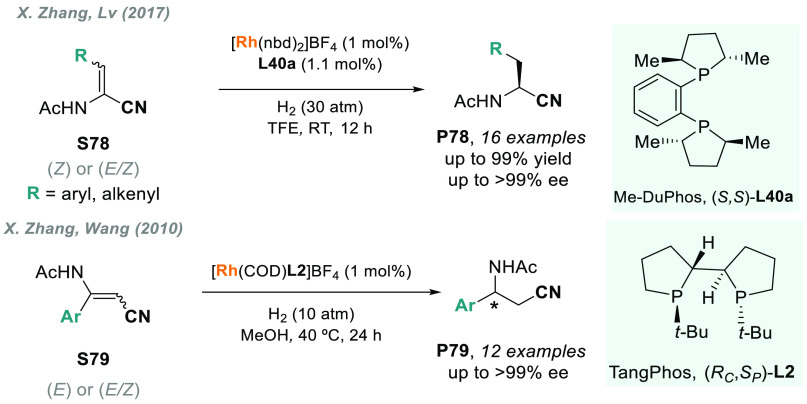
Rhodium-Catalyzed AH of α- and β-Amino Acrylonitriles

Another important transformation in this section is the rhodium-catalyzed AH of α-amino acrylonitriles **S78**, as it provides a concise route to the synthesis of chiral α-acylamino nitriles **P78** (Scheme 50). These compounds are versatile synthetic intermediates, and they can be direct precursors of valuable α-amino acids. X. Zhang and co-workers recently reported that Rh-Me-DuPhos (**L40a**) is an efficient catalyst for this transformation, thus furnishing **P78** in excellent yields and enantioselectivities.^325^ Previously, the same group described the highly enantioselective rhodium-catalyzed AH of β-acylamino acrylonitriles **S79** using TangPhos (**L2**) or QuinoxP* (**L18a**) as chiral ligands (Scheme 50).^326,327^ Interestingly, in both cases, the hydrogenation of an *E/Z* mixture gave excellent enantioselectivities, thus making it unnecessary to isolate the substrate’s isomers.

**Scheme 51:**
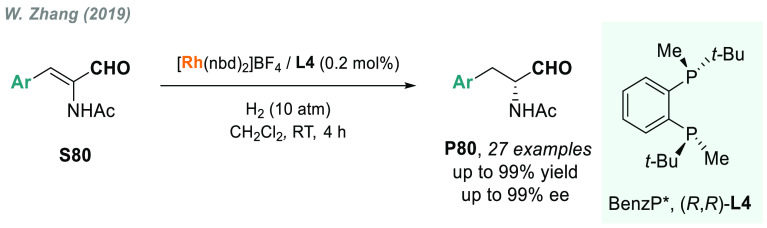
Rhodium-Catalyzed AH of α-Formyl Enamides

In 2019, W. Zhang and co-workers described a powerful strategy for the preparation of enantioenriched chiral α-amido aldehydes, which have many potential applications in organic synthesis and medicinal chemistry. Using a rhodium complex of a *P*-stereogenic biphosphine ligand ((*R,R*)-BenzP*, **L4**), α-formyl enamides **S80** were hydrogenated in a highly chemo- and enantioselective manner (up to >99.9% ee, Scheme 51).^328^ Under different hydrogen pressures, the preparation of highly enantioenriched β-amido alcohols is also plausible. The method can be carried out on a gram scale, thus demonstrating its high efficiency and practicability.

**Scheme 52:**
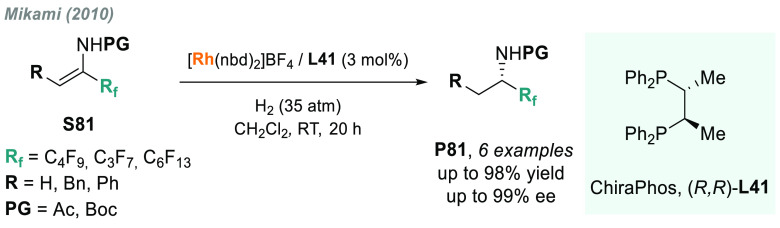
Enantioselective Synthesis of α-Perfluoroalkylated Chiral Amines

Although the AH of enamido esters, vinyl acetamides, or related compounds has received the most attention in the field, the AH of other α-and β-functionalized enamides constitutes a privileged methodology in the design of new pharmaceuticals and agrochemicals. In 2010, Mikami and co-workers described the enantioselective synthesis of α-(perfluoroalkyl)amines via the rhodium-catalyzed AH of enamides **S81**, which can be prepared by perfluoroalkylation of nitriles with Ti/Mg-reagents.^329^ By using ChiraPhos **L41**, acyclic perfluoroalkyl *sec*-amines were furnished with excellent enantioselectivities (Scheme 52). Also in 2010, Benhaim et al. reported the first enantioselective synthesis of β-trifluoromethyl α-amino acids using rhodium-catalyzed AH with TCFP (**L27**).^330^

**Scheme 53:**
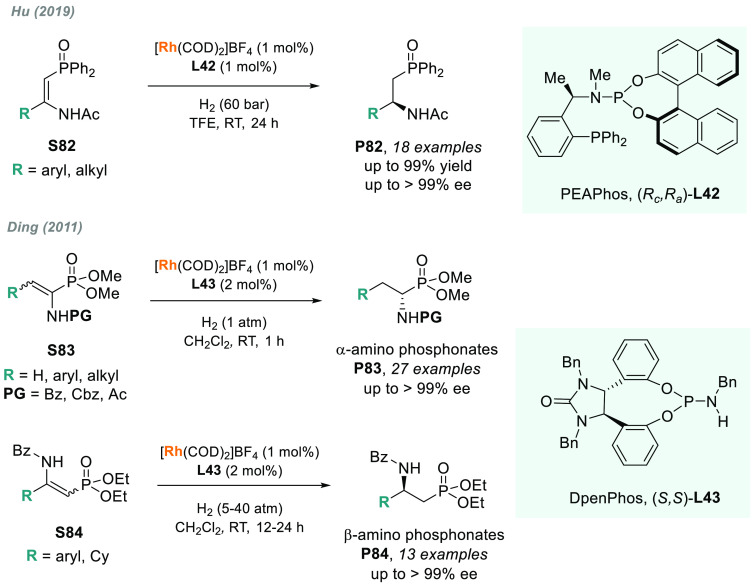
Rhodium-Catalyzed AH of Enamido Phosphonates and β-Phosphorylated Enamides

Another type of well-established chiral scaffold is β-amino phosphine derivatives. Hu and co-workers recently reported an unprecedented, catalytic AH of β-phosphorylated enamides **S82** (Scheme 53).^331^ The method used rhodium catalysis derived from an unsymmetrical hybrid chiral phosphine-phosphoramidite ligand (**L42**). A wide range of aromatic and alkylic enantioenriched β-acetamidophosphine oxides **P82** were efficiently prepared. These compounds could be readily hydrolyzed and reduced, thus providing an efficient route to important chiral β-aminophosphines.

Optically active α- and β-amino phosphonic acid derivatives can also be prepared by means of AH. In fact, in 2011, Ding designed a family of chiral monodentate phosphoramidite (DpenPhos) ligands that were found to be highly efficient in the rhodium-catalyzed AH of enamides **S83** and **S84** (Scheme 53).^332^ Of note, when **L43** was used, a set of chiral amino phosphonates **P83** and **P84** were prepared with excellent results. In several cases, the enantioselectivity values obtained were higher than those reported previously.^333,334^

**Scheme 54:**
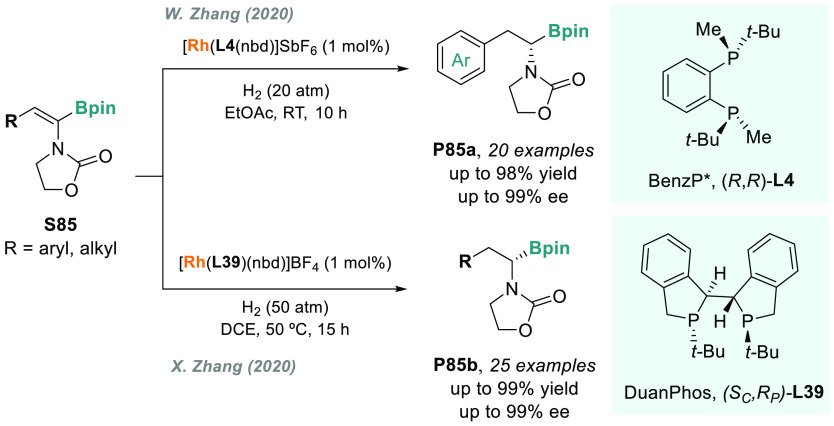
Rhodium-Catalyzed AH of α-Boryl Enamides

Organoboron compounds are also important due to their unique physical, chemical, and biological properties. However, the preparation of chiral α-aminoboronic acids, as mimics of chiral amino acids, is not trivial. In 2020, W. Zhang and X. Zhang independently pioneered this field describing the rhodium-catalyzed AH of α-boryl enamides (**S85**) using the *P*-stereogenic diphosphines **L4** and **L39**, respectively (Scheme 54).^335,336^ Critical to the success of this method was the chelate coordination of the amido group to rhodium and the nonbonding interactions between the substrate and the ligand. Whereas by using **L4** the method was limited to aryl substituents in the β position, the use of **L39** allowed an expanded substrate scope, as alkyl substituents were also well tolerated. Chiral α-amidoboronic esters **P85** were furnished in quantitative conversion and excellent enantioselectivity. Exquisite chemoselectivity was observed as no protodeboronation was detected.

**Scheme 55:**
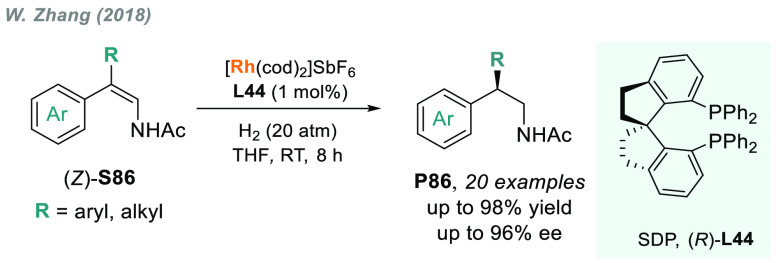
Enantioselective Synthesis of β-Stereogenic Amines via AH

While the hydrogenative synthesis of chiral α-substituted amines has been widely addressed, synthetic methodologies for the preparation of β-chiral amines are rare. Only a few examples have been reported, mostly by AH of dehydroamino acids.^337,338^ The AH of β-branched simple enamines remained a long-standing challenge due to the difficulties related to the stereocontrol of the reaction. To overcome this issue, in 2018, W. Zhang and co-workers disclosed the first catalytic protocol using a Rh complex bearing a diphosphine ligand with a large bite angle (SDP, **L44**).^339^ β-Branched simple enamides with a (*Z*)-configuration (**S86**) were efficiently hydrogenated to optically pure β-chiral amines **P86** in quantitative yields and with excellent enantioselectivities (Scheme 55).

#### 3.1.2 Ni and Co Catalysts

**Scheme 56:**
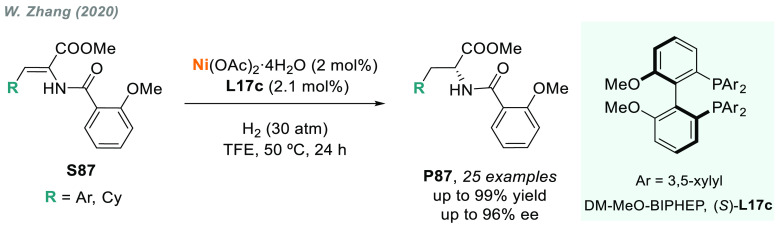
Nickel-Catalyzed AH of 2-Amidoacrylates

The limited availability, high cost, and toxicity of noble metals stimulated the research in their replacement with earth-abundant, inexpensive first-row transition metals. However, challenges such as different reaction mechanism and unexpected deactivation of the catalyst prevented their widespread use in asymmetric hydrogenation.^340,341^ While dozens of examples using Rh catalysis have been reported during the past decade, the use of earth-abundant transition metals has just started showing practical efficiency in AH. In 2020, W. Zhang and co-workers reported a highly efficient nickel-catalyzed AH of 2-amidoacrylates (Scheme 56).^342^ In contrast to the AH with Rh catalysts, where the amido-assisted activation strategy allowed attainment of high activity and enantioselectivity, Ni catalysts cannot utilize this approach as they have their own coordination modes. However, W. Zhang envisioned that other interactions between the substrate and catalyst would lead to high catalytic activity. Interestingly, when using **S87** bearing an *ortho*-methoxy-substituted benzoyl group and Ni/BIPHEP-type ligand (**L17c**), the AH occurred smoothly and the corresponding chiral α-amino acid esters **P87** were afforded in excellent enantioselectivities (up to 96% ee).

**Scheme 57:**
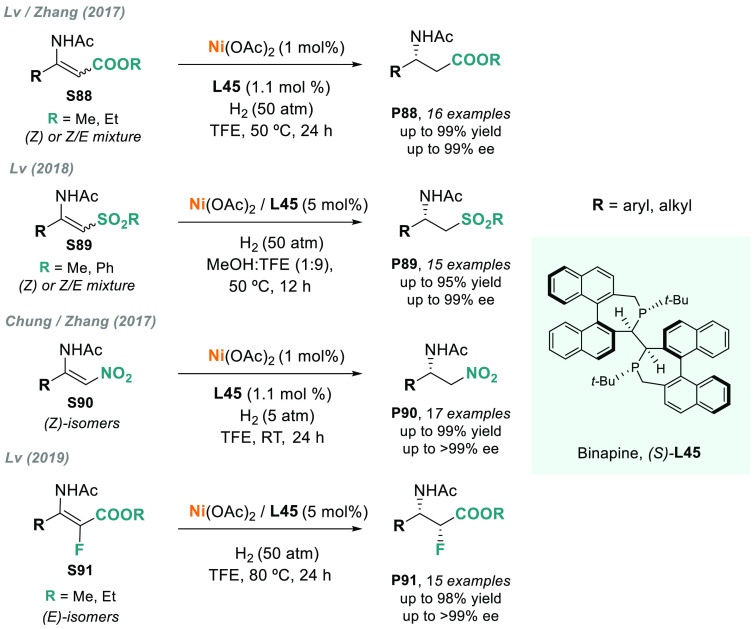
AH of β-Functionalized *N*-Acyl Enamines Using the Ni/Binapine System

Nickel-catalyzed AH has also been used in the synthesis of chiral β-amino acid derivatives. Lv and X. Zhang and co-workers reported a highly enantioselective hydrogenation of (*Z*)-β-(acylamino)acrylates **S88** to provide enantiomerically pure β-amino acid derivatives **P88** using a commercially available binapine ligand (**L45**) (Scheme 57).^343^ High enantioselectivities were obtained even using *Z/E* isomeric mixtures. The same catalytic system proved to be fruitful for many other functionalized enamides, including benchmark substrates.^344^ In 2018, Lv and co-workers expanded its use for the Ni-catalyzed AH of β-acetylamino vinylsuflones **S89** (Scheme 57).^345^ The methodology showed good compatibility with substituted (*Z*)-isomers and *Z/E* isomeric mixtures, thus being an alternative to the previously reported protocol using Rh/TangPHOS-**L2**.^346^ The resulting chiral sulfones **P89** were obtained in high yields and excellent enantioselectivities, in gram scale in the presence of only 0.2 mol % of catalyst. This catalyst also showed high activity toward the AH of β-acylamino nitroolefins **S90**. These are usually challenging substrates for AH due to the weak binding affinity of the olefins with the electron-withdrawing nitro group, and in fact, only a few examples have been reported involving precious transition metal catalysts.^347−349^ Despite this, Chung, X. Zhang, and co-workers showed that Ni/Binapine could be used as catalyst to attain chiral β-amino nitroalkanes **P90** with excellent enantioselectivity (>99% ee in the best cases) and high TONs using mild conditions (Scheme 57).^350^ Finally, Lv also reported the AH of tetrasubstituted β-enamino-α-fluoro esters **S91** in high yields and excellent diastereo- and enantioselectivities using Ni/**L45** (Scheme 57).^351^ Interestingly, key experiments revealed the critical role of acidic solvent in modulating the reaction pathway, as well as for the control of diastereoselectivity. This method provides a highly straightforward and concise route to α-fluoro-β-amino esters **P91**.^352^

**Scheme 58:**
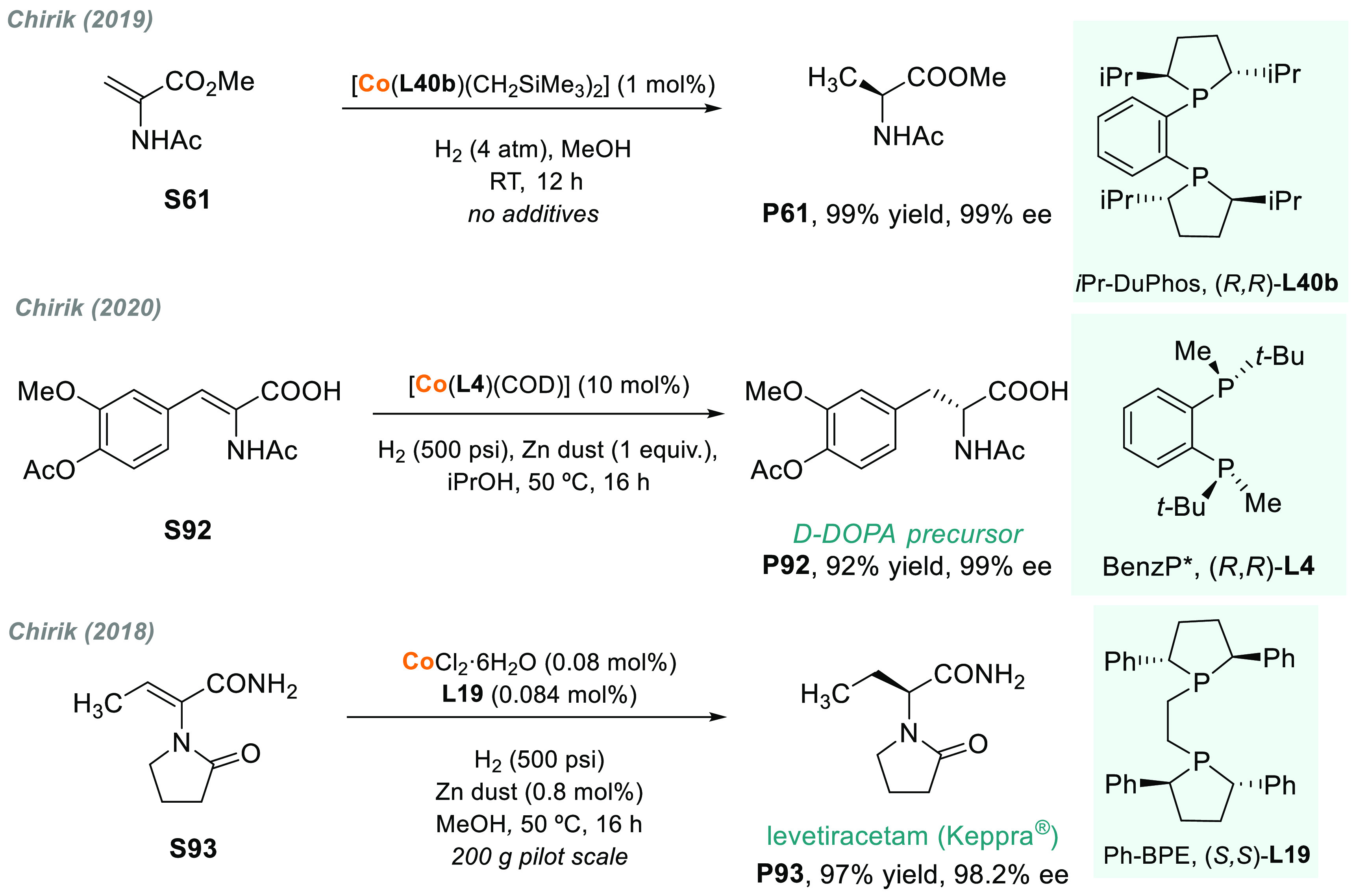
Co-Catalyzed AH of 2-Functionalized *N*-Acyl Enamines

Cobalt has also gained great importance during the past decade in the field of AH. Chirik’s laboratory has pioneered the use of cobalt complexes bearing chiral diphosphines to attain hydrogenative processes with extraordinary activity and enantioselectivity.^353,354^ In 2019, the group demonstrated that cobalt complexes bearing DuPhos-type ligand (**L40b**) efficiently hydrogenated MAA **S61** in excellent enantioselectivity (Scheme 58).^355^ More importantly, the reaction was carried out using MeOH, an industrially preferred green solvent which is often a poison for reduced earth-abundant metals, and without the use of additives. Other α,β-unsaturated carboxylic acids, including di-, tri-, and tetra-substituted acrylic acid derivatives, as well as dehydro-α-amino acid derivatives, were hydrogenated using Co/BenzP*-**L4** (Scheme 58).^356^ Chiral carboxylic acids, including bioactive ones such as Naproxen, (*S*)-Flurbiprofen, and a D-DOPA precursor **P92**, were attained in high yields and enantioselectivities. Again, protic solvents such as MeOH were identified as optimal, and Zn dust was used stoichiometrically. The group had previously described the Co-catalyzed AH of enamides using zinc-activation, which promoted straightfroward single-electron reduction to enable the catalytic process (Scheme 58).^357^ The optimized protocol, using Co/**L19**, exhibited high activity and enantioselectivity and allowed the asymmetric synthesis of the epilepsy drug levetiracetam (**P93**) at 200-g scale with only 0.08 mol % of catalyst loading.

### 3.2 Endocyclic *N*-Acyl Enamides

**Scheme 59:**
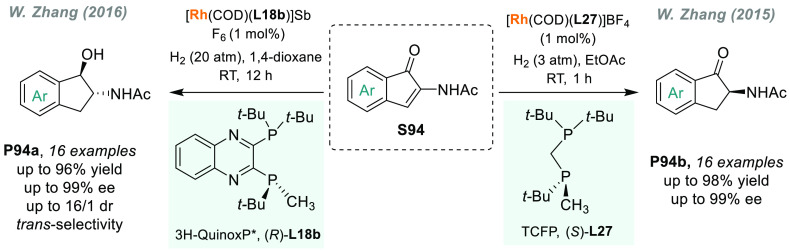
Partial and Total Rhodium-Catalyzed AH of Cyclic α-Dehydroamino Ketones

In contrast to acyclic enamides, which have been extensively studied, the AH of cyclic enamides remained a challenge before the past decade. Despite that, the resulting chiral cyclic amines are very useful structural motifs that can be found in a range of bioactive molecules.^358^ An example of this class of substrates are cyclic α-dehydro amino ketones (**S94**, Scheme 59). In 2016, W. Zhang and co-workers reported that *P*-stereogenic chiral ligand **L18b**, using Rh catalysis, efficiently hydrogenated **S94** to chiral cyclic *trans*-β-amino alcohols **P94a** via a one-pot sequential AH with excellent enantioselectivities and diastereoselectivities.^359^ The same group achieved rhodium-catalyzed partial hydrogenation using small-bite angle ligand TFCP (**L27**) in a completely chemoselective manner (Scheme 59).^360^ Thus, chiral α-amino ketones **P94b** were exclusively obtained with excellent results, and both synthetic protocols were scaled up to gram scale. In contrast, the AH of cyclic β-keto enamides remains unexplored, with only one precedent in the literature and with very limited scope.^361^

**Scheme 60:**
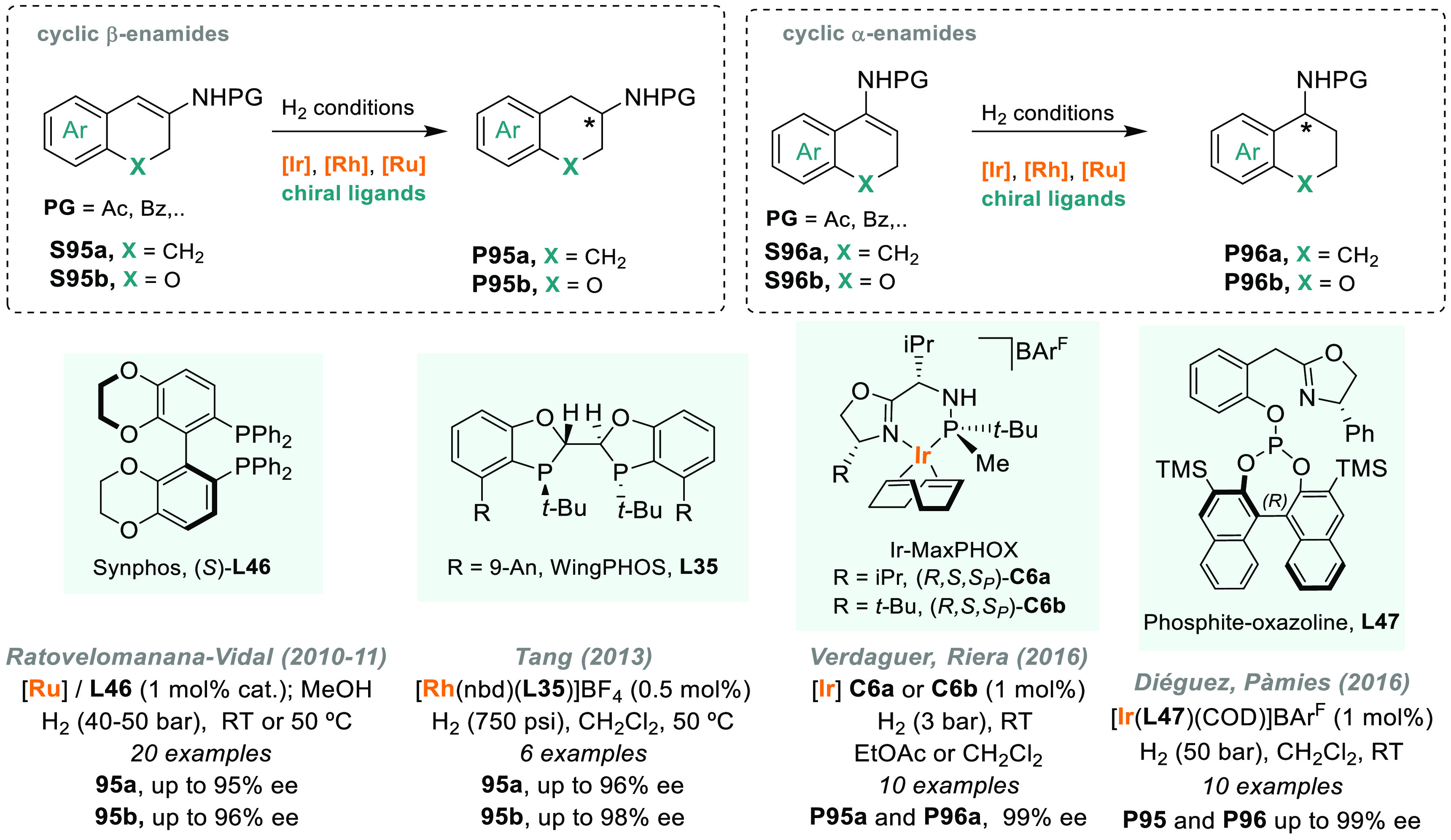
Metal-Catalyzed AH of Cyclic Enamides Derived from α- and β-Tetralones

Another family of long-standing challenging substrates are cyclic enamides derived from tetralones and chromanones. The resulting chiral amines are highly desirable as they are precursors of therapeutic drugs. In this regard, the AH of cyclic enamides has typically relied on the use of Rh and Ru catalysts.^362−365^ Among the most successful examples, Ratovelomanana-Vidal and co-workers reported up to 96% ee in the reduction of **S95** (Scheme 60). The method employed Ru catalysis in combination with binap-type ligand SynPhos **L46**.^366,367^ Later, Tang and co-workers described the use of WingPHOS ligand (**L35**) in the rhodium-catalyzed AH of cylic enamides **S95**, which yielded the corresponding chiral amines **P95** in up to 98% ee (Scheme 60).^281^

**Figure 6:**
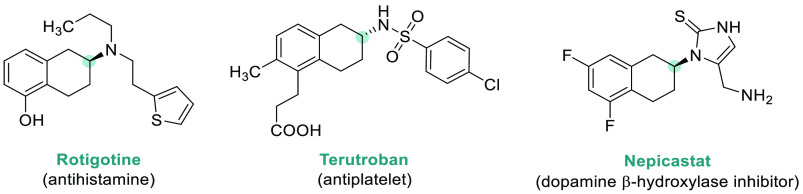
Pharmaceutical drugs containing the chiral 2-aminotetraline structure.

However, these methods suffer from harsh reactions conditions such as high H_2_ pressure or heating. In this regard, iridium catalysis, which has scarcely been used in the AH of *N*-acyl enamides and other alkenes bearing a metal-coordinating group, offered an excellent alternative. In 2016, Verdaguer, Riera, and co-workers reported the highly enantioselective iridium-catalyzed AH of cyclic enamides **S95** and **S96**, derived from α- and β-tetralones (Scheme 60).^77^ They optimized the iridium complexes bearing *P*-stereogenic phosphino-oxazoline ligands (**C6a** or **C6b)**. These catalytic systems provided the highest selectivity reported to date for the reduction of these substrates. The resulting chiral amines **P95** and **P96** were obtained in 99% ee. Moreover, the process was carried out in environmentally friendly solvents such as MeOH and EtOAc without loss of selectivity and under very mild conditions (3 bar of H_2_). When the ligand was replaced with a *P*-stereogenic phosphino-imidazole ligand, the enantioselectivity decreased considerably.^368^ Diéguez and co-workers also reported the iridium-catalyzed AH of cyclic enamides **S95** and **S96** in excellent enantioselectivities employing a phosphite-oxazoline ligand (**L47**, Scheme 60).^369,370^ The same group further extended this methodology using other modular ligands.^371−374^ Overall, these protocols allowed an efficient route to the asymmetric synthesis of 2-aminotetralines and 3-aminochromanes, key structural units in many biologically active agents such as rotigotine, terutroban, and nepicastat (Figure 6).^375,376^

**Figure 7:**
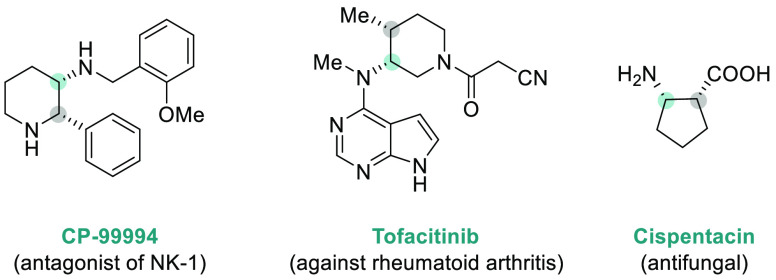
Pharmaceutical drugs containing amines with vicinal chiral centers.

The AH of tetrasubstituted endocyclic enamides^29^ has been a focus of great attention over the last years. The resulting chiral cyclic amines with a substitution at the 2-position are important motifs in many bioactive molecules and drugs (Figure 7).

**Scheme 61:**
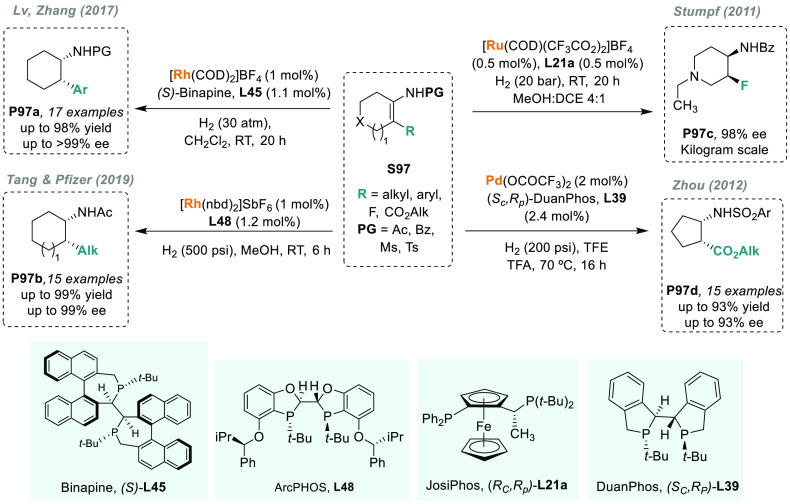
Metal-Catalyzed AH of Tetrasubstituted Cyclic Enamides

In 2017, Lv, X. Zhang, and co-workers developed a highly enantioselective hydrogenation of cyclic *N*-acyl enamines **S97** to provide optically pure cycloalkyl amides **P97a** using Rh-Binapine (**L45**) as catalyst (Scheme 61).^377^ The resulting chiral amides had an aryl substituent in the vicinal position. The methodology could be applied to prepare biologically active compounds. More recently, in 2019, Tang, in collaboration with a team from Pfizer, demonstrated that an electron-rich *P*-stereogenic bisphosphorus ligand with deep chiral pockets (ArcPHOS, **L48**) could be applied to the rhodium-catalyzed AH of **S97** bearing alkyl substituents at the 2-position (Scheme 61).^378^ Consequently, chiral amides **P97b** were attained in excellent yields and enantioselectivities. The methodology was showcased by a concise synthesis of Tofacitinib (Figure 7). Previously, Stumpf and co-workers reported a multikilogram scale asymmetric synthesis of the enantiomerically pure fluoropiperidine **P97c** via AH using a Ru/JosiPhos (**L21a**) catalyst with high enantiomeric excesses (Scheme 61).^379^ This fluorinated aminopiperidene is also present as a structural motif in the antibacterial clinical candidate AZD9742. In fact, researchers at AstraZeneca have recently reported its enantioselective synthesis by means of AH using [((*S*)-BINAP)RuCl_2_](*p*-cymene).^380^

Chiral cyclic β-amino acids,^381^ such as cispentacin (Figure 7), are important in the synthesis of β-peptides. In 2003, X. Zhang and co-workers pioneered the AH of tetrasubstituted cyclic β-(acylamino)acrylates using the chiral biaryl ligand C_3_-TunaPhos.^382^ Following this path, Zhou’s group hydrogenated tetrasubstituted cyclic β-(arylsulfonamido)acrylates. Using Pd/DuanPhos-**L39** as catalyst, a range of five-membered chiral β-amino acid derivatives **P97d** were obtained in excellent yields and enantiomeric excesses (Scheme 61).^383^ More recently, the same group described the asymmetric hydrogenation of carbocyclic aromatic amines using a ruthenium-DuPhos (**L40b**) complex as catalyst.^384^

### 3.3 *N*-Sulfonyl Enamines

**Scheme 62:**
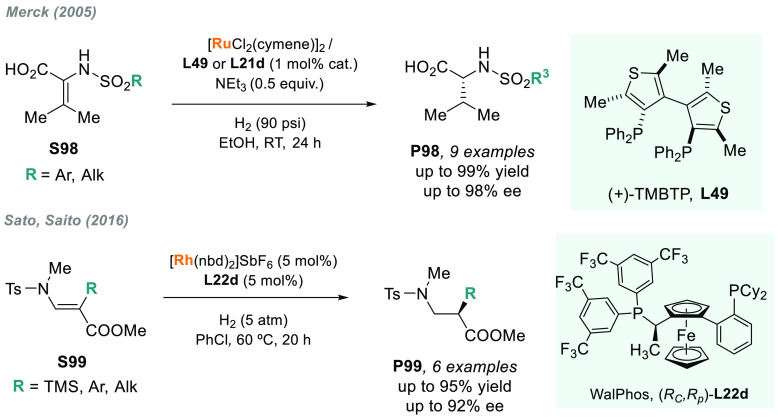
Metal-Catalyzed AH of α- and β-(Arylsulfonamido)acrylates

Little attention has been devoted to the AH of *N*-sulfonyl enamines, and only a few examples can be found in the literature.^385,386^ Following the latter example (**P97d**, Scheme 61), the enantioselective synthesis of chiral amino acids via AH of noncyclic α- and β-(arylsulfonamido)acrylates is of great importance. In 2005, a team from Merck published the synthesis of an anthrax lethal factor inhibitor via ruthenium-catalyzed AH of **S98** (Scheme 62).^387^ Catalyst screening identified that JosiPhos **L21d** and the bis-thiophene atropoisomeric ligand **L49** gave excellent enantioselectivities. Later, in 2016, Sato, Saito, and co-workers reported nickel-promoted regioselective carboxylation of internal enamides to afford a range of α-substituted β-aminoacrylates **S99**. These were then subjected to the rhodium-catalyzed AH using Walphos ligand **L22d**. Chiral amino acid derivatives **P99** were furnished in a highly enantioselective manner (Scheme 62).^388^ On the other hand, the AH of cyclic *N*-sulfonyl enamines is rare. To the best of our knowledge, there is only one example in the literature, reported by Andersson’s group, using iridium complexes bearing chiral P,N ligands.^389^ However, the method was hampered by low conversions and moderate enantioselectivities with a very narrow scope.

### 3.4 Other Enamides

**Scheme 63:**
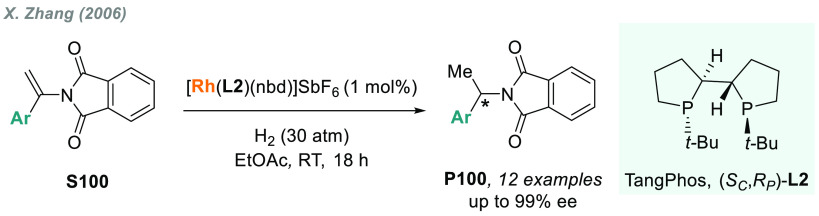
Rhodium-Catalyzed AH of *N*-Phthaloyl Enamides

The AH of *N*-phthaloyl enamides is a promising method for the preparation of chiral amines. Apart from serving as a directing group, the phthalimido functionality can be easily removed under mild conditions. In 2006, X. Zhang and co-workers reported the highly enantioselective hydrogenation of α-aryl *N*-phthaloyl enamides using rhodium catalysts derived from TangPhos (**L2**) (Scheme 63).^390^ The resulting chiral α-methyl, aryl *N*-phthalimides **P100** were generally obtained in excellent enantioselectivities, but these dramatically decreased for substrates bearing an *ortho*-substituent on the aromatic ring. In contrast, the preparation of enantioenriched α-methyl, alkyl *N*-phthalimides using AH has not yet been explored. Later, the authors reported the use of the same chiral ligand **L2** in the rhodium-catalyzed AH of *N*-phthaloyl dehydroamino acid esters, thus affording highly valuable chiral α- or β-amino acid derivatives with good to excellent enantioselectivities.^391^

**Scheme 64:**
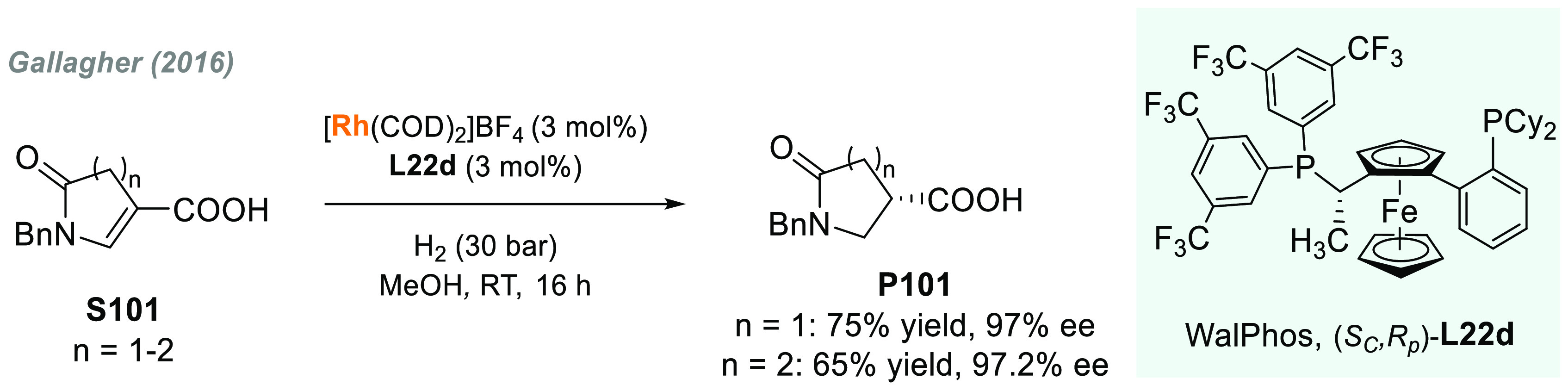
Metal-Catalyzed AH of Lactams

The rhodium-catalyzed AH of heterocyclic β-aminoacrylates **S101** was accomplished by Gallagher and co-workers in 2016 (Scheme 64).^392^ By using WalPhos **L22d** as a chiral ligand, several pyrrolidine and piperidone variants were efficiently hydrogenated, providing chiral heterocyclic amino acids **P101** with high enantioselectivity. The use of the carboxylic acid was essential for the success of the reaction. Similarly, the AH of β,γ-unsaturated γ-lactams was described by Liu, W. Zhang, and co-workers, although the hydrogenation proceeded via *N*-acyliminium cations.^196^

On the other hand, Ding, Han, and co-workers recently described the double asymmetric hydrogenation of 3,4-dialkylidene-2,5-diketopiperazines using an iridium-SpinPhox (**C4**) complex as catalyst.^393^

**Scheme 65:**
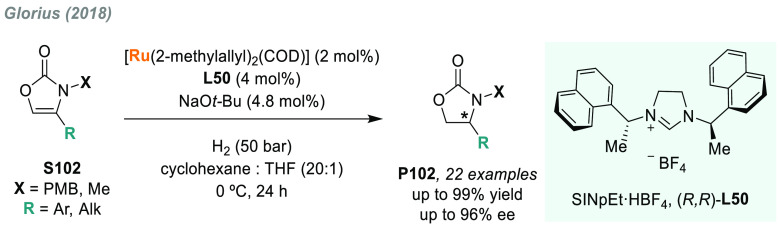
Ruthenium-Catalyzed AH of 2-Oxazolones

The enantioselective synthesis of chiral 2-oxazolidinones, widely used as Evans’ chiral auxiliaries, has attracted considerable attention for the construction of new chiral building blocks and the development of new asymmetric transformations. An alternative to the conventional approach, limited to easily accessible chiral β-amino alcohols, is the direct AH of 2-oxazolones. In this regard, in 2018, Glorius and co-workers reported an innovative protocol for the ruthenium-catalyzed AH of **S102** using **L50** as precursor of the NHC ligand (Scheme 65).^394^ A variety of chiral 2-oxazolidinones **P102** were obtained in excellent enantioselectivities (up to 96% ee). The formal synthesis of (−)-aurantioclavine was demonstrated as a synthetic application. X. Zhang’s group previously reported the rhodium-catalyzed AH version of this transformation using TangPhos **L2**, albeit with moderate enantioselectivities and restricted mainly to substrates bearing electron-donating groups on the aryl ring.^395^

## 4 Asymmetric Hydrogenation of Enamines

Although great progress has been made in the transition metal-catalyzed asymmetric hydrogenation of *N*-protected enamides, the introduction and removal of the protecting group reduce the overall efficiency of the method and limit its applications in the synthesis of optically active amines. To overcome this drawback, significant efforts have been devoted to the development of new chiral catalysts for the direct AH of enamines, including *N*-alkyl, *N*-aryl, or unprotected amines.^40^

### 4.1 *N*-Alkyl Enamines

**Scheme 66:**
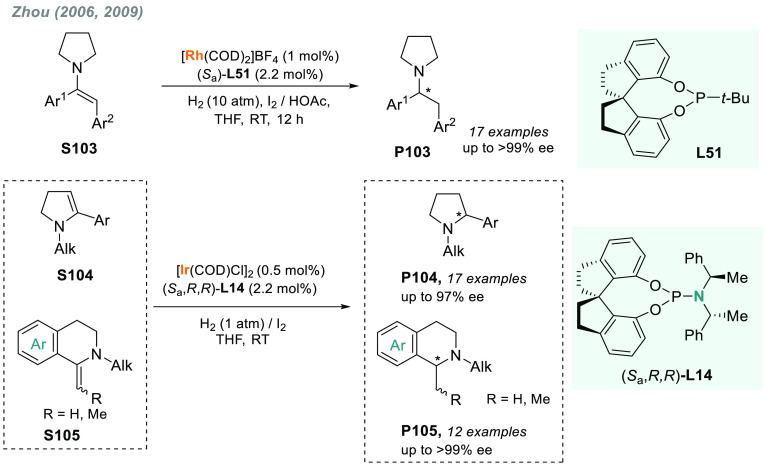
Metal-Catalyzed AH of Endocyclic and Exocyclic *N*-Alkyl Enamines

In 2006, Zhou pioneered the AH of cyclic enamines using rhodium catalysts bearing monophosphorus ligands. In particular, spiro-phosphinite ligand **L51** showed excellent enantioselectivites for simple *N,N*-dialkyl enamines **S103** (Scheme 66).^396,397^ The reaction rates were enhanced using I_2_/acetic acid as additives. Later, in 2009, the same group reported that other *N*-alkylated enamines could be efficiently hydrogenated using a similar catalytic system Ir/**L14**/I_2_ (Scheme 66). This protocol allowed the hydrogenation of both endocyclic^398^ (**S104**) and exocyclic^399^ (**S105**) enamines in excellent enantioselectivities under very mild conditions (1 bar of H_2_ and RT). Pfaltz also contributed to the field using phosphino-oxazoline ligands for the iridium-catalyzed AH of **S103**-type substrates, albeit with lower ee values.^400^

**Scheme 67:**
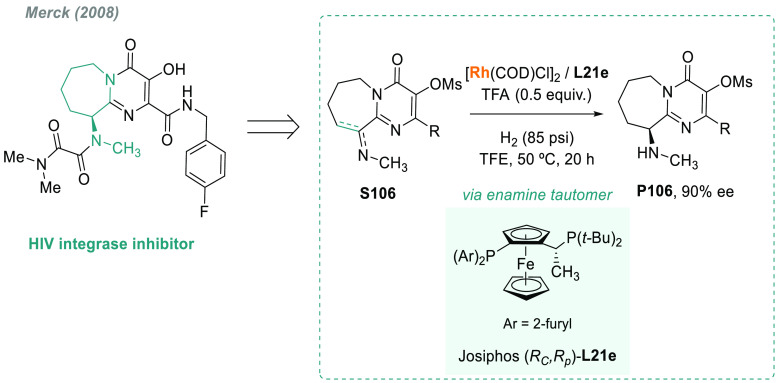
Catalytic Synthesis of an HIV Integrase Inhibitor

In 2009, a team from Merck applied the direct AH of alkylated enamines to the synthesis of an HIV integrase inhibitor (Scheme 67).^401^ Using Rh and a JosiPhos ligand (**L21e**), a mixture of imine/enamine **S106** was efficiently hydrogenated, affording **P106**, a direct precursor of the target drug, in 90% ee. Interestingly, a deuterium labeling study suggested that the AH proceeds predominantly via the enamine tautomer.

### 4.2 *N*-Aryl Enamines

**Scheme 68:**
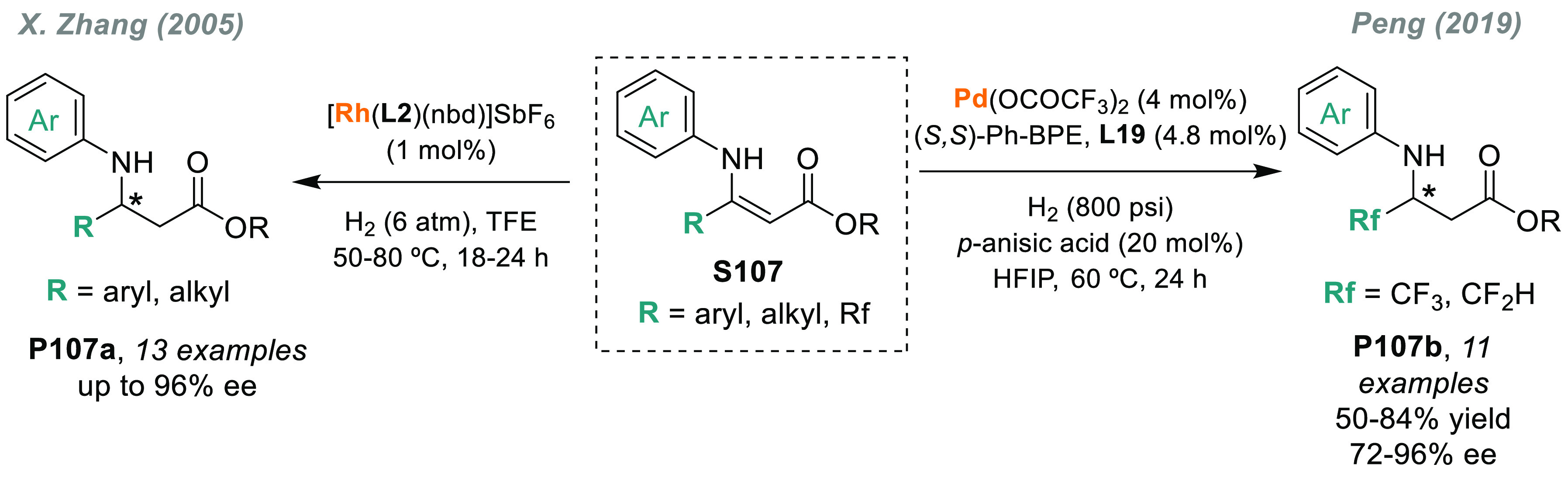
Metal-Catalyzed AH of *N*-Aryl Enamines

The enantioselective synthesis of *N*-aryl β-enamino esters was first studied in 2005, when X. Zhang and co-workers developed an enantioselective strategy based on the rhodium-catalyzed AH of *N*-aryl enamines **S107** (Scheme 68).^402^ Chiral *N*-aryl-substituted β-amino acid derivatives **P107a** were obtained in moderate to excellent enantioselectivities (79–96% ee) using a P-stereogenic ligand (TangPhos, **L2**). However, the reaction was highly substrate-dependent, and **S107** bearing a CF_3_ as high electron-withdrawing group showed poor conversion. To overcome this limitation, Peng recently reported an alternative approach using Pd/**L19** as catalyst.^403^ Chiral β-fluoroalkyl β-amino acid derivatives **P107b** were obtained in good yields and excellent enantioselectivites (Scheme 68). The use of *p*-anisic acid, which can promote the tautomeric transformation between imines and enamines, enhanced both the activity and enantioselectivity. The authors speculated that the hydrogenation could occur through an asymmetric reduction of the iminium ion rather than the enamine form of the substrate.

**Scheme 69:**
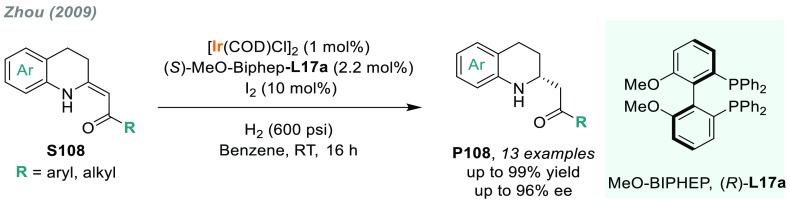
Iridium-Catalyzed AH of Exocyclic *N*-Aryl Enamines

In 2009, Zhou and co-workers described the first highly enantioselective AH of exocyclic unprotected enamines **S108** by using Ir/(*S*)**-L17a**/I_2_ as catalytic system (Scheme 69).^404^ The resulting chiral amines **P108** were afforded in excellent yields and up to 96% ee.

### 4.3 Unprotected Enamines

**Scheme 70:**
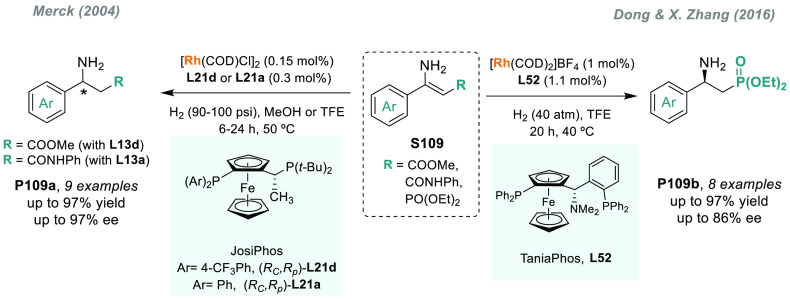
Rhodium-Catalyzed AH of β-Functionalized Enamines

The AH of unprotected enamines has scarcely been studied because the transition metal catalyst is usually poisoned by the nucleophilic amino group. However, the direct AH of these substrates is highly desirable to avoid redundant introduction and subsequent removal of protecting groups and also for the preparation of pharmacologically relevant compounds. In 2004, a team from Merck reported the first example of catalytic AH of unprotected β-enamine esters and amides (**S109**), using Rh-JosiPhos complexes as catalysts (Scheme 70).^405^ Ligand **L21d** gave the best results in the AH of enamine esters, while **L21a** gave the highest rates and enantioselectivities for the AH of enamine amides. β-Amino acids derivatives **P109a** were attained in excellent yields and enantioselectivities, thus proving that the *N*-acyl group is not always a prerequisite for such transformations.

**Scheme 71:**
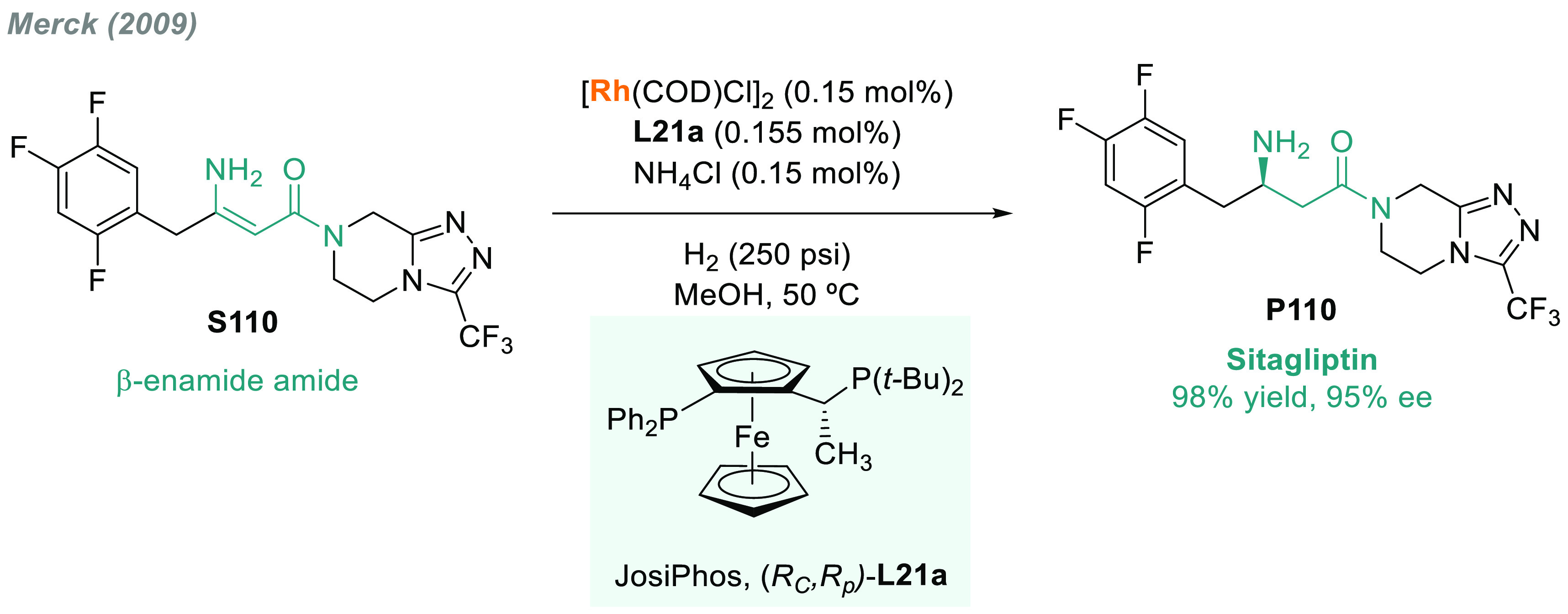
Asymmetric Synthesis of Sitagliptin via Rhodium-Catalyzed AH

The applicability of this protocol in late-stage functionalization was showcased by the asymmetric synthesis of Sitagliptin (**P110**, Scheme 71), which was implemented on a manufacturing scale.^58^**P110** was obtained in 98% yield and 95% ee (improved to >99% ee by recrystallization) using *(R*_*C*_*,R*_*p*_*)***-L21a**. More recently, Chikkali and co-workers reported that Rh complexes bearing chiral FerroLANE ligands also catalyze the AH of **S110** to yield sitagliptin with excellent enantioselectivity (98% ee).^406^ The asymmetric synthesis of **P110** was also accomplished via direct asymmetric reductive amination (ARA) with unprecedented levels of asymmetric induction.^407^ In addition, Ru catalysis has been successfully applied in both ARA or direct AH of unprotected enamines for the preparation of other pharmacologically relevant compounds.^408,409^

The rhodium-catalyzed AH of unprotected β-enamine phosphonates was described by Dong, X. Zhang, and co-workers (Scheme 70).^410^ By using Rh/TaniaPhos-**L52**, the method provided an efficient route to free β-amino phosphonates **P109b**, which are important intermediates in biochemistry and pharmaceuticals. This work provided an alternative to the protocol previously reported by Ding, in which the amine was necessarily protected by acyl groups (Scheme 53).^332^ Also, Ruchelman et al. reported the enantioselective hydrogenation of unprotected β-aminosulfones using Rh catalysis, which afforded a key intermediate of the phosphodiesterase 4 (PDE4) inhibitor Apremilast.^411^

**Scheme 72:**
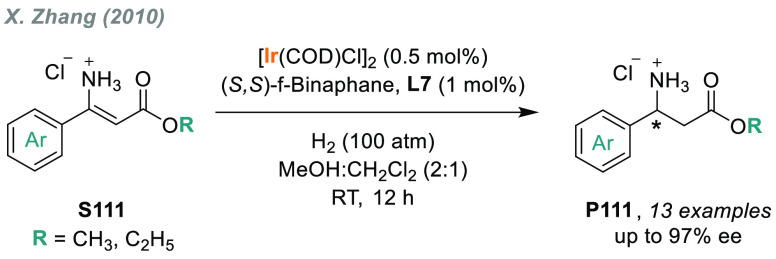
Iridium-Catalyzed AH of β-Enamine Hydrochloride Esters

Iridium catalysis has also been applied in the AH of unprotected enamines. X. Zhang demonstrated that β-enamine hydrochloride esters **S111** can be suitable substrates for AH, despite the fact that primary amines might have a strong inhibitory effect on the iridium catalyst.^412^ The combination of Ir/f-binaphane (**L7**) and the use of hydrochlorides provided direct access to a range of enantiomerically enriched β-amino acids without use of an amino protecting group (Scheme 72).

## 5 Asymmetric Hydrogenation of Allyl Amines

**Figure 8:**
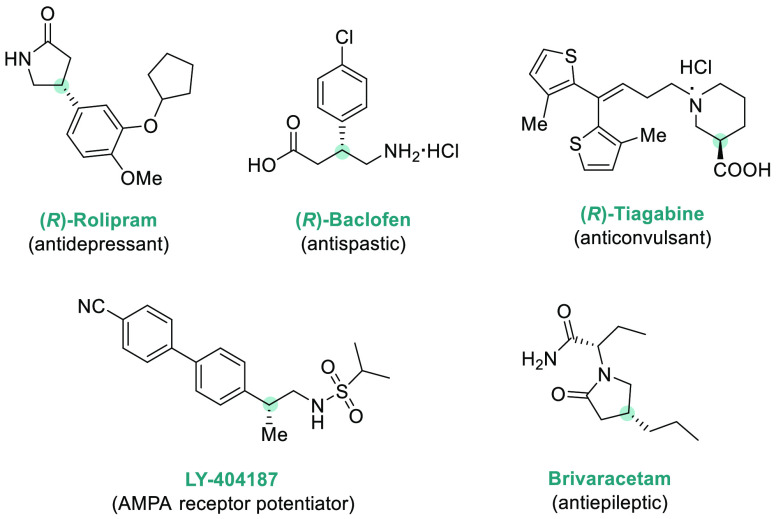
Representative drugs that can be prepared via the AH of allyl amines.

The AH of allyl amines remained relatively underdeveloped before the past decade. Allyl amines are usually considered minimally functionalized olefins as the unsaturated bond lacks close coordinating groups. For this reason, the AH of allyl amines is more challenging if compared to imines or enamines. In the early past decade, a range of new strategies were described for the metal-catalyzed AH of allyl amines, exhibiting high reactivity and enantiocontrol. Thus, the preparation of highly valuable β- and γ-substituted chiral amines is now more accessible. In this section, the most important precedents in the field will be described, along with pharmaceuticals and drugs that can be attained by means of the AH of allyl amines (Figure 8).

### 5.1 *N*-Phthaloyl Allyl Amines

**Scheme 73:**
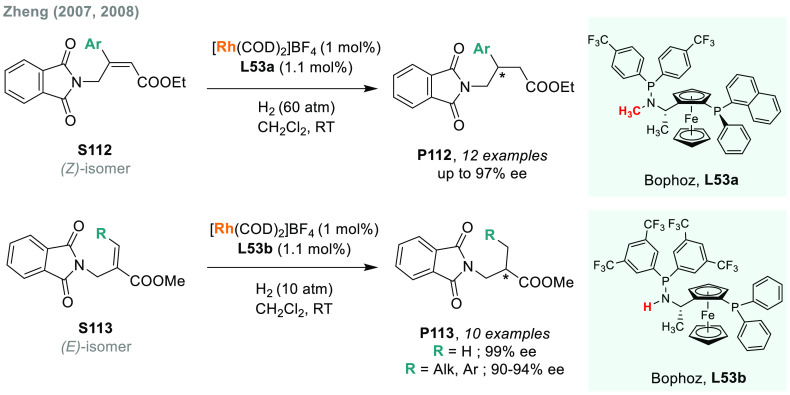
Rhodium-Catalyzed AH of β- and γ-Phtalimido-Substituted Unsaturated Esters

As previously stated, chiral β- and γ-amino acids and their derivatives are important building blocks in the synthesis of pharmaceuticals and other bioactive compounds. Zheng and co-workers reported the first highly enantioselective synthesis of chiral β-aryl-γ-amino acid ester derivatives **P112** via rhodium-catalyzed AH of γ-phthalimido-substituted acrylates^413^ using the BoPhoz-type ligand^414,415^**L53a** (Scheme 73). The method showed high reactivity and enantioselectivity (up to 97% ee) for a range of (*Z*)-substrates **S112**. The method was successfully applied to the synthesis of several chiral pharmaceuticals including (*R*)-rolipram and (*R*)-baclofen (Figure 8) with high enantioselectivities. The same group later expanded the applicability of this approach to the asymmetric synthesis of β^2^-amino acids **P113** via rhodium-catalyzed AH using another ligand of the BoPhoz family: **L53b** (Scheme 73).^416^ Interestingly, the presence of an N–H proton in the ligand significantly improved the enantioselectivity, whereas the introduction of a *P*-stereogenic center in the phosphino moiety proved unfruitful and displayed low conversion. The same catalytic system also exhibited excellent ee values for β-unsubstituted substrates **S113** (99% ee). Other protocols for the stereoselective synthesis of chiral β^2^-amino acids include the rhodium-catalyzed AH of β-substituted α-aminomethyl acrylates that Börner and co-workers^417,418^ and Qiu and co-workers^419,420^ independently reported in this earlier past decade.

**Scheme 74:**
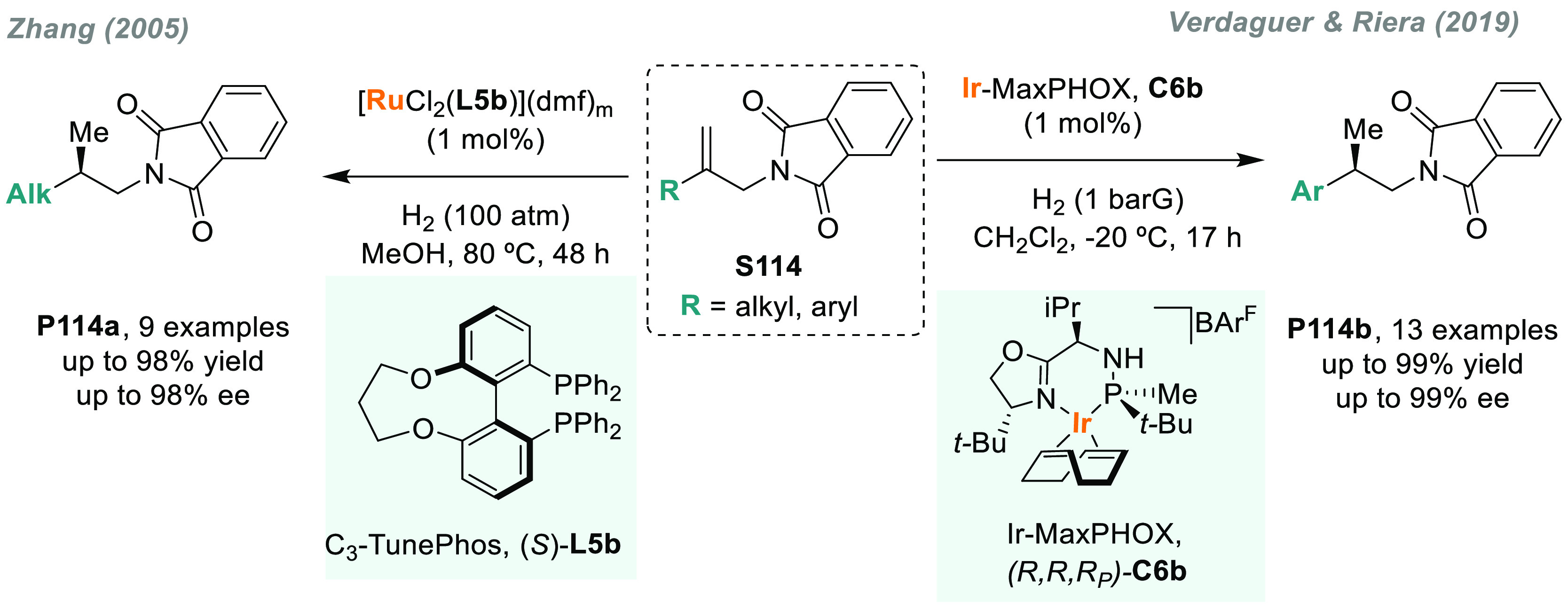
Metal-Catalyzed AH of *N*-Allyl Phthalimides

Chiral amines bearing a β-methyl stereogenic center are extremely interesting as they are present in numerous drugs and pharmaceuticals. The AH of 2-substituted allyl phthalimides is an efficient route toward their preparation. X. Zhang pioneered the field with the ruthenium-catalyzed AH of terminal disubtituted allylphthalimides **S114** using *C*_3_-TunePhos ligand **L5b** (Scheme 74).^421^ Chiral β-alkyl-β-methyl amines **P114a** were attained in excellent yields and enantioselectivities. However, the scope of this reaction was limited to alkyl substituents, as the hydrogenation of an aromatic substrate gave moderate enantioselectivity. To overcome this limitation, Verdaguer and Riera’s laboratory recently reported the highly enantioselective hydrogenation of 2-aryl allyl phthalimides using iridium catalysis (Scheme 74).^422^ Ir-MaxPHOX **C6b** bearing a bulky substituent on the oxazoline ring gave the best enantioselectivities (>99% ee in the best cases for **P114b**), showing an exquisite functional group tolerance for a range of substrates. Several direct synthetic applications of this catalytic method were disclosed, such as the formal synthesis of (*R*)-Lorcaserin (Figure 1), which is a marketed anorectic drug, and also a novel approach to enantiomerically enriched 3-methyl indolines.

### 5.2 *N*-Sulfonyl Allyl Amines

**Scheme 75:**
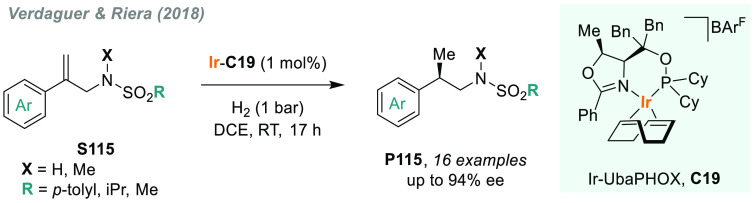
Iridium-Catalyzed AH of 2-Aryl *N*-Sulfonyl Allyl Amines

Verdaguer and Riera’s group also reported the iridium-catalyzed AH of *N*-sulfonyl allyl amines **S115**,^423^ which can be easily prepared by the iridium-catalyzed isomerization of *N*-tosylaziridines.^424^ By using the commercially available iridium catalyst UbaPHOX (**C19**), first reported by Pfaltz,^425,426^ a wide range of chiral β-methyl amines were afforded with good to excellent enantioselectivities (Scheme 75). These compounds are also key intermediates for the preparation of allosteric modulators of AMPA receptor such as LY-404187 (Figure 8).^427^

**Scheme 76:**
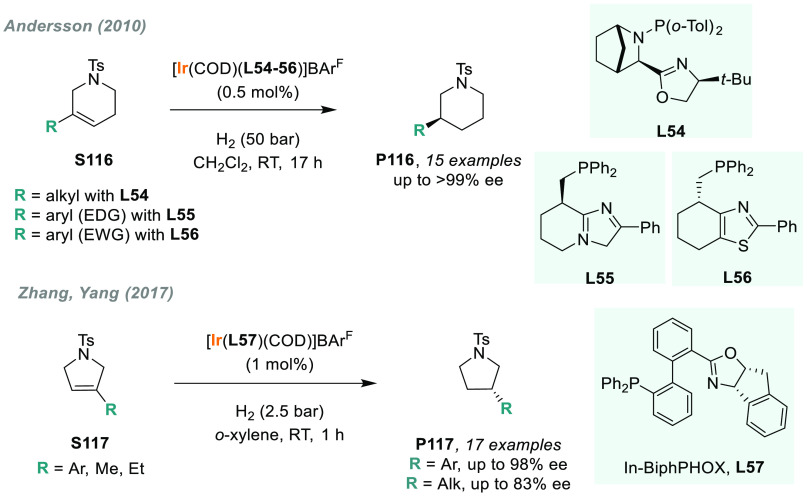
Iridium-Catalyzed AH of Cyclic *N*-Sulfonyl Allyl Amines

Iridium complexes bearing chiral *P*,*N* ligands were also applied to the catalytic hydrogenation of cyclic *N*-sulfonyl allyl amines **S116**, as reported by Andersson and co-workers.^428,389^ The reaction was highly substrate-dependent, and an appropriate chiral ligand was used for each case. Substrates **S116** bearing aliphatic substituents were efficiently hydrogenated using a phosphino-oxazoline ligand **L54** whereas phosphino-imidazole **L55** and phosphino-thiazole **L56** gave excellent activities and selectivities for aromatic substrates, depending on their electronic properties (Scheme 76). In addition, the methodology was further expanded to the iridium-catalyzed AH of five- and seven-membered *N*-heterocyclic olefins. The chiral pyrrolidines, piperidines, and azepanes, which are highly valuable motifs for the synthesis of medicinal compounds and natural products, were attained in excellent enantioselectivities. Similarly, W. Zhang and co-workers recently reported the catalytic AH of 3-substituted 2,5-dihydropyrroles (**S117**) using an Ir-catalyst with an axially flexible chiral phosphine-oxazoline ligand named BiphPhox (**L57**).^429^ Chiral *N*-tosyl pyrrolidines **P117** were efficiently prepared in good to excellent enantioselectivities (Scheme 76).

### 5.3 Other Allyl Amines

**Scheme 77:**
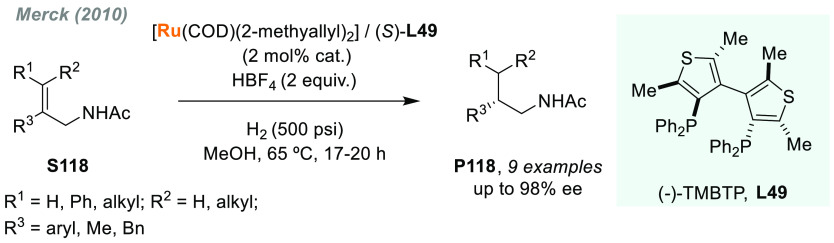
Ruthenium-Catalyzed AH of *N*-Acyl Allyl Amines

During the past decade, other remarkable examples have also been reported for the highly enantioselective hydrogenation of protected allyl amines. In 2010, a team from Merck described a general method for the ruthenium-catalyzed AH of trisubstituted *N*-acyl allyl amines **S118** using the axially chiral ligand **L49**, affording chiral β-substituted amines **P118** with excellent enantioselectivities (Scheme 77).^430^

**Scheme 78:**
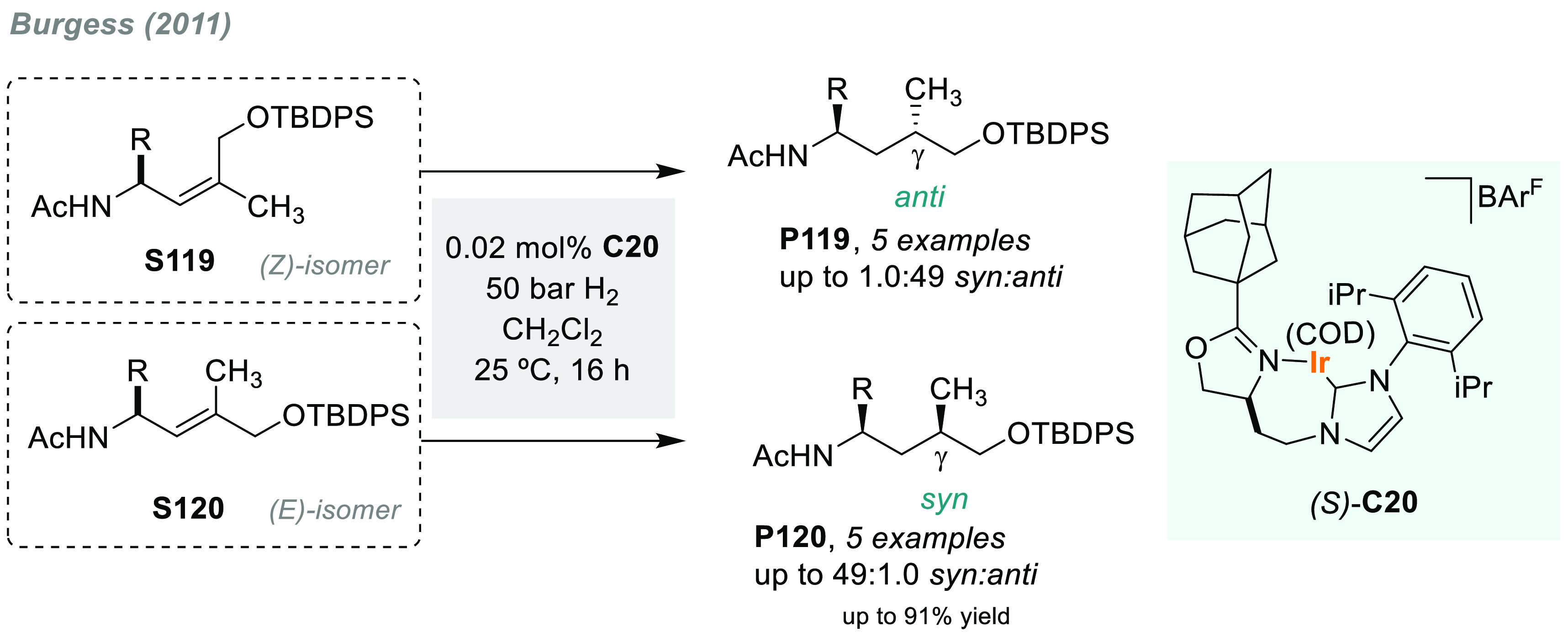
Iridium-Catalyzed AH of *N*-Acyl Allyl Amines

Optically active amines with remote stereocenters, such as γ-substituted chiral amines, are often key contributors to the potent biological activity of many natural products and pharmaceuticals. Important examples of this class of compounds are dexbrompheniramine, which is an antihistaminic, and tolterodine, an anticholinergic (Figure 1). γ-Amino acids such as γ-aminobutyric acid (GABA) are also important in medicinal chemistry. Consequently, asymmetric synthesis of chiral derivatives of GABA with appropriate side chains is potentially important for the design of new drug-like molecules with enhanced pharmacological properties. Nevertheless, the direct preparation of γ-substituted chiral amines remains underdeveloped compared to the well-established methods for constructing α- and β-substituted chiral amines. In addition, most of the enantioselective syntheses of these compounds are indirect and often require multiple steps. Buchwald’s group first reported a direct approach to γ-chiral amines by enantioselective CuH-catalyzed reductive relay hydroamination.^431^ Hull and co-workers developed efficient conditions for the highly enantioselective synthesis of γ-branched amines via rhodium-catalyzed reductive amination.^432^ Alternatively, the redox neutral asymmetric isomerization of allylic amines is a method of choice with an excellent atom economy, although current methods are hampered by very limited substrate scope.^17,433^ In this scenario, the development of new transition metal-catalyzed AH processes to afford enantioenriched γ-substituted amines is extremely desirable. In 2011, Burgess and co-workers reported the asymmetric synthesis of α-methyl-γ-amino acid derivatives via catalytic AH using a carbene–oxazoline iridium complex **C20** (Scheme 78).^434^ The method of optimization was based on varying peripheral aspects of the substrate rather than optimizing the catalyst via ligand modifications. Following this approach, *O*-TBDPS-protected allylic substrates gave the best results. Once hydrogenated under mild conditions, chiral γ-methyl amines were afforded in high stereocontrol. *Anti*-products **P119** were formed from the *Z*-alkenes **S119**, while the *E*-isomers **S120** gave *syn*-target compounds **P120**.

**Scheme 79:**
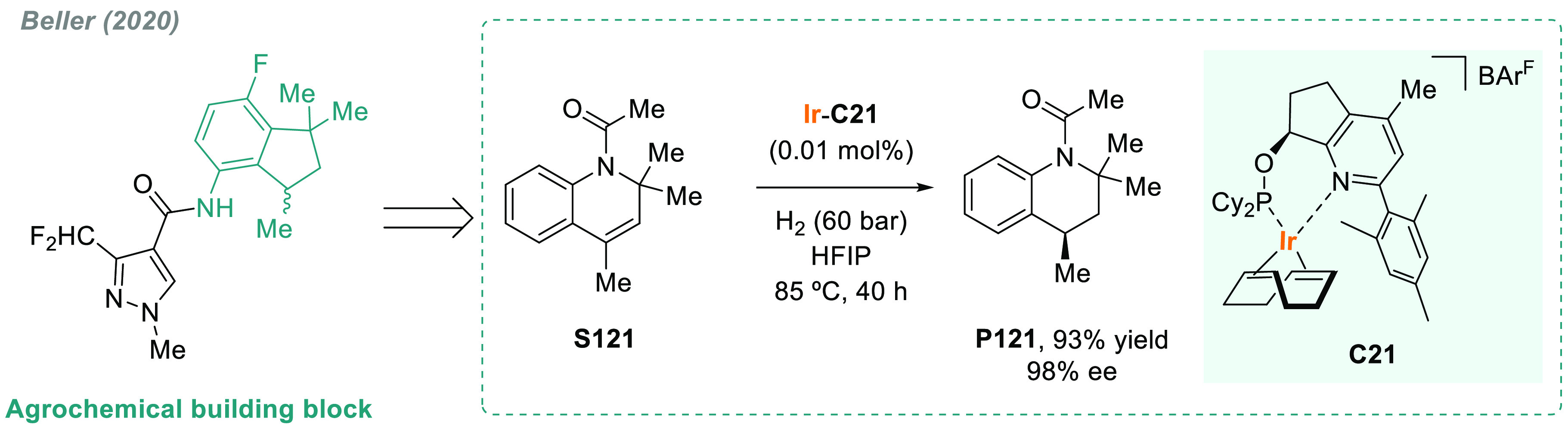
Synthesis of an Agrochemical Building Block via AH

Similarly, Beller and co-workers recently used **C21**, an iridium-*P*,*N*-ligand complex, for the AH of *N*-acyl endocyclic allyl amines **S121** (Scheme 79).^435^ Using HFIP as reaction solvent, the reaction proceeded efficiently to afford chiral γ-methyl amide **P121**, which is a key intermediate for the preparation of a common agrochemical building block.

**Scheme 80:**
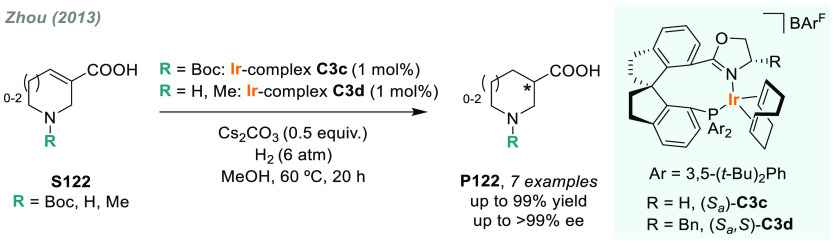
Iridium-Catalyzed AH of Endocyclic *N*-Allyl Amines

**Scheme 81:**
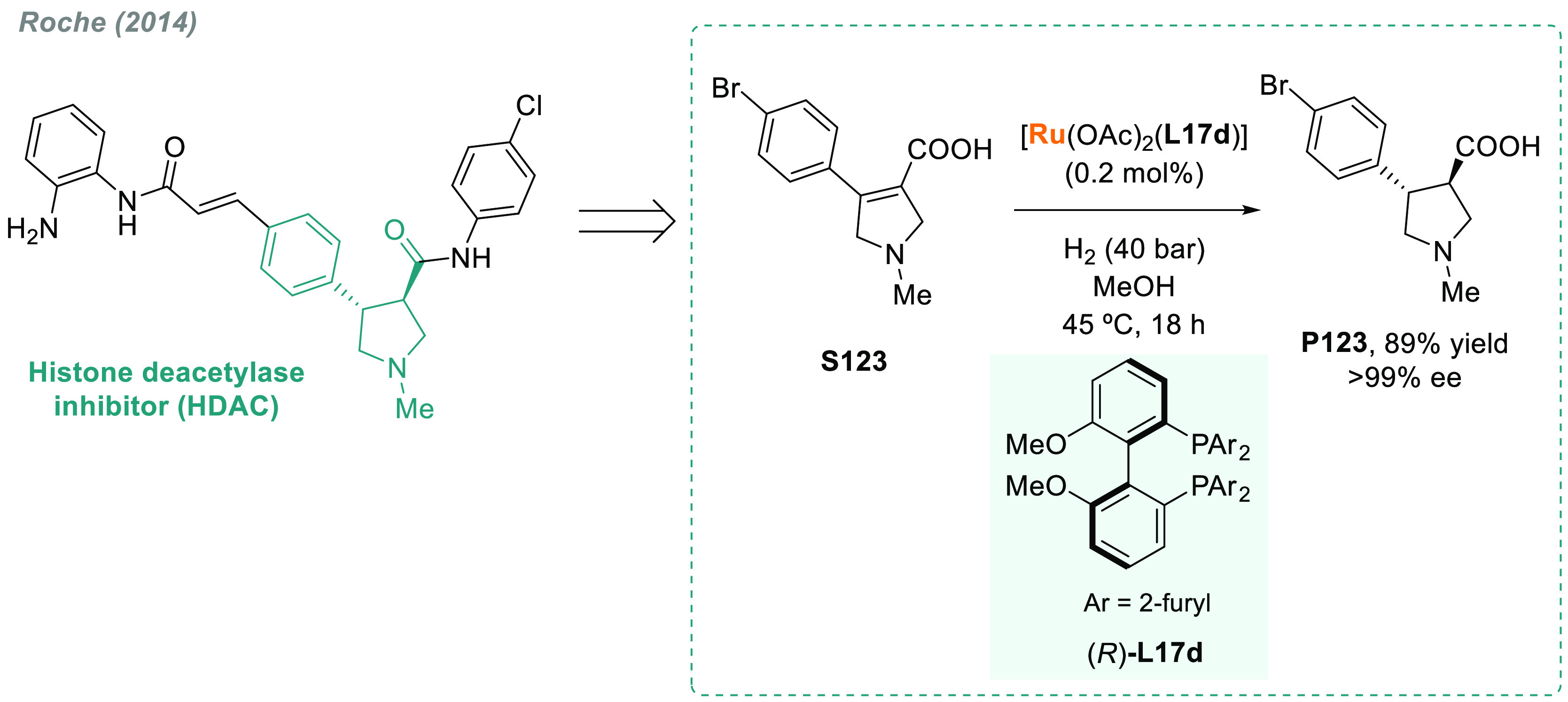
Asymmetric Synthesis of a HDAC Inhibitor via AH

Another type of endocyclic allyl amines, amino acids **S122**, were hydrogenated by Zhou and co-workers using iridium complexes of *P*,*N*-ligands with a spiro backbone (**C3c** and **C3d**) (Scheme 80).^436^ In particular, **C3c** was chosen as the best catalyst for *N*-Boc allyl amines, while **C3d** was used for *N*-Me and unprotected allyl amines. The resulting chiral heterocyclic acids **P122** were afforded in excellent enantioselectivities, and the potential utility was demonstrated by the concise asymmetric synthesis of (*R*)-nipecotic acid and (*R*)-tiagabine (Figure 8). In fact, chiral cyclic amines with a β-carboxylic acid or derivatives are common structural motifs in many bioactive compounds. For example, the key intermediate for the preparation of a histone deacetylase inhibitor (HDAC, Scheme 81) was obtained via the ruthenium-catalyzed AH of **S123** using **L17d**.^437^ This achievement was reported by a team from Roche in 2014. They disclosed that the presence of a carboxylic acid was crucial for the rapid hydrogenation of the unsaturated bond.

**Scheme 82:**
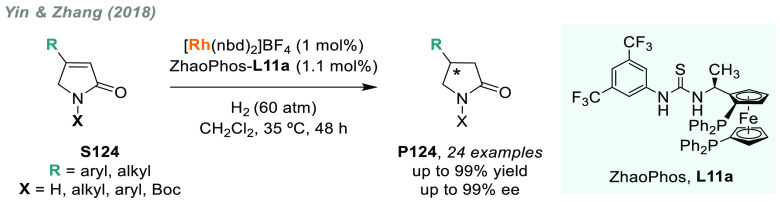
Rhodium-Catalyzed AH of β-Aryl-Substituted α,β-Unsaturated Lactams

Chiral γ-lactams are ubiquitous in biological compounds. The antiepilepsy drug Brivaracetam and the PDE-4 inhibitor Rolipram are examples of γ-lactam clinical drugs (Figure 8). These lactams are key building blocks in medicinal chemistry as masked γ-amino acids. In their efforts to prepare γ-lactams in optically pure form, Yin, X. Zhang, and co-workers recently developed the AH of **S124** using Rh/ZhaoPhos-**L11a** (Scheme 82).^438^ A wide range of enantioenriched γ-lactams **P124** were furnished in excellent yields and enantioselectivities. Interestingly, the catalytic system successfully tolerated a free NH amide group which actually played a positive effect by hydrogen bonding with the thiourea motif of **L11a**.

**Scheme 83:**
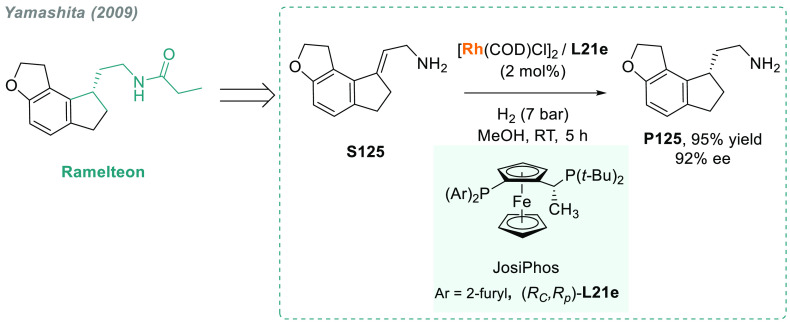
Rhodium-Catalyzed AH of Unprotected Primary Allyl Amine

Another important drug with a chiral center in the γ-position of the amino group is ramelteon, a selective melatonin MT1/MT2 receptor agonist. Yamashita’s laboratory reported that Rh/JosiPhos-**L21e** was a highly effective catalyst for the AH of a key precursor, the unprotected allyl amine **S125** (Scheme 83).^439^ Interestingly, the primary amino group might act as an anchoring group to the rhodium atom. Chiral amine **P125** was smoothly prepared in 92% ee using very mild conditions.

**Scheme 84:**
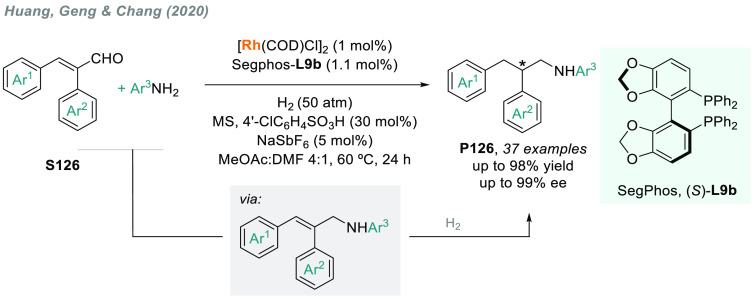
Combination of AH and Reductive Amination

Finally, Huang, Geng, Chang, and co-workers recently reported a practical combination of AH and reductive amination in the enantioselective synthesis of the chiral β-aryl amines **P126** (Scheme 84). Starting from readily available anilines and α,β-unsaturated aldehydes **S126**, they described a one-pot hydrogenation that involved DRA and AH, using Rh/**L9b** complex as catalyst.^440^ Control experiments revealed that the construction of the C–N bond beforehand helped to pave the way for the subsequent AH of the corresponding *N*-allyl amine.

## 6 Asymmetric Hydrogenation of Heteroaromatic Compounds

The direct AH of heteroaromatic compounds provides straightforward access to the corresponding chiral saturated *N*-cyclic skeletons. This is a less explored area than the AH of prochiral unsaturated amines such as imines or enamides.^30−32,441^ This may be ascribed to several reasons: (a) the possible deactivation of the chiral catalysts due to the basicity of the nitrogen atom of the aromatic ring; (b) the lack of a coordinating group in simple aromatic compounds; and (c) the increased stability due to the aromaticity of these compounds, which might require harsher conditions. Despite all these issues, remarkable advances have been disclosed during the past decade. In this section, we will review these advances along with the pioneering works and research milestones in this field, focusing mainly on heteroarenes such as quinolines, pyridines, quinoxalines, and indoles.

### 6.1 Quinolines and Derivatives

Optically pure tetrahydroquinolines (THQs) and their derivatives are of great importance due to their pharmaceutical and agrochemical applications,^442^ as they are basic units in many natural products. Among the different strategies for their preparation, the AH of quinolines is the most effective.^443^

**Scheme 85:**
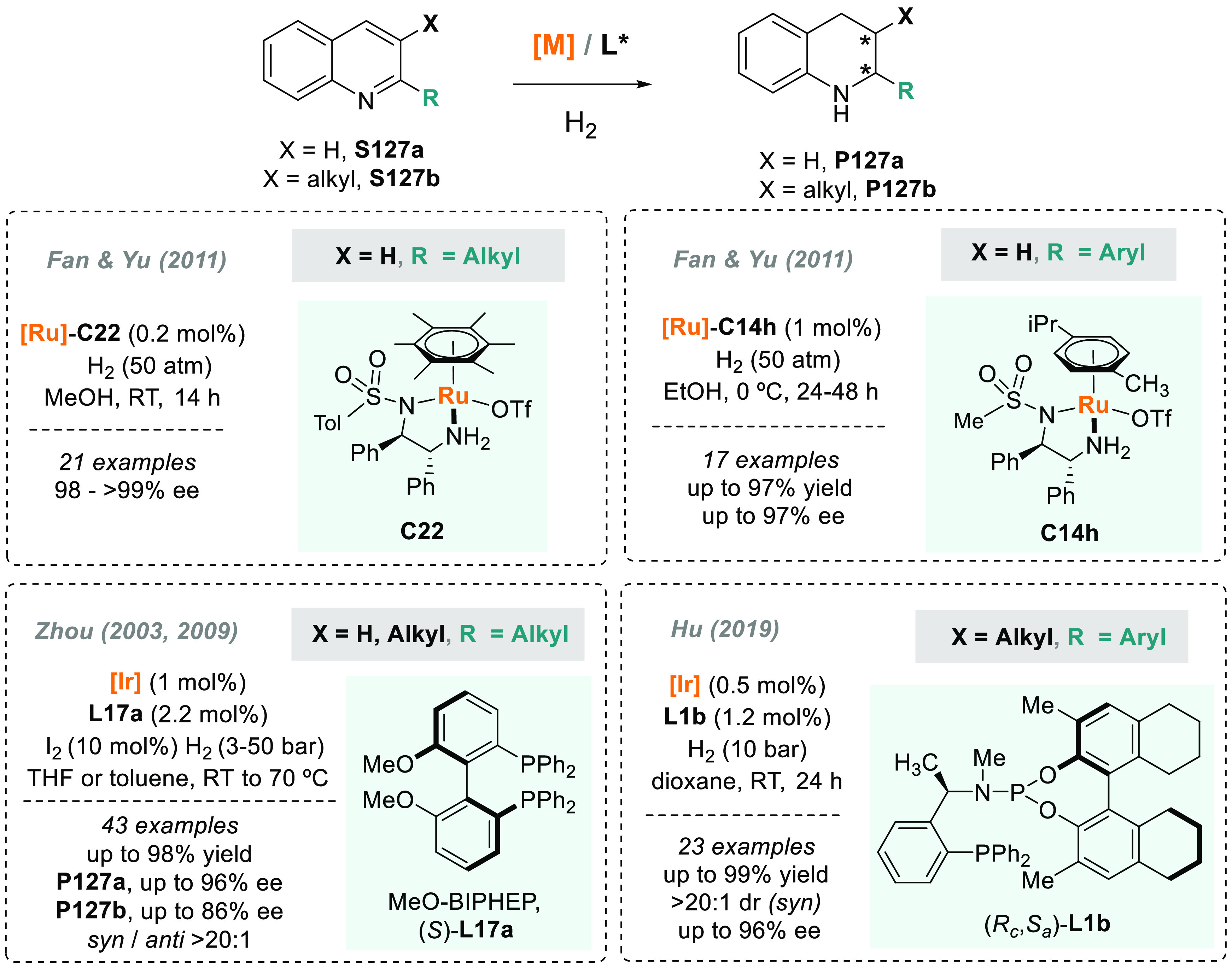
Metal-Catalyzed AH of Unfunctionalized 2-Quinolines and 2,3-Disubstituted Quinolines

In 2011, Fan, Yu, Chan, and co-workers reported the AH of a wide range of quinoline derivatives (**S127**) catalyzed by chiral cationic η^6^-arene Ru(II)-diamine complexes (Scheme 85).^444^ Interestingly, for 2-alkylquinolines, **C22** exhibited outstanding enantioselectivity as **P127a** (R = alkyl) were attained in excellent enantioselectivities (99% ee for most of the cases). To the best of our knowledge, this is the protocol with the highest level of enantioselectivity for 2-alkyl-substituted quinolines **S127a**. In contrast, the AH of 2,3-dialkyl quinolines **S127b** provided a very low ratio of diastereoselectivity. The same authors reported that similar Ru-diamine complexes were capable of efficiently hydrogenating **S127a** in solvent-free conditions,^445^ in neat water,^446^ in ionic liquids,^447,448^ and in oligo(ethylene glycol)s through host–guest interactions.^449^ On the other hand, the AH of 2-aryl-substituted quinolines was more challenging, and only a few examples have been reported. The same group extended the use of these Ru(II)–diamine complexes and found that **C14h** was a highly active catalyst for the AH of 2-aryl quinolines, affording the corresponding **P127a** (R = aryl) in excellent yields and enantioselectivities (Scheme 85).^444^ Previously, Fan, Xu, and co-workers had already disclosed that air-stable and phosphine-free iridium complexes were also efficient catalysts for the highly enantioselective hydrogenation of quinoline derivatives.^450^

Y.-G. Zhou’s laboratory reported other important achievements in the field. In 2003, they pioneered the use of iridium complexes bearing axially chiral phosphines that, in combination with I_2_, were found to be highly active in the AH of 2-alkyl quinolines (Scheme 85).^451^ In particular, and when using **L17a**, **P127** were obtained in excellent yields and enantioselectivities (up to 96% ee). Using the same catalytic system, the substrate scope was further expanded to a wide range of 2-functionalized quinoline derivatives.^452−457^ More importantly, 2,3-dialkylquinolines were also efficiently hydrogenated using the same catalyst, although **P127b** were furnished from moderate to good enantioselectivities (up to 86% ee). Zhou’s group later reported the iridium-catalyzed AH of quinolines activated by Brønsted acids, thus avoiding catalyst activation using I_2_.^458^

The highly enantioselective and catalytic hydrogenation of 3-alkyl-2-arylquinolines remained a challenging task until 2019, when Hu and co-workers examined the use of structurally fine-tuned phosphine-phosphoramidite **L1**-type ligands (Scheme 85).^459^ Using **L1b** as a chiral ligand, a highly diastereo- and enantioselective iridium-catalyzed AH of unfunctionalized 3-alkyl-2-arylquinolines was disclosed. The transformation displayed broad functional group tolerance, thus furnishing a wide range of 2,3-disubstituted tetrahydroquinolines **P127b** in up to 96% ee and with perfect *cis*-diastereoselectivity. In contrast, the AH of 2,3-diaryl quinolines remains unsolved. In general, the AH of 2,3-disubstituted quinolines represents a more difficult task due to the requirement of diastereocontrol in the construction of two vicinal stereogenic centers. Nevertheless, the AH of functionalized 2,3-disubstituted quinolines with a phthaloyl^460,461^ or an ester^462^ group at the 3-position and an alkyl group at the 2-position of quinoline has been accomplished.

**Scheme 86:**
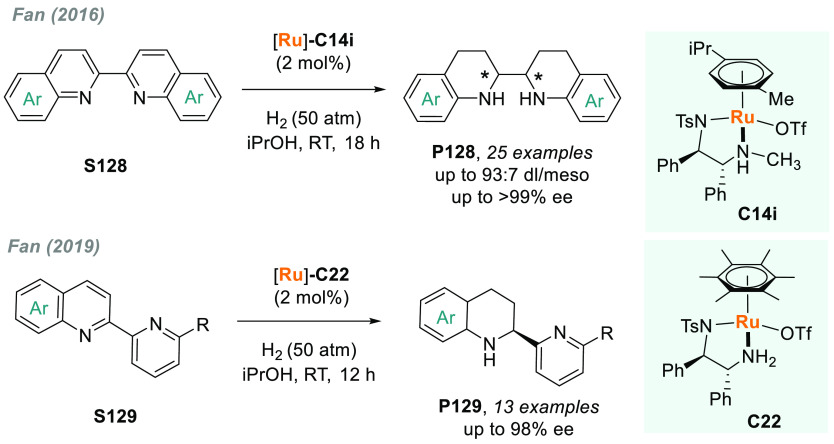
Ruthenium-Catalyzed AH of 2,2′-Bisquinoline Derivatives

The chemistry of chiral vicinal diamines and their derivatives has attracted a great deal of interest because they are key substructures in many biologically active compounds. In 2016, Fan and co-workers reported the highly enantioselective synthesis of vicinal diamines by direct AH of 2,2′-bisquinolines **S128** (Scheme 86).^463^ Using the Ru/diamine complex **C14i**, the reaction proceeded with very good diastereoselectivity while reaching an unprecedented level of enantioselectivity (>99% ee in the best cases). The resulting chiral vicinal diamines **P128** can be used as new chiral ligands. Similarly, the same group later described the use of Ru-complex **C22** for the AH of 2-(pyridine-2-yl)quinoline derivatives **S129** (Scheme 86).^118^ Based on the resulting chiral scaffolds **P129**, a small library of tunable chiral pyridine-aminophosphine ligands were readily prepared.

**Scheme 87:**
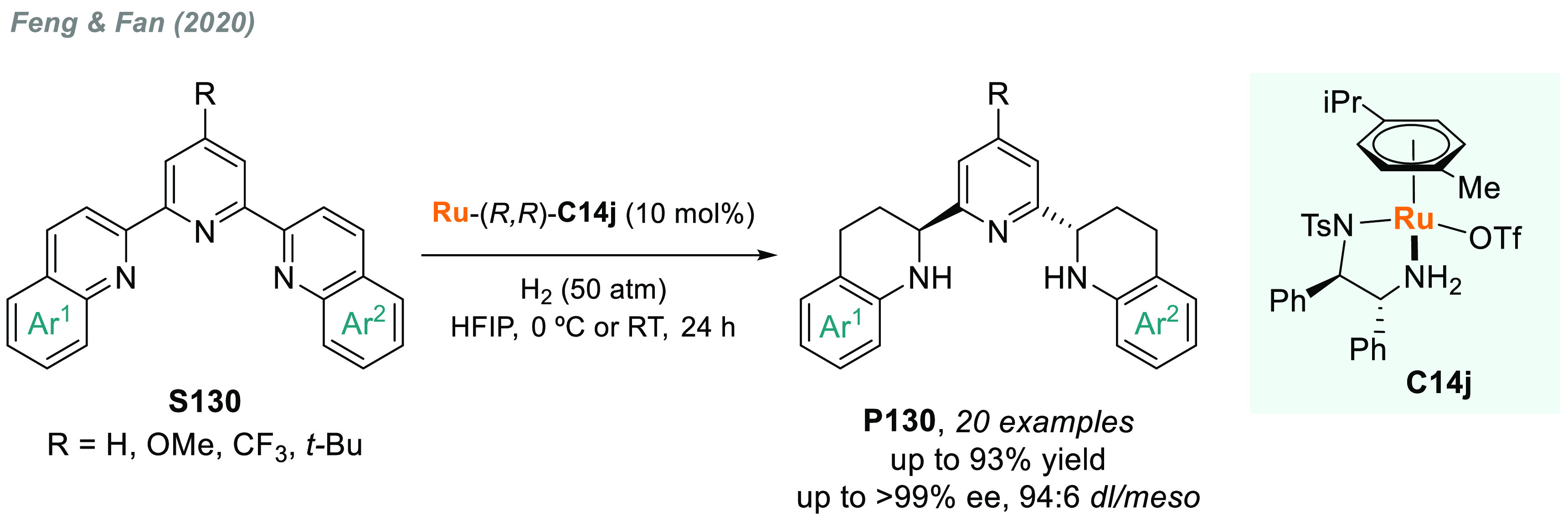
Ruthenium-Catalyzed AH of 2,6-Bis(quinolinyl) Pyridines (PyBQs)

In addition, the enantioselective hydrogenation of 2,6-bis(quinolinyl) pyridines (PyBQs) or terpyridine-type *N*-heteroarenes **S130** was successfully developed in 2020 by Fan’s group using Ru(diamine) complex **C14j** as catalyst (Scheme 87).^464^ The method provided partially reduced chiral pyridine-amine-type products **P130** in high yields with excellent diastereo- and enantioselectivity, which can serve as a new class of chiral nitrogen-donor ligands.

**Scheme 88:**
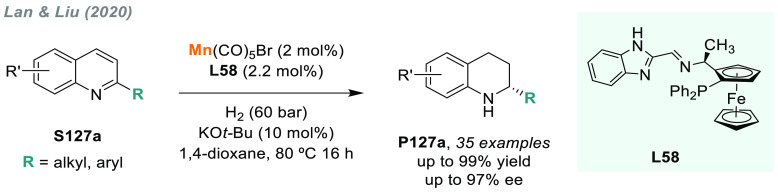
Mn-Catalyzed AH of Quinolines Enabled by π–π Interaction

The AH of quinolines using a non-noble metal was also accomplished by Lan, Liu, and co-workers in 2020. Using a chiral pincer manganese catalyst, the AH of quinolines **S127a** was achieved in high yields and enantioselectivities (up to 97% ee, Scheme 88).^465^ Interestingly, an effective π–π interaction between the C=N double bond and the imidazole ring of the ligand **L58** ensured precise regulation of the enantioselectivity. Undoubtedly, this represents an important precedent for the efficient AH of *N*-heteroaromatics without the need for precious metals.

Although the catalytic AH of quinolines is the most direct and reliable approach, Mashima, Ratovelomanana-Vidal, Ohsima, and co-workers reported the AH of quinolinium salts using cationic Ir(III) halide complexes with difluorphos (**L20**).^466^ This catalyst system successfully converted both 2-aryl- and 2-alkyl-quinolinium salts to the corresponding THQs with excellent enantioselectivities (up to 95% ee). The AH of quinolinium salts has also been applied as the key step for the synthesis of vabicaserin.^467^

**Scheme 89:**
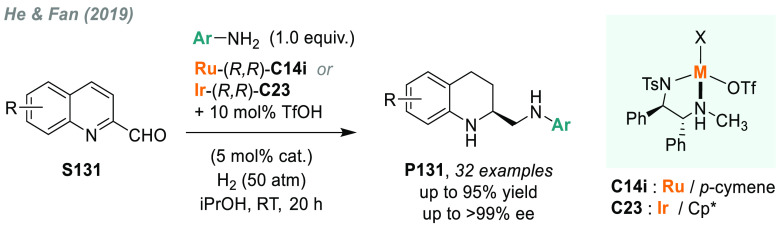
Consecutive Intermolecular Reductive Amination/AH

The asymmetric synthesis of THQs using tandem processes involving hydrogenation has been thoroughly explored in recent years. In 2019, Fan, He, and co-workers reported a novel strategy for the synthesis of chiral vicinal diamines based on a consecutive Ir- or Ru-catalyzed tandem intermolecular reductive amination/asymmetric hydrogenation.^468^ Using the appropriate catalyst (**C14i** or **C23**), 2-quinoline aldehydes (**S131**) and feedstock anilines were transformed into a broad range of sterically tunable chiral diamines **P131**, which were afforded in high yields with excellent enantioselectivity (Scheme 89). The usefulness and practicality of the method were exemplified by the transformation of **P131** into sterically hindered chiral *N*-heterocyclic carbene precursors.

**Scheme 90:**
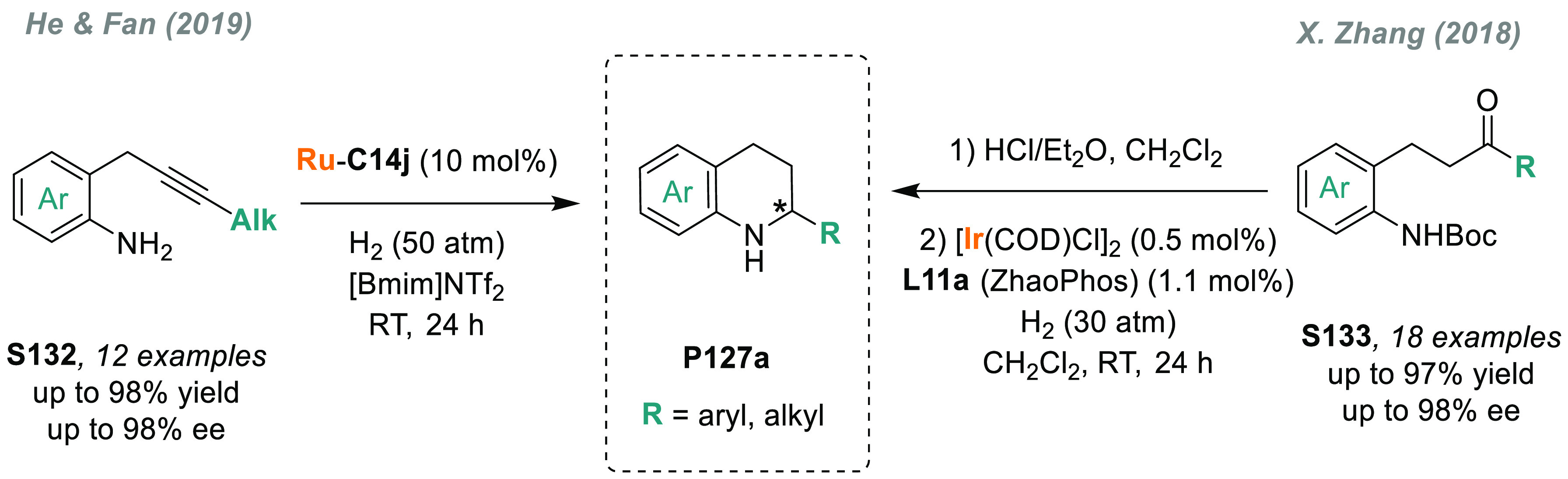
Asymmetric Synthesis of THQs through Sequential Processes

The same group developed a synthetic route to chiral THQs via sequential intramolecular hydroamination and ruthenium-catalyzed AH of anilino-alkynes **S132** (Scheme 90).^469^ Alternatively, X. Zhang and co-workers reported a one-pot process involving *N*-Boc deprotection/intramolecular asymmetric reductive amination using **S133** (Scheme 90).^470^ This latter methodology was also applied to the synthesis of tetrahydroisoquinolines^471^ as well as enantioenriched dibenz[c,e]azepines.^472^

### 6.2 Isoquinolines, Pyridines, and Pyridinium Salts

**Scheme 91:**
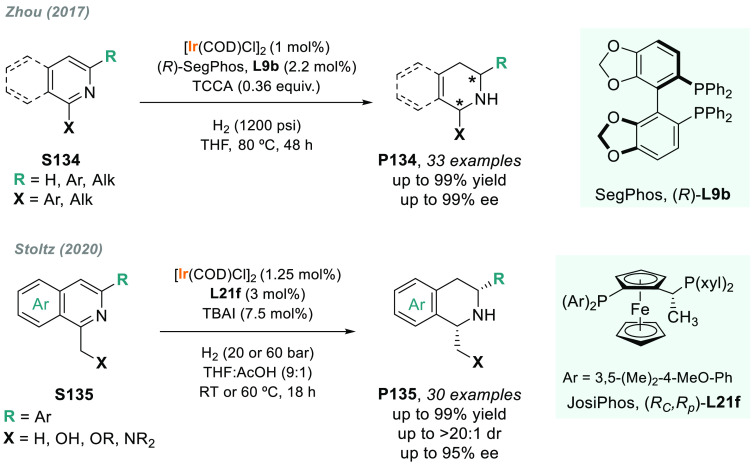
Iridium-Catalyzed AH of Isoquinolines and Pyridines

**Scheme 92:**
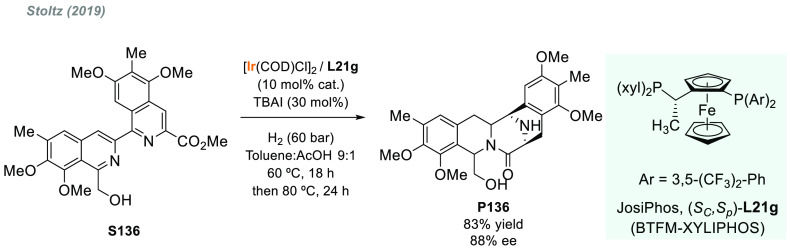
Enantioselective Hydrogenation as the Key Step for the Total Synthesis of (−)-Jorunnamycin A and (−)-Jorumycin

In contrast to quinolines, the transition metal-catalyzed AH of isoquinolines has remained significantly underdeveloped. The resulting products (1,2,3,4-tetrahydroisoquinolines, THIQs) have a stronger basicity and coordination ability, which can lead to a more facile catalyst deactivation. Typically, the strategy most widely used for the AH of substituted isoquinolines was substrate activation via isoquinolinium salts.^473−477^ However, the requirement of prior formation of the salt for activation was a considerable limitation. Furthermore, *in situ* and transient activation using chloroformates^478^ or halogenides^479,480^ has also been reported. An example of this approach was provided by Zhou’s group in 2017 (Scheme 91).^480^ By using Ir/(*R*)-**L9b** as catalyst, a variety of both isoquinolines and pyridines (**S134**) were hydrogenated in excellent yields and enantioselectivities. The reaction was promoted using halogenide trichloroisocyanuric acid (TCCA) as traceless activation reagent. Mechanistic studies indicated that hydrogen halide generated *in situ* acted as the substrate activator. Nevertheless, and although this method avoided the tedious steps of the introduction and removal the activation groups, the reaction conditions were harsh as high temperatures and H_2_ pressure were required. To overcome these limitations, Stoltz and co-workers recently reported a highly straightforward method for the iridium-catalyzed AH of 1,3-disubstituted isoquinolines **S135** under very mild conditions (Scheme 91).^481^ The reaction, which involves a commercially available ligand (**L21f**), proceeded in good yields with high levels of enantio- and diastereoselectivity. The key to the success of this approach was the introduction of a directing group at the C1 position that enabled hydrogenation to occur under mild reaction conditions. As a result, a wide variety of chiral THIQs **P135** were prepared in optically pure form, in a broad scope and with a very high tolerance of Lewis basic functionalities. Using a similar catalytic system, the same authors applied this hydrogenation reaction as the key step to the concise total synthesis of (−)-jorunnamycin A and (−)-jorumycin (Scheme 92), which were attained with high efficiency after 15 and 16 steps, respectively.^482^

**Scheme 93:**
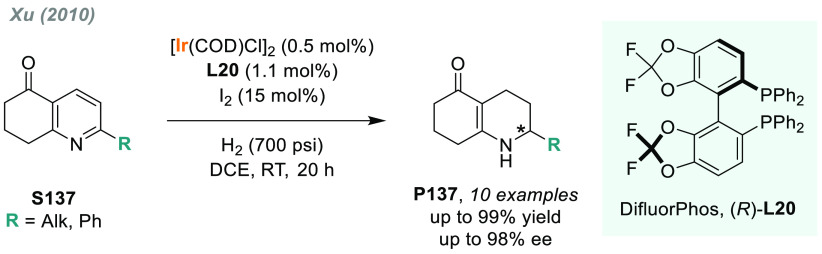
Iridium-Catalyzed AH of Trisubstituted Pyridines

Pyridines are also challenging substrates. Xu and co-workers reported the iridium-catalyzed AH of trisubstituted pyridine derivatives. They described that the iridium complex generated *in situ* from [Ir(COD)Cl]_2_, difluorPhos-**L20**, and I_2_ was an excellent catalyst for the AH of **S137**, affording the corresponding chiral amines **P137** in excellent yields and enantioselectivities (Scheme 93).^483^ The same group reported that P-Phos (**L16**) could also be used as a chiral ligand without diminishing the reactivity or enantioselectivity.^484^ These protocols, which could also be applied to the AH of quinolines, were more efficient than the previous one reported by Zhou’s group.^485^

**Scheme 94:**
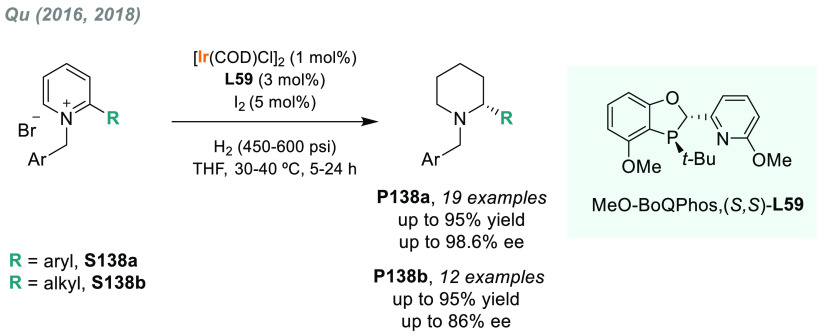
Iridium-Catalyzed AH of Pyridinium Salts

To facilitate the AH of pyridines, the activation of pyridine derivatives was first studied. In 2005, Legault and Charette demonstrated that chiral piperidine derivatives could be attained with high enantioselectivities (up to 90% ee) via the AH of *N*-acyliminopyridinium ylides using a Ir/P,N catalyst.^486^ Later, Andersson further developed this methodology with a screening of iridium catalysts with chiral *P*,*N*-ligands.^487^ In the search for an operationally simple protocol for large-scale synthesis, the *N*-benzylpyridinium salts emerged as the best method for pyridine activation. First described by Y.-G. Zhou’s group^488^ and applied by X. Zhang’s^489^ and Lefort’s laboratories,^490^ this method provided a better approach in terms of safety and applicability. Using their catalytic systems, the subsequent iridium-catalyzed AH of these *N*-benzylpyridinium salts furnished a wide variety of 2-arylpyridines in both high yields and enantioselectivities, but with limited application to 2-alkylpyridines. Qu et al. then reported the use of a new rigid P-stereogenic *P*,*N*-ligand **L59** for the iridium-catalyzed AH of **S138** (Scheme 94).^491^ Chiral α-(hetero)aryl piperidines **P138a** were afforded in high levels of enantioselectivity. Using the same ligand, chiral α-alkyl piperidines **P138b** were also prepared but with lower enantiocontrol.^492^ This catalytic system was also applied in the asymmetric construction of the indenopiperidine core of an 11β-HSD-1 inhibitor.^493^

**Scheme 95:**
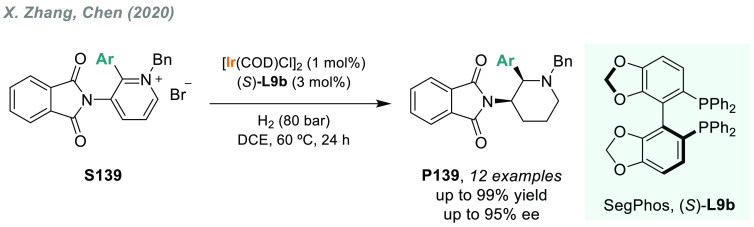
Iridium-Catalyzed AH of 2-Aryl-3-phthalimidopyridinium Salts

In addition, Mashima and Zhou’s laboratories independently reported another strategy for the AH of Brønsted acid-activated multisubstituted pyridines using enantiopure binuclear iridium complexes, thus affording the corresponding chiral piperidines in high diastereo- and enantioselectivities (up to 90% ee).^494,495^ Later, Mashima expanded this methodology to the AH of 2-aryl-3-amidopyridinium salts, delivering the corresponding chiral piperidines with high diastereoselectivities and good enantioselectivities (up to 86% ee).^496^ These piperidines containing two contiguous centers as structural moieties are present in many neurokinin-1 (NK1) receptor antagonist derivatives, such as (+)-CP-99994 and Vofopitant. Due to the importance of these structural units, X. Zhang and co-workers recently described the AH of 2-aryl-3-phthalimidopyridinium salts **S139** driven by the Ir/SegPhos (*S*)-**L9b** catalytic system (Scheme 95).^497^

Furthermore, the AH of 3-substituted pyridinium salts was developed by Lefort and co-workers using a Rh-JosiPhos catalyst with ee values up to 90%.^498^ Working with similar substrates, Y.-G. Zhou’s laboratory recently reported a highly enantioselective hydrogenation of 3-hydroxypyridinium salts using Ir/f-binaphane (**L7**) followed by sequential Swern oxidation, thus furnishing chiral 6-substituted piperidin-3-ones in excellent enantioselectivities (up to 95% ee).^499^ On the other hand, in 2018, Peters and co-workers reported an alternative synthesis of piperidines from isoxazolinones by Pd/Ir relay catalytic hydrogenation of the initially formed 3,4-dihydropyridines.^500^

### 6.3 Quinoxalines and Pyrazines

**Scheme 96:**
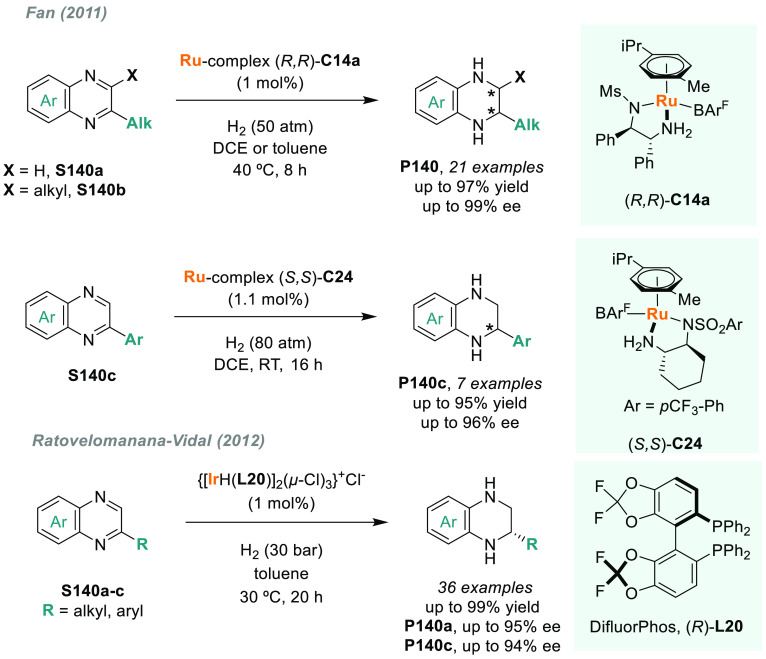
Ruthenium-Catalyzed AH of Quinoxalines

The 1,2,3,4-tetrahydroquinoxaline ring system is of great interest as a key structural unit in many therapeutically active compounds. In 2011, Fan’s group described that the family of half-sandwich Ru-diamine complexes, which had already been used for the AH of quinolines, were excellent catalysts for the AH of other heteroaromatic compounds such as quinoxalines. In particular, catalyst **C14a** was used for the enantioselective hydrogenation of 2-alkyl and 2,3-dialkyl quinoxalines **S140** with up to 99% ee (Scheme 96).^501^ Of note, the bulky and weakly coordinating counteranion (BAr^F^) was found to be critical for the high enantioselectivity and/or diastereoselectivity. On the other hand, and using **C24**, 2-aryl quinoxalines **S140c** were also hydrogenated in excellent ee values (up to 96% ee, Scheme 96). Later, in 2013, Ohkuma and co-workers reported an alternative protocol for the enantioselective hydrogenation of 2-alkyl quinoxalines using chiral ruthenabicylic complexes.^129^

Although this work is the most successful precedent in the field, iridium catalysts had also found use in the AH of quinoxalines. In this regard, Chan, Xu, Fan, and co-workers described a highly efficient AH of quinoxalines using Ir/H_8_-binapo at low catalyst loading.^502^ Later, Ratovelamana-Vidal and co-workers described a general AH of a wide range of 2-alkyl- and 2-aryl-substituted quinoxaline derivatives using a cationic dinuclear triply chloride-bridged Ir(III) complex bearing **L20** as a chiral ligand.^503,504^ Of note, the efficiency of the catalytic system was demonstrated through a broad scope with excellent enantioselectivities (up to 95% ee, Scheme 96).

Mashima and co-workers showed that the use of amines as additives had a positive effect for the iridium-catalyzed AH of quinoxalines.^505^ In particular, they found that *N*-methyl-*p*-anisidine (MPA) was the best amine additive for achieving high enantioselectivity. More recently, Nagorny applied new chiral SPIROL-based phosphinite ligands for the iridium-catalyzed AH of heterocycles, including quinoxalines, in good to excellent enantioselectivities.^506^

While 2-alkyl- and 2-aryl-substituted quinoxalines have been hydrogenated at useful levels of enantioselectivity, the AH of other derivatives such as quinoxaline-2-carboxylates is still underdeveloped, although the resulting chiral cyclic amino acids are highly valuable. To the best of our knowledge, there is only one example in the literature, affording the corresponding chiral amino acid in moderate enantioselectivity (74% ee).^507^

**Scheme 97:**
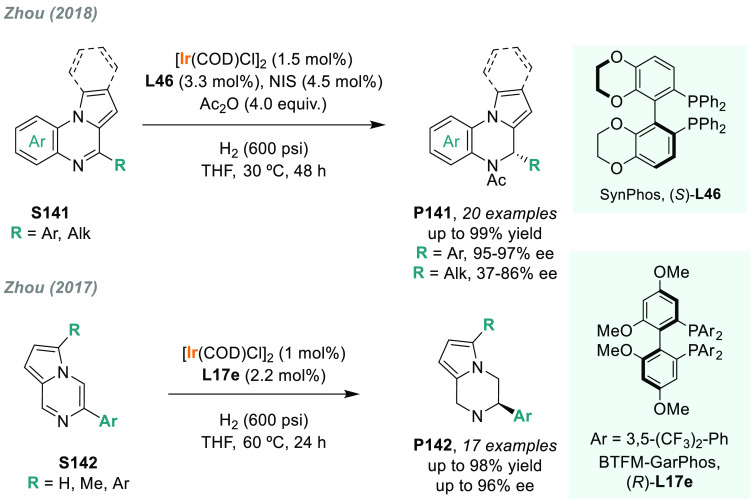
Iridium-Catalyzed AH of Pyrrolo/indolo[1,2-*a*]quinoxalines and of Pyrrolo[1,2-*a*]pyrazines

Iridium catalysis was also applied to the AH of pyrrolo/indolo[1,2-*a*]quinoxalines (Scheme 97). In 2018, Zhou’s laboratory described the highly enantioselective hydrogenation of **S141** with Ir/Synphos-**L46**, providing facile access to chiral **P141** with up to 97% ee.^508^ However, the addition of acetic anhydride was pivotal for suppressing the rearomatization of hydrogenation products. The system was also applied to the AH of phenanthridines (see section 6.5). The same laboratory studied the iridium-catalyzed AH of pyrrolo[1,2-*a*]pyrazines **S142** (Scheme 97).^509^ In this case, the preparation of the corresponding salt or the addition of a *N*-protecting group was not required. The reaction was performed under very mild conditions, and it exhibited excellent activity and enantioselectivity when using **L17e** as a chiral ligand. The group previously reported the enantioselective synthesis of **P142** via the iridium-catalyzed AH of pyrrolo[1,2-*a*]pyrazinium salts, which were prepared after substrate activation using alkyl halides.^510^

**Scheme 98:**
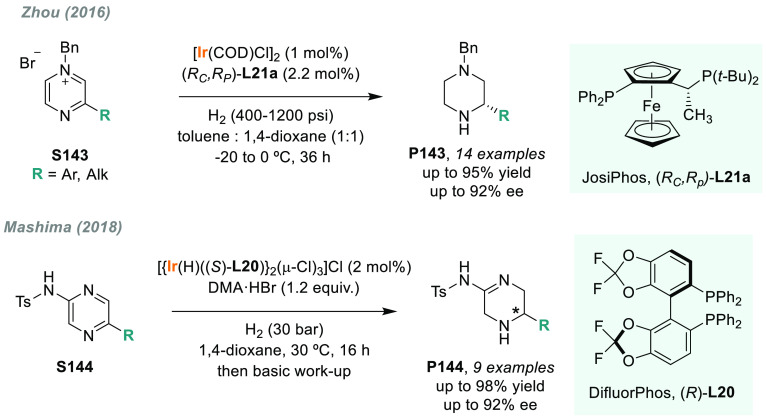
Iridium-Catalyzed AH of Pyrazinium Salts

In 2016, Zhou’s laboratory reported a facile method for the synthesis of chiral piperazines **P143** through iridium-catalyzed AH of 3-substituted pyrazinium salts **S143** using JosiPhos-type ligand **L21a** (Scheme 98).^511^ The system showed broad substrate scope with good to excellent selectivity (up to 92% ee). Furthermore, 2,3- and 3,5-disubstituted pyrazinium salts were also hydrogenated with high yields and enantioselectivities when using the appropriate chiral ligand. To demonstrate the applicability of the methodology, Vestipitant, a potent and selective NK1 receptor antagonist, was prepared in just two steps.

In a similar approach, Mashima and co-workers reported the iridium-catalyzed enantioselective hydrogenation of tosylamido-substituted pyrazines **S144**. The addition of *N,N-*dimethylanilinium bromide (DMA·HBr) enhanced the catalytic activity of the iridium complexes, as well as the enantioselectivity (Scheme 98).^512^ Chiral tetrahydropyrazines with an amidine skeleton (**P144**) were obtained with good to excellent enantioselectivities (up to 92% ee) using dinuclear triply chloro-bridge Ir(III) complexes bearing chiral difluorPhos (**L20**). The resulting tetrahydropyrazines **P144** are versatile precursors for the preparation of chiral piperazine derivatives without loss of enantioselectivity.

### 6.4 Indoles

**Scheme 99:**
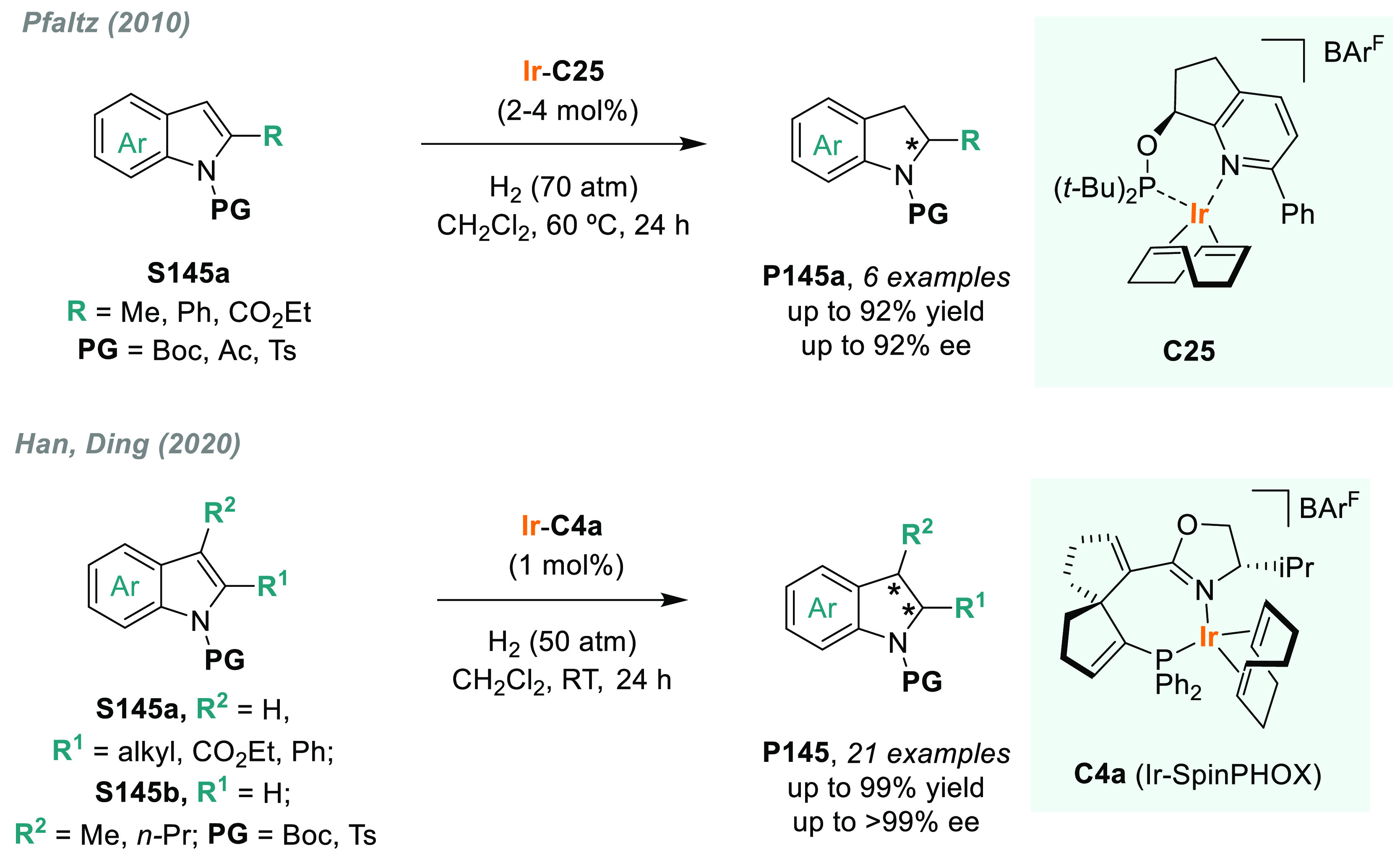
Iridium-Catalyzed AH of *N*-Protected Indoles

Iridium chiral complexes were also applied to the AH of *N*-protected indoles to obtain chiral indolines, which are common structures occurring in alkaloids and other natural or synthetic products with biological activity.^513^ Pfaltz pioneered the use of cationic Ir complexes derived from PHOX or other chiral *P*,*N*-ligands in the AH of 2-substituted *N*-protected indoles **S145a** (Scheme 99).^514^ In particular, **C25** gave the highest enantioselectivities, and the results obtained demonstrate that the protecting group (*N*-Boc, *N*-acetyl, or *N*-tosyl) influences both reactivity and enantiomeric excess. Moreover, various examples of 3-substituted indoles were also hydrogenated, using the right combination of catalyst and protecting group. On the other hand, Han, Ding, and co-workers recently reported that Ir/SpinPHOX **C4a** is an efficient catalyst for the AH of both 2- and 3-substituted *N*-protected indoles (Scheme 99).^515^ The corresponding chiral indolines were afforded in excellent yields and enantioselectivities (>99% ee in most cases).

**Scheme 100:**
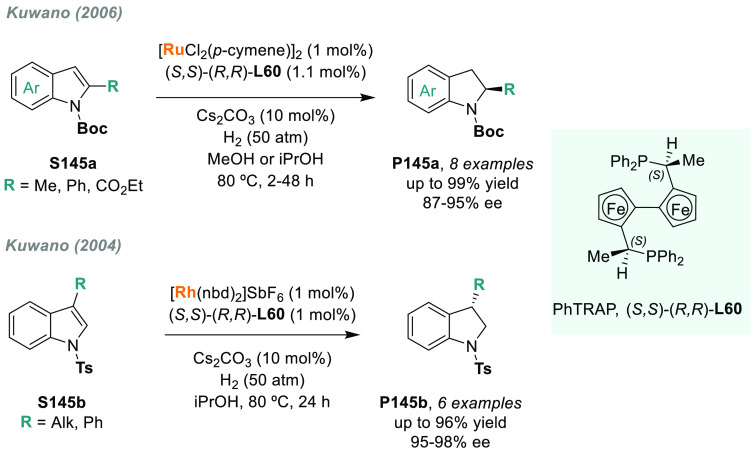
Metal-Catalyzed AH of *N*-Protected Indoles Using PhTRAP

Prior to these iridium complexes, the only suitable catalytic systems known for the AH of *N*-protected indoles were Ru and Rh complexes bearing the *trans*-chelating chiral diphosphine (*S,S*)-(*R,R*)-PhTRAP ligand (**L60**), first reported by Kuwano’s group.^516^ In their work, a wide range of *N*-protected indoles were hydrogenated with high efficiency, thus providing the corresponding chiral indolines in excellent yields and enantioselectivities.^517^ For example, the ruthenium-catalyzed AH of *N*-Boc 2-substituted (**S145a**) and *N*-tosyl 3-substituted indoles (**S145b**) furnished the corresponding indolines (**P145)** in excellent yields using **L60** (Scheme 100).^518,519^ 2-Substituted indole esters were also hydrogenated by the groups of Minaard^520^ and Agbossou-Niedercorn^521^ using Rh complexes bearing PinPhos or WalPhos as catalysts, although only moderate enantioselectivities were achieved.

**Scheme 101:**
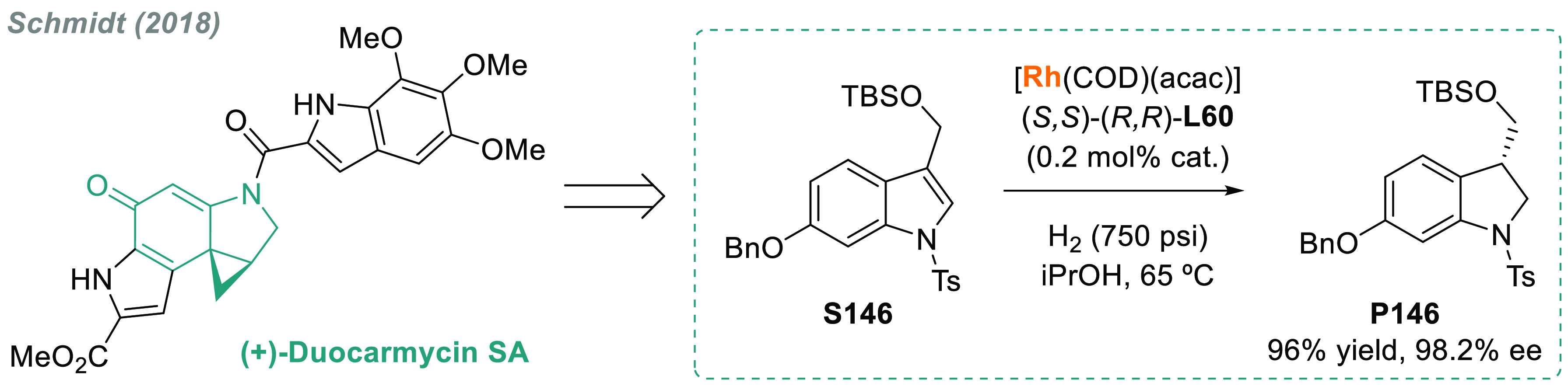
Synthesis of (+)-Duocarmycin SA via Rhodium-Catalyzed AH

The methodology developed by Kuwano and co-workers allows the preparation of a wide range of optically active indolines with a chiral center at the 3-position. A team at Bristol-Myers Squibb recently used it in the enantioselective total synthesis of (+)-Duocarmycin SA, which is a potent antitumor antibiotic (Scheme 101).^522^ The key tricyclic core was constructed through a highly enantioselective hydrogenation of indole **S146** using **L60**.

The progress achieved in the AH of *N*-protected indoles was in sharp contrast to the AH of simple unprotected indoles, which remain a challenge in organic synthesis. However, significant advances were made in this field in the last ten years.

**Scheme 102:**
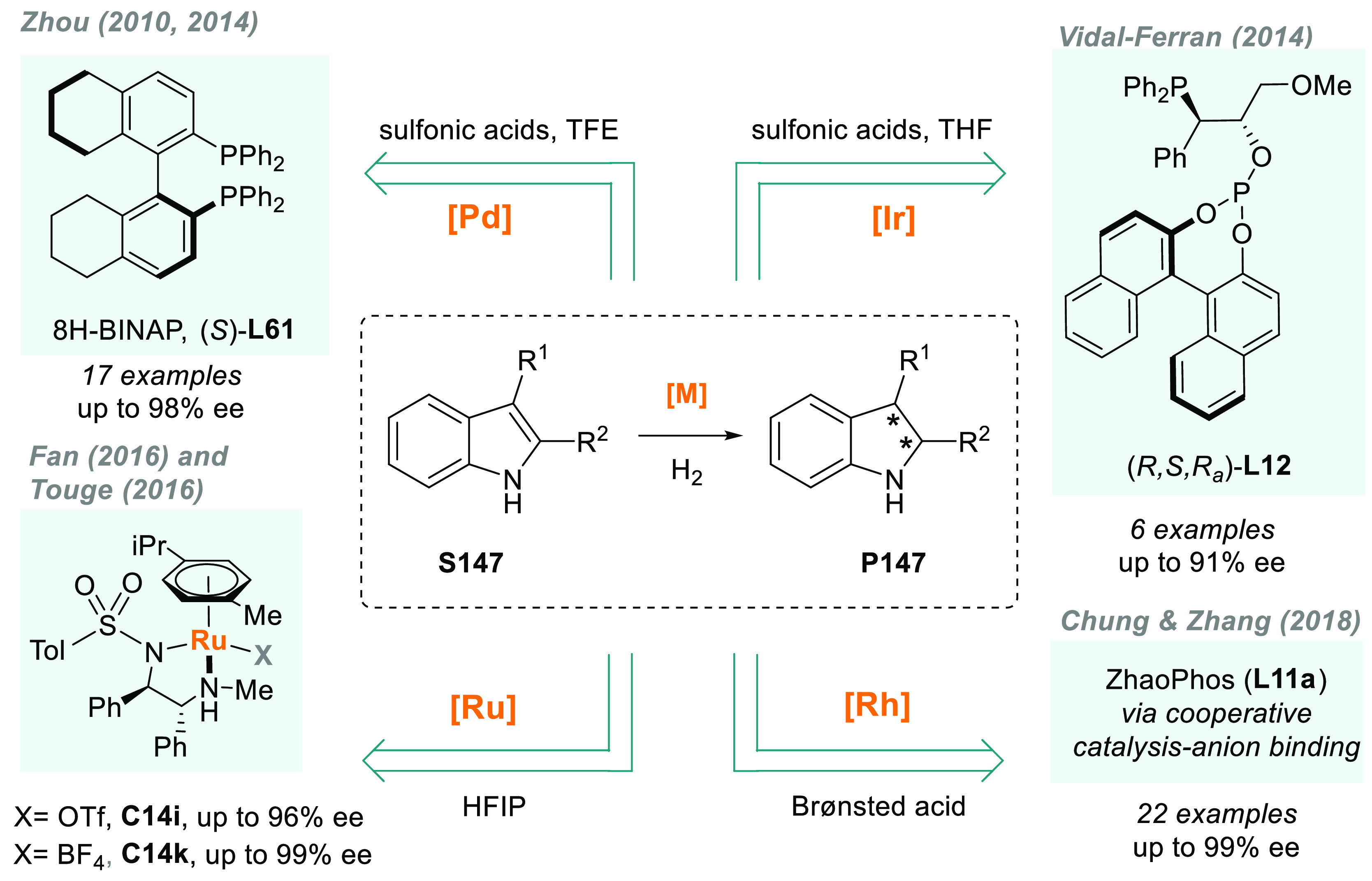
Catalytic Strategies toward the AH of Unprotected Indoles

In 2010, Y.-G. Zhou, X. Zhang, and co-workers developed the first highly enantioselective hydrogenation of simple indoles using Pd/(*R*)-H_8_-BINAP (**L61**) with a Brønsted acid (l-camphorsulfonic acid, l-CSA) as an activator and TFE as solvent (Scheme 102).^523^ This methodology provided an efficient route to make chiral indolines from unprotected indoles in excellent enantioselectivities (up to 96% ee). In 2014, Zhou’s laboratory reported an extensive substrate scope, including 2-substituted and 2,3-disubstituted indoles (**S147**).^524^ Using the same reaction conditions as the previous work, chiral indolines were prepared in up to 98% ee (Scheme 102). The main drawback of this approach was the low activity and/or enantioselectivity of the 2-aryl-substituted indoles. Interestingly, a mechanistic study using DFT calculations revealed that the hydrogenation occurs through a stepwise, ionic, and outer-sphere mechanism. As an alternative approach, W. Zhang reported that Pd/(*S*)-C_10_-BridgePhos is a highly efficient catalyst for the AH of 2-, 3-, and 2,3-substituted unprotected indoles.^525^

Sulfonic acids were also used as Brønsted acids in the iridium-catalyzed AH of **S147**, reported by Vidal-Ferran and co-workers, using a P-OP ligand **L12** and THF as solvent (Scheme 102).^526^ Enantiomerically enriched indolines **P147** were attained in up to 91% ee. The Brønsted acid (*rac*-CSA), which could be reused by addition of heterogeneous additives, activates the indole ring by breaking its aromaticity. Interestingly, the reaction proceeded by a stepwise process: Brønsted acid-mediated C=C isomerization, thus generating the corresponding iminium ion, followed by asymmetric hydrogenation.

Chung and X. Zhang also envisioned a similar strategy. They developed an efficient method to obtain optically pure indolines using a Rh/ZhaoPhos-**L11a** complex (Scheme 102).^527^ Various 2-substituted and 2,3-disubstituted indoles **S147** were hydrogenated with high enantioselectivities. By employing HCl as Brønsted acid, an active iminium ion intermediate was formed and reduced. The thiourea anion binding of **L11a** proved crucial to achieve high enantioselectivity and reactivity.

Fan and co-workers also studied the AH of unprotected indoles using half-sandwich Ru–diamine complexes. In particular, when using **C14i** with a triflate as counteranion, indolines **P147** were afforded in up to 96% ee (Scheme 102).^114^ Of note, the reaction was performed under very mild conditions at room temperature and using HFIP as solvent, which significantly influenced catalytic performance. Excellent enantio- and diastereoselectivities were obtained for a wide range of indole derivatives. Simultaneously, Touge and Arai and co-workers described that a similar Ru complex with a tetrafluoroborate as counteranion (**C14k**) also gave excellent yields and enantioselectivities in very mild conditions (Scheme 102).^528^

**Scheme 103:**
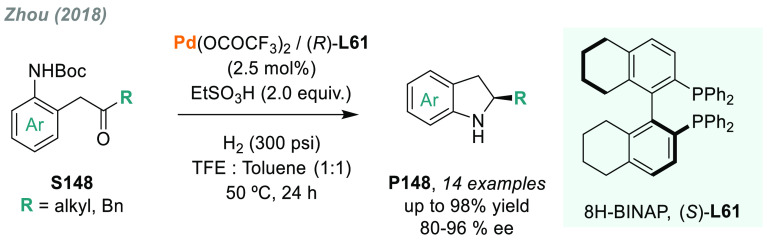
One-Pot Synthesis of Chiral Indolines via Palladium-Catalyzed AH

Subsequently, other related transformations using Pd catalysis emerged, including dehydration-triggered AH^529^ and consecutive Brønsted acid/Pd-complex-promoted tandem reactions.^530^ Moreover, 3-(toluenesulfonamidoalkyl)-indoles were synthesized and hydrogenated to form chiral indolines.^531^ Of note, Y.-G. Zhou’s group recently reported a facile synthesis of chiral indolines through the AH of *in situ* generated indoles.^532^ The reaction was developed through an intramolecular condensation, deprotection, and palladium-catalyzed AH using **L61** in a one-pot process (Scheme 103). Chiral 2-alkyl-substituted indolines **P148** were furnished in excellent yields and enantioselectivities (up to 96% ee).

### 6.5 Other Heteroaromatic Compounds

**Scheme 104:**
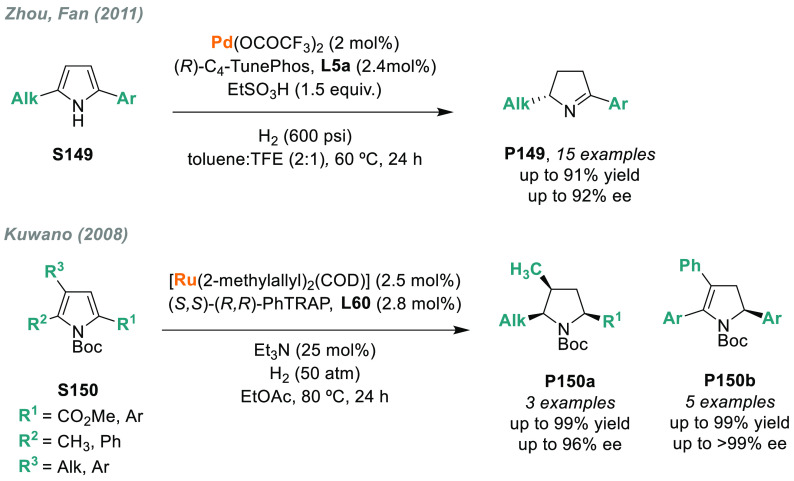
Metal-Catalyzed AH of Pyrroles

In 2011, Zhou, Fan, and co-workers disclosed the first AH of simple unprotected pyrroles. In their work, the Brønsted acid-activation mode was applied to the partial AH of pyrroles **S149** (Scheme 104).^533^ Using sulfonic acid as activator, a highly enantioselective palladium-catalyzed partial hydrogenation using C_4_-TunePhos (**L5a**) was reported, providing chiral 2,5-disubstituted 1-pyrrolines **P149** with up to 92% ee. Previously, Kuwano’s group applied the PhTRAP ligand **L60** to the ruthenium-catalyzed AH of *N*-Boc protected pyrroles (Scheme 104).^534^ Using this catalytic system, pyrroles **S150** were hydrogenated with high ee’s to give chiral pyrrolidines **P150a** or 4,5-dihydropyrroles **P150b**.

**Scheme 105:**
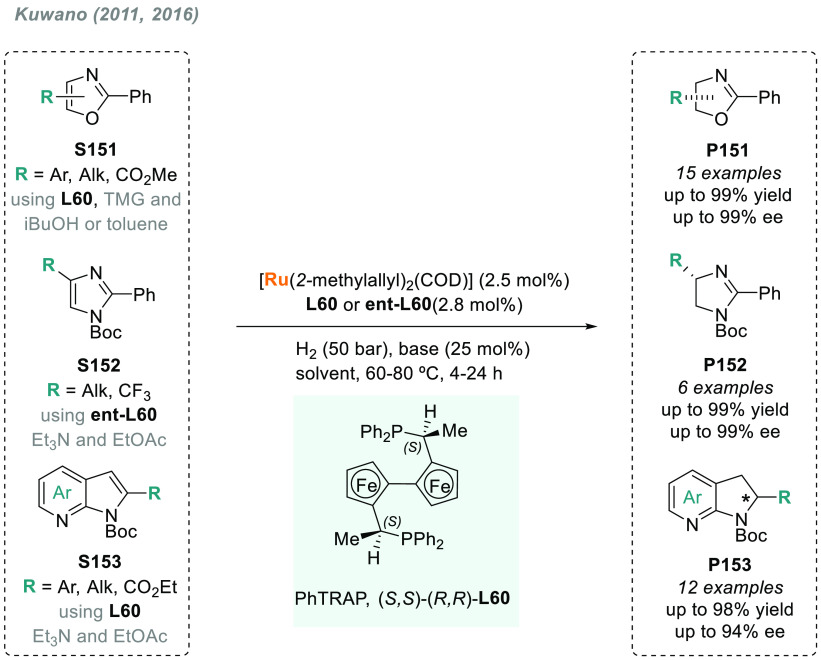
Ruthenium-Catalyzed AH of Oxazoles, Imidazoles, and Azaindoles Using PhTRAP

The Ru/**L60** system was further exploited by Kuwano’s group for the AH of other single-ring heteroarenes. Using this catalytic system, oxazoles **S151** and *N*-Boc imidazoles **S152** were hydrogenated into the corresponding chiral oxazolines **P151** and imidazolines **P152**, respectively (up to 99% ee, Scheme 105).^535^ These hydrogenation products are highly valuable synthetic intermediates, as they can be easily converted to 1,2-diamines or β-amido alcohols without loss of enantiopurity. On the other hand, fused aromatic rings consisting of two (or more) heteroarenes are challenging substrates due to the need to control chemoselectivity. Pyrido-fused pyrroles, namely azaindoles, are an important class of this type of substrate. In 2016, Kuwano and co-workers reported the chemo- and enantioselective reduction of 7-azaindoles **S153** using Ru/**L60** as catalyst (Scheme 105).^536^ The reaction occurred exclusively on the five-membered ring, thus furnishing the corresponding azaindolines **P153** with up to 94% ee. Furthermore, the catalyst was also highly active for the AH of 6-, 5-, and 4-azaindoles.

**Scheme 106:**
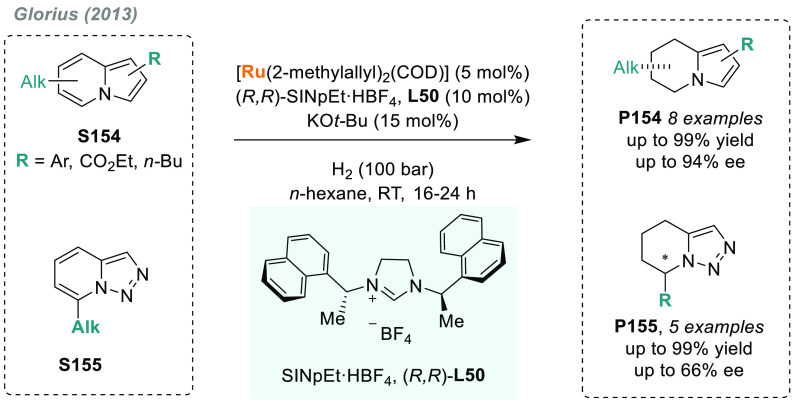
Ruthenium-Catalyzed AH of Indolizines and 1,2,3-Triazolopyridines

Indolizines are another class of ring-fused heteroaromatic compounds. However, their selective reduction is a difficult task due to the nitrogen atom of the bridgehead position. In fact, the efficient AH of indolizines had not been accomplished until 2013, when Glorius and co-workers described the Ru-catalyzed AH of indolizines **S154** using the chiral NHC-ligand **L50** (Scheme 106).^537^ The corresponding hydrogenated products, indolizidines **P154**, were afforded in high yields and enantioselectivities, and the applicability of such a methodology was exemplified by the synthesis of unnatural (−)-monomorine in only two steps. In addition, the catalyst was also used for the high-yielding and completely regioselective AH of 1,2,3-triazolo-pyridines **S155**, albeit in low to moderate enantioselectivity (Scheme 106). Later, the same group used this catalytic system for the enantioselective hydrogenation of imidazo[1,2-*a*]pyridines, with enantiomeric ratios of up to 98:2.^538^ Very recently, Fan and co-workers have also reported the synthesis of benzo-fused indolizidines and quinolizidines via tandem AH/reductive amination using a Ru-DPEN catalyst (**C14j**).^539^

**Scheme 107:**
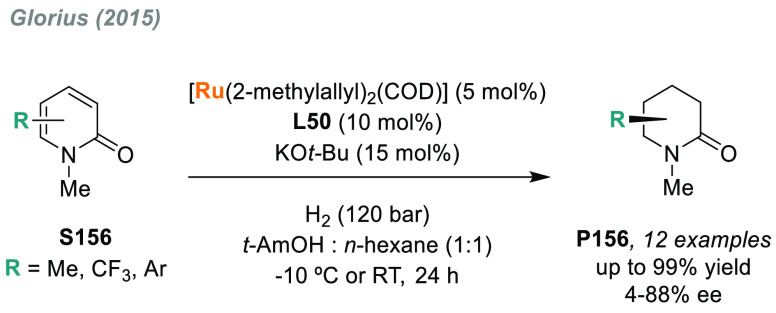
Ruthenium-Catalyzed AH of 2-Pyridones

In 2015, Glorius and co-workers applied the same catalyst Ru/**L50** for the AH of 2-pyridones **S156** into the corresponding enantioenriched 2-piperidones **P156** (Scheme 107).^540^ However, and even though this was the first related example using a homogeneous catalytic system, the method suffered from low enantioselectivities, and therefore there is still plenty of room for improvement.

**Scheme 108:**
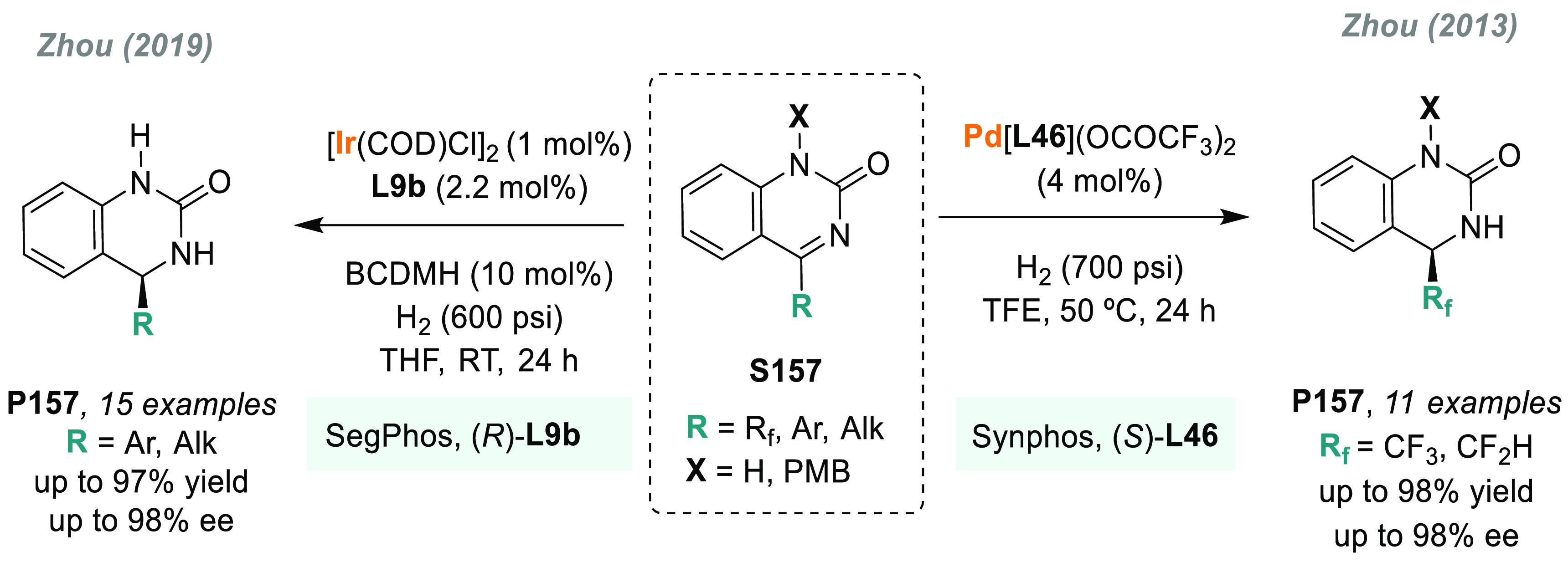
Enantioselective Hydrogenation of Quinazolinones Using Pd or Ir Catalysts

The enantioselective hydrogenation of quinozalinones was achieved by Zhou’s laboratory, using Pd or Ir catalysis (Scheme 108). First, the group disclosed that Pd/SynPhos [(*S*)-**L46**] was an excellent catalyst for the AH of fluorinated quinazolinones (94–98% ee).^541^ Later, the substrate scope was expanded to other substituted quinazolinones using iridium catalysis (with **L9b**), thus allowing the preparation of chiral dihydroquinazolinones **P157** in excellent yields and enantioselectivities (Scheme 108).^542^

**Scheme 109:**
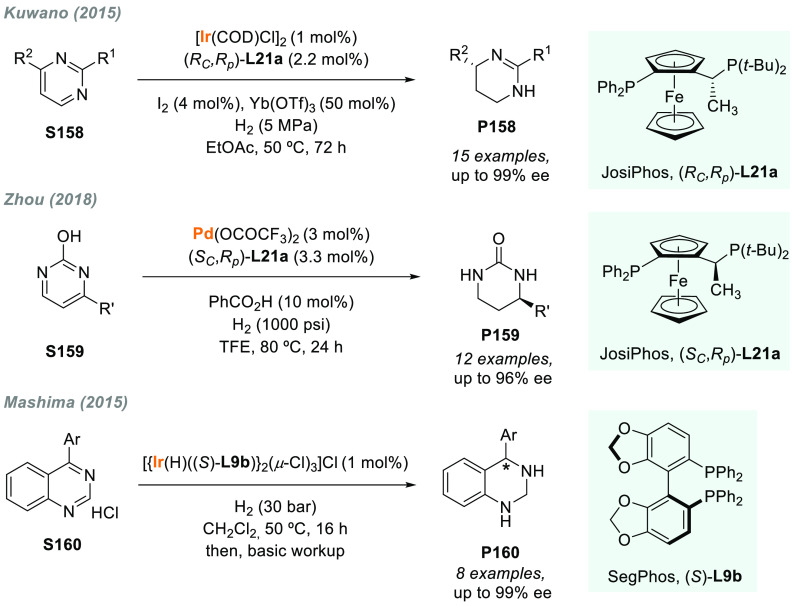
AH of Pyrimidines

The AH of pyrimidines was first reported by Kuwano and co-workers in 2015.^543^ Using an Ir/**L21a** as catalyst, various 2,4-disubstituted pyrimidines **S158** were converted into chiral 1,4,5,6-tetrahydropyrimidines **P158** in high yield (Scheme 109). Interestingly, lanthanide triflate was used as additive for achieving high enantiocontrol, as well as for activating the heteroarene substrate. Similarly, the AH of 2-hydroxypyrimidines **S159** was reported by Zhou’s group (Scheme 109).^544^ Taking advantage of the lactame-lactime tautomerism of the 2-hydroxypyrimidine, the authors developed two distinct catalytic systems for the efficient preparation of cyclic ureas **P159**, which are highly valuable structural motifs in many pharmacophores and other biologically active compounds. In particular, they reported an efficient Brønsted acid-activated palladium-catalyzed AH using chiral ligand **L21a**. By slightly modifying the catalytic system, di- and trisubstituted 2-hydroxypyrimidines were also hydrogenated in good yields. Alternatively, the same laboratory developed the same transformation using iridium catalysis with **L7**, in which the Brønsted acid was generated *in situ*.^545^ On the other hand, the AH of quinazolinium salts **S160** was reported by Mashima and co-workers (Scheme 109).^546^ By using halide-bridged dinuclear iridium complexes bearing SegPhos ((*S*)-**L9b**) as ligand, the corresponding 1,2,3,4-tetrahydroquinazolines **P160** were achieved in high enantiomeric excess along with some dihydroquinazolines.

**Scheme 110:**
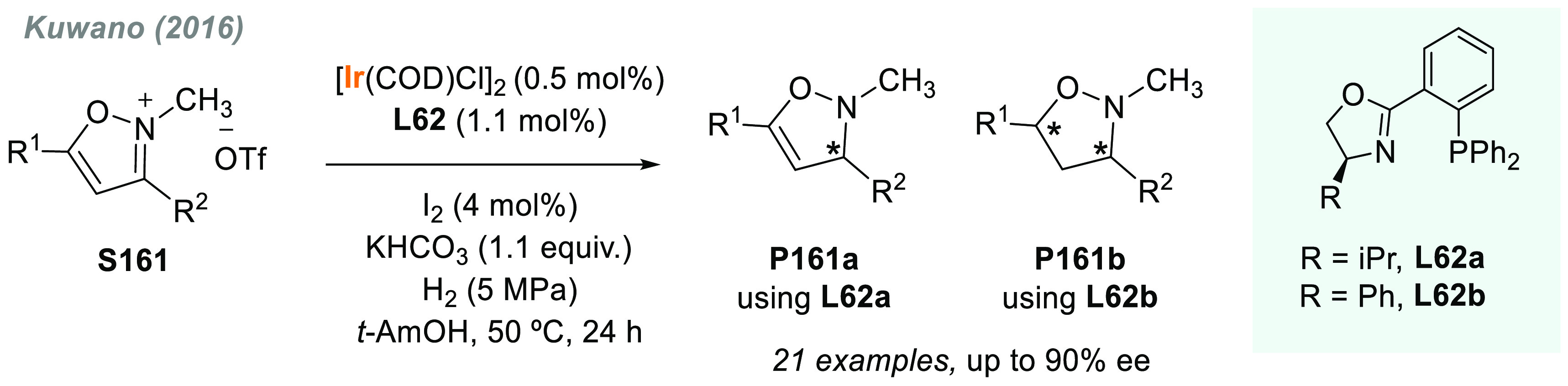
AH of Isoxazolium Salts

Iridium catalysis was also used for the efficient AH of isoxazolium salts (Scheme 110). Kuwano’s laboratory reported that iridium complexes bearing phosphino-oxazoline **L62**-type ligands were highly active catalysts for the AH of isoxazolium triflates **S161**.^547^ Interestingly, by fine-tuning the oxazoline substituent, isoxazolines **P161a** or isoxazolidines **P161b** were selectively obtained in good to high enantioselectivity.

Dihydrophenanthridines (DHPDs, **P162**) are important structural units in natural products and biologically active molecules. In addition, 9,10-dihydrophenanthridine proved to be a regenerable biomimetic hydrogen source, as it was designed as a NAD(P)H analog by Zhou.^548^

**Scheme 111:**
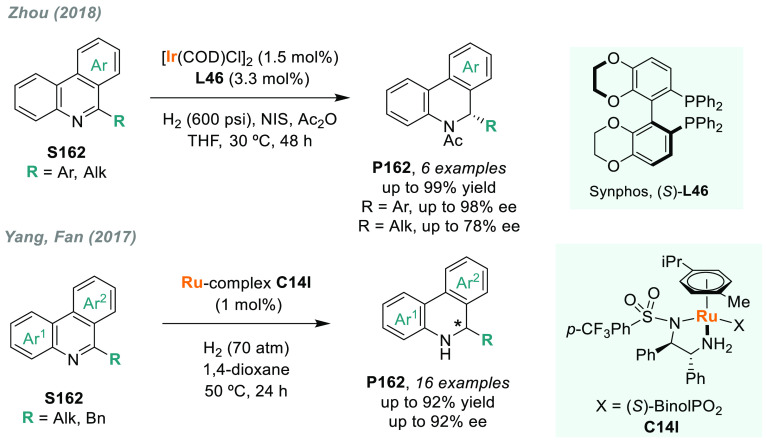
Metal-Catalyzed AH of Phenanthridines

However, until 2017, the AH of substituted phenanthridines had not been documented. Phenanthridines are heteroarenes formed by three fused aromatic rings. Their hydrogenation is difficult due to the possible dehydrogenative aromatization of the reduced products. Moreover, the strong coordinative nitrogen atom can easily poison the metal catalyst. At that point, Zhou’s laboratory reported a highly enantioselective iridium-catalyzed AH of phenanthridines **S162** through *in situ* protection of the reduced products with acetic anhydride to inhibit rearomatization (Scheme 111).^508^ This approach was also used for pyrrolo/indolo[1,2-*a*]quinoxalines, as previously stated (see Scheme 97). Aryl substituents were well-tolerated, and the corresponding DHPDs **P162** were attained with excellent yields and enantioselectivities. For alkyl substituents, the level of enantioselectivity was only moderate. Nevertheless, alkyl-substituted phenanthridines **S162** had previously been hydrogenated by Yang, Fan, and co-workers in a highly efficient manner (Scheme 111).^549^ By using a chiral cationic Ru-diamine complex **C14l**, the corresponding enantioenriched **P162** were obtained. Interestingly, the counteranion was found to be critical for attaining high enantioselectivities (up to 92% ee).

**Scheme 112:**
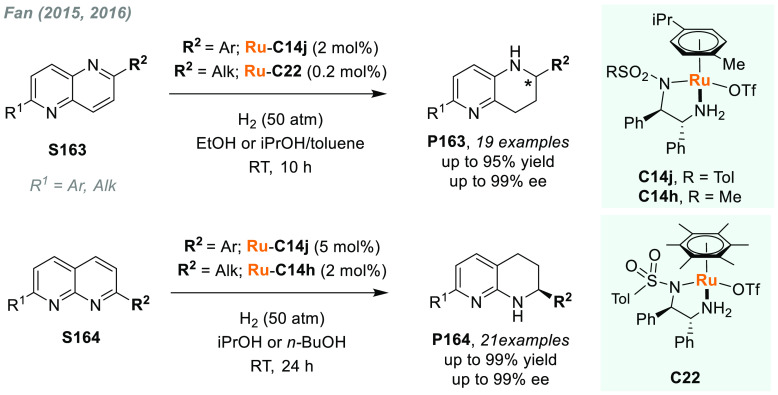
Ruthenium-Catalyzed AH of 1,5- and 1,8-Naphthyridines

Fan and co-workers further exploited the use of chiral cationic Ru-diamine complexes to the AH of 1,5- and 1,8-naphthyridines (Scheme 112).^550,551^ By using the appropriate Ru catalyst, chiral amines containing the 1,2,3,4-tetrahydronaphthyridine ring were afforded in excellent yields and enantioselectivities. In fact, these chiral heterocycles have been used as rigid chelating diamine ligands for asymmetric synthesis and can be found in many biologically active compounds. Previously, in 2013, the same authors reported the highly enantioselective ruthenium-catalyzed AH of 1,10-phenanthroline and its derivatives using Ru-**C14j**.^552^

## 7 Conclusions and Outlook

The metal-catalyzed asymmetric hydrogenation of prochiral unsaturated amines has been intensively studied in the last ten years, and it continues to be a growing field and a fundamental tool in synthetic organic chemistry. More than four hundred articles have been published since 2010. Although a huge number of catalysts have been described to date, there are some privileged ligands, such as DuanPhos, SegPhos, and ZhaoPhos, able to provide excellent results in a large variety of substrates. Moreover, some modular catalytic systems such as JosiPhos, WalPhos, and the ruthenium DPEN have also proved highly versatile, through fine-tuning the substituents and counterions. In the last ten years we have witnessed incredible advances and may conclude that asymmetric hydrogenation is, arguably, the cleanest and most convenient methodology for the synthesis of chiral amines. Nevertheless, to overcome the fierce competition of biocatalytic and resolution methodologies, further improvement is needed. In the years to come we might expect further development on the use of nonprecious metals to allow for greener and cheaper processes. On the other hand, catalytic systems that are active and resistant to basic and/or acidic conditions, catalyst deactivation, or poisoning by amines are much needed. These advances and others which we may not foresee now will for sure keep the metal-catalyzed synthesis of chiral amines as a thriving field in the next decade.

## Abbreviations

AH: asymmetric hydrogenation
Alk: alkyl
ARA: asymmetric reductive amination
BAr^F^: tetrakis[3,5-bis(trifluoromethyl)phenyl]borate
BCDMH: 1-bromo-3-chloro-5,5-dimethylhydantoin
BINAP: 2,2′-bis(diphenylphosphino)-1,1′-binaphthyl
Boc: *tert*-butoxycarbonyl
Cbz: benzyloxycarbonyl
COD: cyclooctadiene
Cp*: pentamethylcyclopentadienyl
CSA: camphor-10-sulfonic acid
Cy: cyclohexyl
d-DTTA: d-di-p-toluoyl-d-tartaric acid
DCE: dichloroethane
DHIQs: dihydroisoquinolines
DHPDs: dihydrophenanthridines
DFT: density functional theory
DMF: dimethylformamide
DPEN: *trans*-1,2-diphenylethylene diamine
dr: diastereomeric ratio
EDG: electron-donating group
ee: enantiomeric excess
EWG: electron-withdrawing group
HA: chiral phosphoric acid
HFIP: hexafluoroisopropanol
MIBK: methyl isobutyl ketone
MOM: methoxymethyl ether
MS: molecular sieves
Ms: methanesulfonyl (mesyl)
nbd: norbornadiene
NHC: *N*-heterocyclic carbene
NIS: *N*-iodosuccinimide
PG: protecting group
PMB: *p*-methoxybenzyl
PMP: *p*-methoxyphenyl
Rf: perfluoroalkyl
RT: room temperature
S/C: substrate/catalyst ratio
tAA: *tert*-amyl alcohol
TBAI: tetrabutylammonium iodide
TBDPS: *tert*-butyldiphenylsilyl
TBS: *tert*-butyldimethylsilyl
TCCA: trichloroisocyanuric acid
TCFP: trichickenfootphos
Tf: trifluoromethanesulfonyl (triflate)
TFA: trifluoroacetic acid
TFE: trifluoroethanol
THF: tetrahydrofuran
THIQs: tetrahydroisoquinolines
THQs: tetrahydroquinolines
TMS: trimethylsilyl
TON: turnover number
Ts: *p*-toluenesulfonyl (tosyl)
Xyl: xylyl (dimethylphenyl)
